# Evolving patterns of co-mutations from tumor initiation to metastatic progression

**DOI:** 10.1038/s41588-026-02661-4

**Published:** 2026-07-10

**Authors:** Arvind Iyer, Miljan Petrovic, Debora Sesia, Luca Nanni, Marco Mina, Giovanni Ciriello

**Affiliations:** 1 Department of Computational Biology, University of Lausanne, Lausanne, Switzerland; 2 Swiss Cancer Center Leman, Lausanne, Switzerland; 3 Swiss Institute of Bioinformatics, Lausanne, Switzerland; 4 Present Address: HAYA Therapeutics SA, Epalinges, Switzerland

**Keywords:** Genome informatics, Genomics, Cancer

## Abstract

Phenotypically healthy cells frequently harbor somatic variants at cancer-associated genes, indicating that malignant transformation requires the selection of several alterations. Predicting which combinations of mutations, or co-mutations, exhibit oncogenic capacity requires identifying co-mutations that occur more or less frequently than expected. However, statistical frameworks to solve this problem are hampered by tumor heterogeneity and data availability. Here we curated putative oncogenic mutations in >70,000 human tumors from 119 subtypes, and designed a strategy to search for co-mutations based on in silico simulation of mutagenesis (SelectSim). Using this dataset and tool, we discovered and validated co-mutations across independent human cohorts, compared co-mutations across different tumor types and identified potential risk factors of metastatic progression. Notably, across several cohorts of phenotypically normal tissue samples, we show that, unlike individual oncogenic variants, significantly co-occurring mutations are largely cancer-specific and are observed rarely in healthy tissues, providing clues about the paths to tumorigenesis.

## Abstract (web summary)

SelectSim uses cancer and healthy tissue sequencing datasets to identify co-mutations that occur more or less frequently than would be expected by chance. Differences in co-mutation patterns observed during different stages of tumor evolution are also explored.

## Main

Somatic mutagenesis is one of the main enabling mechanisms of cancer initiation and evolution^1^. Nevertheless, somatic mutations, even bona fide cancer-associated variants, have been reported in several normal tissues^2–4^, supporting the notion that such variants alone are insufficient to drive tumorigenesis. Indeed, human tumors frequently exhibit several oncogenic mutations^5^, whose combined action is required for malignant transformation^6,7^. Work from our group and others have shown that not all combinations of somatic mutations are equally likely to be observed, indicating that only specific combinations exhibit oncogenic capacity and are selected during tumor evolution^8–10^.

Evidence of selected combinations comes from nonrandom patterns of co-occurrence and mutual exclusivity among somatic mutations. Co-occurring mutations may enable each other’s selection directly, as for alterations of the RB and p53 pathways^11–14^, or synergize indirectly by promoting complementary phenotypes, such as co-occurring mutations in the Wnt and ERK signaling pathways in colorectal cancer^15^. Conversely, other mutations are rarely or never found in the same tumors, either because they affect the same cellular process redundantly, as for mutually exclusive alterations within the cell cycle or ERK pathways^5,14^, or because they induce antagonistic effects, which may give rise to synthetic lethal interactions^16,17^. Overall, compelling evidence indicates that oncogenic phenotypes emerge from the concerted action of selected co-mutations.

The search for oncogenic co-mutations is the logical next step to the search for individual oncogenic or ‘driver’ mutations, and it faces similar challenges. In particular, identifying cancer driver genes has relied mainly on estimating statistically significant recurrent mutations^18,19^. This strategy requires several large datasets for discovery and validation, and to account for factors that influence mutation frequency independently of oncogenic selection^4,18–22^. The requirement of large tumor cohorts is even more pressing for co-mutations given the expected low number of samples exhibiting two or more mutations within a specific geneset. As a result, previous studies have limited their scope to representative tumor types^23,24^ or resorted to aggregating several cohorts in so-called pancancer cohorts^14,25^. Even so, validation of co-mutations across independent datasets is still largely missing. Moreover, mutation co-occurrence and mutual exclusivity may emerge because of highly variable gene and tumor mutation rates, lineage-specificity, genomically different tumor subtypes and heterogeneous mutational processes^10^. These mechanisms are independent of synergistic, redundant or antagonistic interactions and ignoring them leads to misinterpreting the biological relevance of co-mutations in cancer. Currently, only a few approaches can account for several tumor types and subtypes in the same dataset or heterogeneous tumor mutation rates, and none can correct for the influence of heterogeneous mutational processes^10^. Approaches to search for co-mutations rely typically on either well-defined probability distributions, which can cope with large numbers of samples and hypotheses but poorly account for the many confounders, or empirical models of co-mutations based on data permutation, which provide more flexibility to incorporate several covariates, although their statistical power is bounded by the number of permutations, which are time-consuming, especially for large datasets.

Here we introduced a new strategy based on in silico simulation of mutagenesis, which retains the flexibility of empirical methods while overcoming the need for inefficient permutations. Our approach outperformed existing methods, and it allows controlling for heterogeneous mutational processes and mutation rates among different tumors and genes, while preserving genomic diversity of tumor types and subtypes. With this strategy in place, we harmonized patient annotations and mutation data across >70,000 human tumors to discover and validate co-mutations across several independent human cohorts, compare co-mutations among different tumor types and between primary tumors and metastases, and explore co-mutation incidence in normal tissues.

## Results

### In silico simulation of mutagenesis

**Fig. 1:**
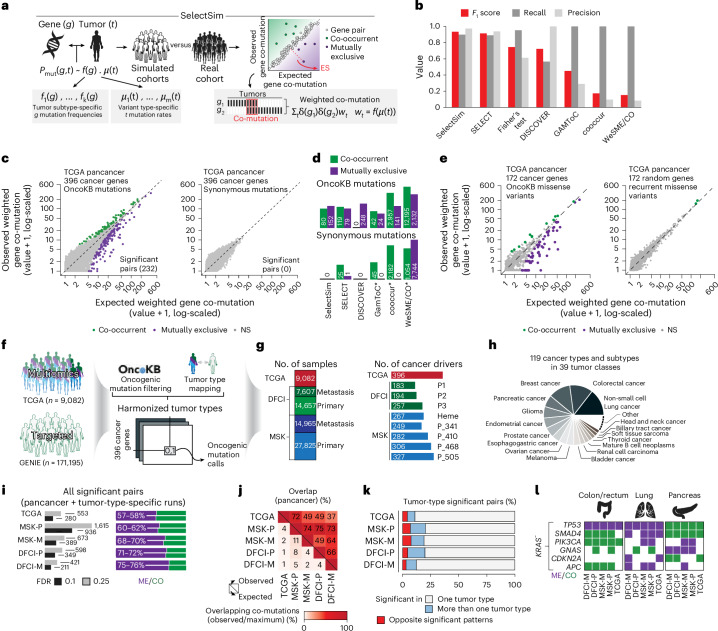
Discovering functional co-mutations through in silico simulation of cancer genomes. **a**, Graphical schematic of the SelectSim algorithm. **b**, Precision, recall and *F*_1_ score obtained by each benchmarked tool on an artificial dataset comprising 1,000 samples, 100 genes and 15 true co-occurrent and 15 true mutually exclusive mutated gene pairs. **c**, Scatterplots comparing the expected (*x* axis) versus observed (*y* axis) weighted gene co-mutation incidence computed by SelectSim in the TCGA pancancer dataset retaining either mutations classified as oncogenic or probably oncogenic in OncoKB (left) or synonymous (right) variants. **d**, Number of significant co-mutations detected by the methods indicated in the TCGA pancancer dataset retaining either mutations classified as oncogenic or probably oncogenic in OncoKB database (top) or synonymous mutations(bottom). Methods marked with asterisks do not account for different tumor types. **e**, Scatterplots comparing the expected (*x* axis) versus observed (*y* axis) weighted gene co-mutation incidence computed by SelectSim in the TCGA pancancer dataset retaining either missense mutations classified as oncogenic or probably oncogenic in OncoKB in 172 cancer-associated genes (left) or matching numbers of recurrent missense mutations in 172 randomly sampled genes (right). **f**, Schematic representation of the datasets integrated. For each dataset, we retained mutations classified as oncogenic or probably oncogenic in the OncoKB database for 396 cancer-associated genes and generated binary mutation matrices. **g**, Sample counts and distribution (left) and number of genes retained for each target gene panel (right) for the five tumor cohorts analyzed in detail. **h**, Pie chart of the distribution of the harmonized tumor classes defined for our analyses. **i**, Left: number of significant co-mutations detected by SelectSim across different cohorts based on FDR < 0.25 and FDR < 0.1 (-P, primary; -M, metastasis); right: proportions of ME and CO mutations. **j**, Expected (lower triangular matrix) and observed (upper triangular matrix) overlap percentage between sets of significant co-mutations in different cohorts. **k**, Fractions of gene pairs (per cohort) that are significantly co-mutated in one or more than one tumor type. **l**, Tumor type specificity of co-mutations involving *KRAS* and other genes in representative tissues. NS, nonsignificant.

**Extended Data Fig. 1:**
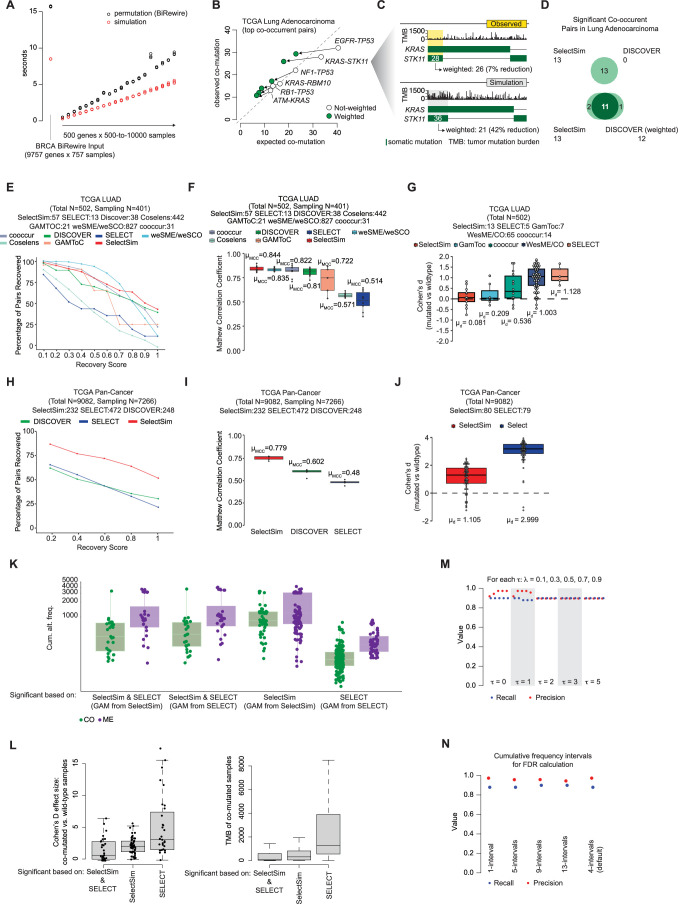
Benchmarking SelectSim. **A**) Time analysis comparison of simulation vs permutation approach using Breast Cancer samples. **B**) Expected (X-axis) vs observed (Y-axis) co-mutations for 6 co-mutations in lung adenocarcinoma. White points indicate unweighted co-mutation incidence, and green points are weighted co-mutation incidence. Arrows connect points corresponding to the same co-mutation. **C**) Highlight of *KRAS*-*STK11* co-mutation incidence in the observed (top) and simulated (bottom) dataset, represented as ‘oncoprint’, with genes as rows and samples as columns. Mutations for each gene are shown as green bars where individual patient mutations are collapsed. Tumor mutation burden (TMB) barplots are shown on the top (one value for each sample). The shaded areas highlight the co-mutated samples (that is, samples harboring both mutations). **D**) Venn-diagram of significant hits detected by SelectSim, DISCOVER, and modified DISCOVER in the TCGA lung adenocarcinoma (LUAD) dataset. **E** and **H**) Line graph of recovery score vs. percentage of pairs recovered for tested de-novo tools using the TCGA LUAD dataset (**E**) and the TCGA pan-cancer dataset (**H**). **F** and **I**) Box plot comparison of Mathew correlation coefficients (MCC) in the TCGA LUAD (**F**) and TCGA pan-cancer datasets (**I**) for tested de-novo tools. Box plot legend: centre line = median, bounds of the box = 25th and 75th percentiles, whiskers’ ends = 25^th^ percentile – 1.5 x IQR and 75^th^ percentile + 1.5 x IQR. **G** and **J**) Box plot comparison of Cohen’s d effect size distributions in the TCGA LUAD (**G**) and TCGA pan-cancer datasets (**J**) for tested de-novo tools. Box plot legend: centre line = median, bounds of the box = 25th and 75th percentiles, whiskers’ ends = 25^th^ percentile – 1.5 x IQR and 75^th^ percentile + 1.5 x IQR. **K**) Box plots of cumulative gene alteration frequencies for significant co-mutations obtained by SELECT and SelectSim, or exclusively by each of the two methods. The frequencies for co-mutations detected by both methods are calculated twice, for each of the two GAMs used in the respective methods. Box plot legend: centre line = median, bounds of the box = 25th and 75th percentiles, whiskers’ ends = 25^th^ percentile – 1.5 x IQR and 75^th^ percentile + 1.5 x IQR. **L**) Box plots of tumor mutational burden of samples with co-mutations (right), and box plots of Cohen’s D effect size (left) comparing the burdens of samples with co-mutations with the burdens of wild-type samples. The box plots split the significant co-mutations between those obtained by SELECT and SelectSim, or exclusively by each of the two methods. Box plot legend: centre line = median, bounds of the box = 25th and 75th percentiles, whiskers’ ends = 25^th^ percentile – 1.5 x IQR and 75^th^ percentile + 1.5 x IQR. **M** and **N**) Precision (red) and recall (blue) values obtained by SelectSim using different lambda and tau parameters (**M**), as well as changing the frequency intervals for FDR calculation (**N**). All analyses were performed using our ground truth artificial dataset with 30 evolutionary dependent pairs and ε = 0.95. Default intervals were used when varying lambda and tau, and default lambda = 0.3 and tau = 1 values were used when varying frequency intervals.

To search for co-mutations (co-occurrence and mutual exclusivity) in large and heterogeneous tumor cohorts, we (1) simulate mutation datasets under the hypothesis of independent selection of individual mutations and (2) estimate statistically significant co-mutations from the comparison of simulated and real mutation patterns (SelectSim). Briefly, SelectSim estimates the expected number of mutations in a given gene g and sample *t* from the mutation frequency of the gene, f(g), and the relative tumor mutation burden (TMB) of the sample, μ(t). These values are estimated within specific mutation and tumor subsets to account for heterogeneous tumor types, tissue specificities and distinct mutational processes. SelectSim uses these estimates to simulate gene mutations in a set of samples under the assumption of independence among genes and samples, while preserving gene mutation frequencies and TMB (Methods), and reducing the computing time with respect to data permutations (Extended Data Fig. 1a). Finally, for each gene pair $({g}_{1},{g}_{2})$, SelectSim determines the distance between the co-mutation incidence of $({g}_{1},{g}_{2})$ in the real and simulated datasets (effect size (ES)), where the co-mutation incidence is defined as the number of samples exhibiting mutations in ${g}_{1}$ and ${g}_{2}$, weighted by the relative TMB of each sample (Methods). Gene pairs with absolute normalized ESs exceeding the threshold corresponding to a false discovery rate (FDR) < *α* are considered statistically significant (Fig. 1a). In this study, we set *α* = 0.1 or 0.25 (Methods).

Note that co-mutation incidences have been estimated typically by counting the number of samples exhibiting mutations in two genes. However, this overestimates the significance of mutually exclusive mutations^9^ and underestimates the significance of co-occurrent mutations^23^. SelectSim weights each sample as a function of its TMB, based on the assumption that *n* co-mutated samples with low TMBs are more surprising than *n* co-mutated samples with high TMBs. We tested this strategy for known co-occurring mutations in lung adenocarcinoma^8^, such as co-mutations of *KRAS* and *STK11*. In The Cancer Genome Atlas (TCGA)^5^ lung adenocarcinoma dataset (TCGA LUAD, *n* = 502 samples), unweighted co-mutation incidences were lower than expected, indicating a trend for mutual exclusivity (Extended Data Fig. 1b, white points), but with our weighting strategy, these pairs were found correctly by SelectSim to be significantly co-occurrent (Extended Data Fig. 1b,c, green points). A previous method, DISCOVER, that uses a similar approach to SelectSim to estimate the likelihood of a gene being mutated in a specific sample reported lack of co-occurrent mutations in cancer^23^. However, upon applying our weighting strategy to DISCOVER, most co-occurrences were recovered (Extended Data Fig. 1d), demonstrating that overestimated co-mutations in samples with high TMB explains the lack of significant co-occurrence reported previously^23^.

Next we benchmarked SelectSim against approaches developed recently that are able to detect both mutually exclusive and co-occurrent alterations^14,23,24,26–28^ (Supplementary Table 1) on real and synthetic datasets. On the TCGA LUAD dataset, SelectSim stood out for its robustness in recovering the same significant hits across several subsampled runs (Extended Data Fig. 1e,f) and for its ability to account for heterogeneous mutation rates, as shown by the lowest dependency between significant co-mutations and TMBs (Extended Data Fig. 1g). SelectSim’s top performances were confirmed in the TCGA pancancer cohort (n = 9,082) against the only two approaches that allow to account for heterogeneous tumor types in the same dataset: SELECT and DISCOVER (Extended Data Fig. 1h–j). In contrast to SelectSim, significant co-mutations that were discovered exclusively by SELECT involved gene pairs that exhibited low alteration frequencies and that were co-mutated in samples with high TMBs (Extended Data Fig. 1k,l), suggesting these are false positive hits driven by heterogenous TMBs. To assess the ability of different methods to recover ‘true’ co-mutations, we designed a model to generate 1,000 synthetic tumor samples harboring a variable number of mutations across 100 genes, with a predefined set of 15 synergistic and 15 antagonistic gene pairs. In our model, synergistic and antagonistic interactions modulate the probability of mutating a gene once its interacting partner is mutated, leading to the emergence of co-occurrent and mutually exclusive mutations, respectively (Methods). On this dataset, only SelectSim and SELECT exhibited both good precision and recall, with SelectSim showing marginally superior performances (Fig. 1b), which remained robust across a wide range of parameter values (Extended Data Fig. 1m,n). In contrast, all methods except DISCOVER exhibited good recall but poor precision, consistent with a high number of false positives, whereas DISCOVER correctly identified the mutual exclusivity pairs but failed to recover true co-occurrent mutations.

**Extended Data Fig. 2:**
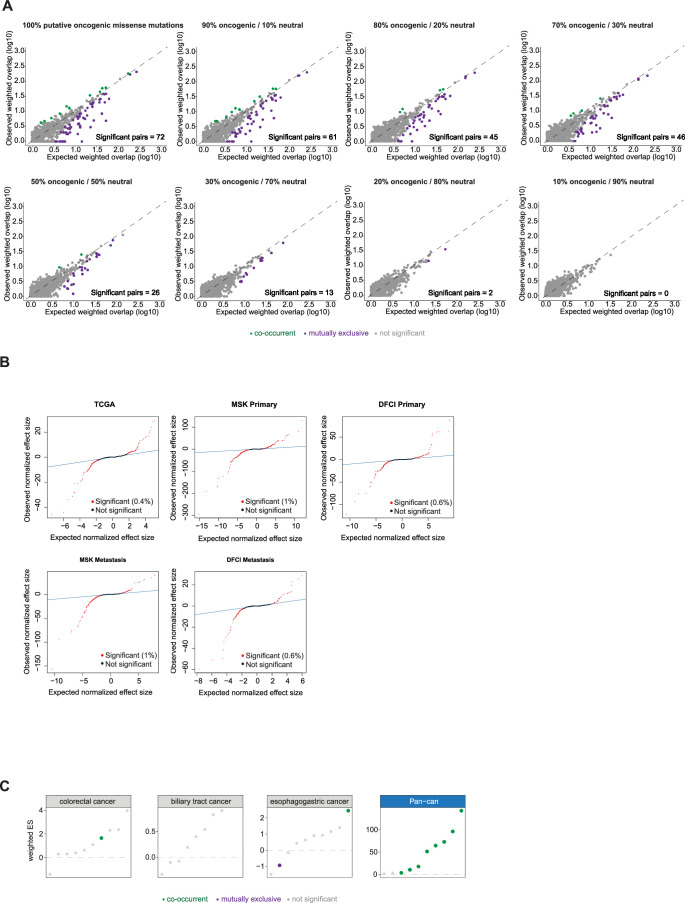
Assessment of weighted effect sizes of SelectSim. **A**) Observed weighted co-mutation (x-axis) vs Expected weighted co-mutation (y-axis) across 8 datasets derived from TCGA comprising different proportions of oncogenic and neutral mutations for the same set of genes. Significant co-mutations called by SelectSim are green (co-occurrence) and purple (mutual exclusivity). **B**) Q-Q plots of SelectSim’s weighted effect size of co-mutations (wES) against the normal distribution, for each of the 5 analyzed datasets (TCGA, MSK-P, DFCI-P, MSK-M, and DFCI-M). **C**) Weighted effect sizes of co-mutation of *APC* and *TP53* across colorectal, biliary tract, esophageal cancer, and pan-cancer analysis. Significant co-mutations are color-coded according to the legend. Multiple points per tumor types indicate results coming from different datasets and gene panels where the co-mutation was testable.

Co-mutations identified by SelectSim were not affected by tumor purity and clonality (Supplementary Note), and were associated exclusively with oncogenic variants. Indeed, among 396 cancer-associated genes in the TCGA pancancer cohort, SelectSim detected numerous significant mutually exclusive and co-occurrent mutations when we retained mutations annotated as oncogenic by OncoKB^29^, but none when we retained synonymous mutations (Fig. 1c). In contrast, tools that did not account for heterogenous tumor types detected an abnormally high number of significant co-mutations, probably inflated by false positives, and all except for DISCOVER detected significant co-mutations even among synonymous variants (Fig. 1d). To assess further the influence of neutral mutations on co-mutation discovery, for each gene we retained a mix of putative oncogenic and neutral variants. The number of significant co-mutations detected by SelectSim decreased as the percentage of neutral variants increased, with 20% neutral mutations leading to a reduction of significant co-mutations of ~40% (Extended Data Fig. 2a and Supplementary Note). Finally, on subsets of either cancer-associated or randomly sampled genes with matched number of missense variants, SelectSim detected significant co-mutations only among cancer-associated genes (Fig. 1e). Overall, these results demonstrate that co-mutations detected by SelectSim are driven by evolutionary selection of functional variants.

### A co-mutation atlas across human cancers

To discover and validate co-mutations across several independent cohorts, we collected and curated mutation data for 171,195 tumor samples from the Genomics Evidence Neoplasia Information Exchange (GENIE) dataset^30,31^. For all these datasets, we retained putative oncogenic mutations and built binary alteration matrices (Fig. 1f and Supplementary Data 1). However, given the heterogeneity of sequencing pipelines, gene panels, and clinical annotations, we decided to focus on the two largest single-center cohorts in GENIE: the Memorial Sloan Kettering Cancer Center (MSK) and Dana Farber Cancer Institute (DFCI) datasets (Supplementary Data 2). Upon filtering samples that did not meet our selection criteria (Methods), we retained 27,825 primary (MSK-P) and 14,965 metastatic (MSK-M) samples in the MSK cohort, and 14,657 primary (DFCI-P) and 7,607 metastatic (DFCI-M) samples in the DFCI cohort (Fig. 1g).

Including the TCGA cohort, we curated mutation data for 74,136 tumor samples and 396 cancer-associated genes, or the subsets of these 396 genes that were sequenced in the MSK and DFCI cohorts (Supplementary Table 2), retaining only mutations that were classified as ‘oncogenic’ or ‘likely oncogenic’ in OncoKB^29^ (Fig. 1g and Supplementary Table 3). Finally, we harmonized clinical annotations, resulting in 39 tumor classes and 119 tumor (sub)types (Fig. 1h and Supplementary Table 4). We ran SelectSim on all five main datasets, both aggregating all tumor classes (pancancer) or within each tumor class, using tumor (sub)types as covariates (Fig. 1i, Extended Data Fig. 2b and Supplementary Table 4). Results were consistent across datasets (Fig. 1j and Supplementary Table 5) and co-mutations frequently exhibited tissue-specificity, with less than a quarter of co-mutations found significant in two or more tumor classes (Fig. 1k). Furthermore, we identified instances of co-mutations that were significant in several tumor classes although exhibiting opposite patterns (that is, mutual exclusivity or co-occurrence). Representative cases involved the *KRAS* oncogene, which was, for example, co-mutated with *TP53* less frequently than expected in colorectal and lung cancer, but more frequently than expected in pancreatic cancer, across all five datasets (Fig. 1l).

**Fig. 2:**
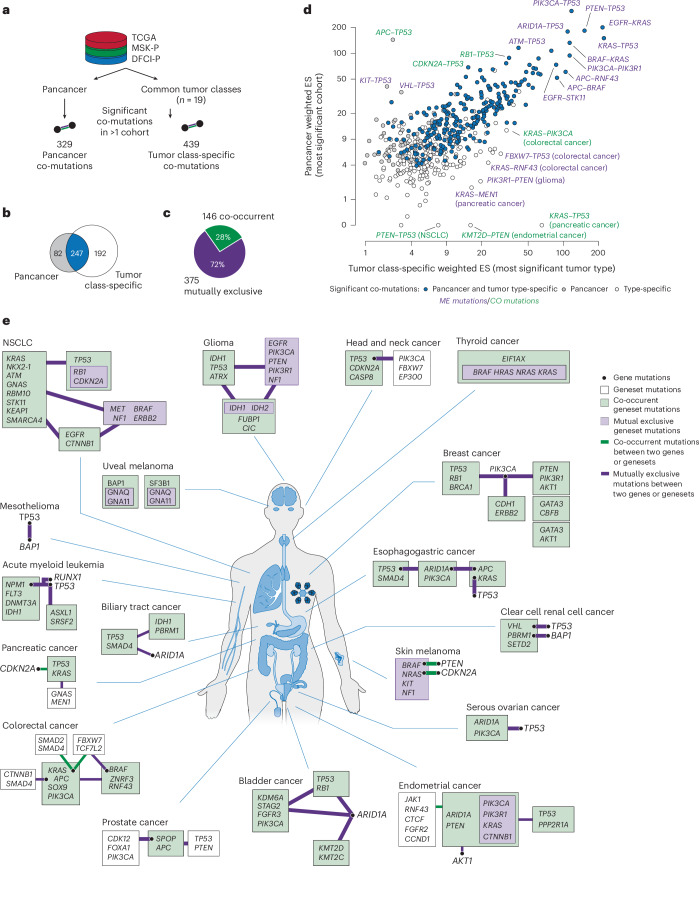
Co-mutation atlas. **a**, Schematic representation of the analysis: out of three primary tumor cohorts, we identified significant pancancer and tumor class-specific co-mutations. We retained 329 pancancer and 439 tumor class-specific co-mutations that were found in at least two independent datasets. **b**, Venn diagram representation of the intersection between pancancer and tumor class-specific co-mutations. **c**, Pie chart of the distribution of co-occurrent (green) and mutually exclusive (purple) co-mutations. **d**, For each significant co-mutation validated in independent datasets, either pancancer or tumor class-specific, we report the maximum weighted effect size achieved in the tumor class-specific analyses (*x* axis) or pancancer analyses (*y* axis). Each point corresponds to a co-mutated gene pair, and it is color-coded based on the analysis in which it was found significant in independent datasets (white: tumor class-specific only (*n* = 192); gray: pancancer only (*n* = 82); orange: tumor class-specific and pancancer (*n* = 247)). Representative co-mutations are labeled, with label colors corresponding to the co-mutation pattern (green: co-occurrence, purple: mutual exclusivity). **e**, Schematic representations of significant tumor class-specific co-mutations for 18 tumor types where significant co-mutations were found. Each box represents a geneset. Green boxes: genesets exhibiting co-occurrent mutations. Purple boxes: genesets exhibiting mutually exclusive mutations. Black dots are associated to individual genes. Connections between genesets and/or individual genes indicate that the corresponding genesets and/or individual genes are mutated in a co-occurrent (green) or mutually exclusive (purple) manner. NSCLC, non-small cell lung cancer.

To validate co-mutations across independent cohorts, we focused on 19 tumor classes that were represented within the TCGA, MSK and DFCI primary tumor cohorts (Supplementary Table 6), and retained pancancer and tumor class-specific co-mutations that were significant in at least two independent datasets. In total, we identified 329 pancancer and 439 tumor class-specific independently validated co-mutations (Fig. 2a and Supplementary Table 7), 247 of which were in overlap (Fig. 2b), with 72% mutually exclusive and 28% co-occurrent co-mutations (Fig. 2c). Pancancer and tumor class-specific effect sizes were overall correlated (Spearman’s correlation = 0.63; Fig. 2d) and they were higher when co-mutations were significant in both pancancer and tumor class-specific cohorts. Co-mutations that differed most between pancancer and tumor class-specific cohorts highlighted either lineage-specific selection, such as co-occurrent mutations of *KRAS* and *TP53* in pancreatic cancer, or co-mutations common to several tumor classes but at a frequency insufficient to reach statistical significance. For example, co-occurrent mutations of *APC* and *TP53* were observed across biliary tract, colorectal and esophageal cancer, but with modest effect sizes (Extended Data Fig. 2c), whereas, once combined in pancancer cohorts, they scored among the top significant co-mutations (Fig. 2d).

**Extended Data Fig. 3:**
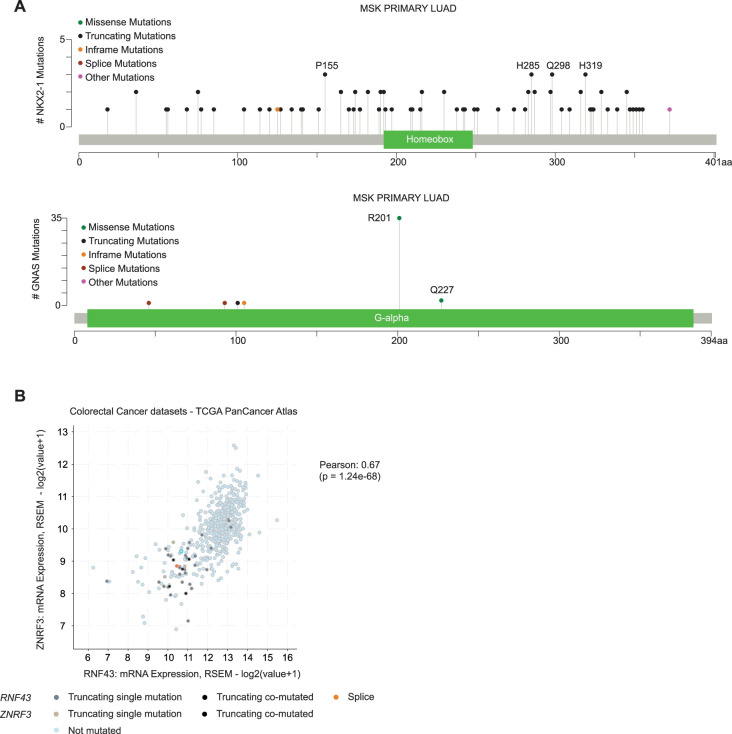
Supporting evidence for specific co-mutation-driven trajectories. **A**) Putative oncogenic somatic mutations detected for *NXK2-1* (top) and *GNAS* (bottom) in the MSK-P dataset. Mutations are positioned as in the protein sequence (X-axis) and the height of each bar corresponds to the mutation incidence in the cohort (Y-axis). **B**) A scatter plot of log normalized RSEM expression value of *RNF43* and *ZNFR3* genes in the TCGA colorectal cancer dataset (n = 352), where each point corresponds to a patient, and it is color-coded based on the *RNF43* and *ZNFR3* gene mutation status.

**Extended Data Fig. 4:**
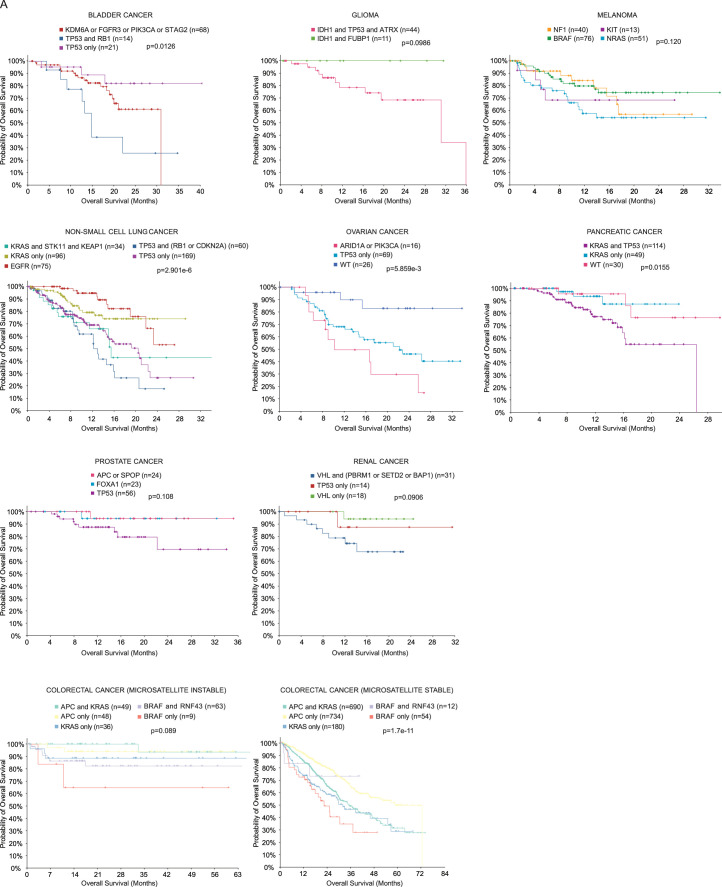
Effect of co-mutations on patient survival. **A**) Overall survival Kaplan-Meier curves for representative tumor types of the MSK dataset. The arms of the cohorts correspond to patients with particular mutations or co-mutations as labeled in the legends. The p-values of the two-tailed log-rank test are reported. Sample sizes of each arm are given in the panel legends.

Among these co-mutations, we searched for co-mutated genesets characteristic of each tumor type (Fig. 2e). Co-mutated genesets often highlighted distinct evolutionary trajectories of the disease, with each trajectory defined by co-occurring mutations (Fig. 2e, green boxes) and mutual exclusivity naturally defining the boundaries between distinct trajectories (Fig. 2e, purple lines). For example, in non-small cell lung cancer, we found a trajectory of co-mutations centered on *KRAS* mutations, which co-occurred with loss-of-function (LoF) mutations of *STK11* and *RBM10*, as reported previously^8,14^, but also with LoF mutations of *NKX2-1* and hotspot mutations of *GNAS* (Extended Data Fig. 3a). *NKX2-1* encodes TTF1—a lineage factor of alveolar cells^32^, the cell of origin of lung adenocarcinoma. *NKX2-1* loss in normal alveolar type-2 cells gives rise to a Krt8^+^ progenitor state^33^, which has been associated with lung tumorigenesis^34^ and, in tumor cells, *NKX2-1* is downregulated with progression of the disease, leading to dedifferentiated cancer cell states^35^. LoF mutations in *NKX2-1* may thus be promoting a dedifferentiated cell state in the early phase of the disease, synergizing preferentially with *KRAS* oncogenic variants^36^. *GNAS* codon 201 hotspot mutations have been reported in gastric, pancreatic and colorectal cancers, often concomitantly with *KRAS*-activating mutations^37,38^. The significant co-occurrence identified by SelectSim indicates that a similar synergy may also exist in lung adenocarcinoma. Mutually exclusive to the KRAS-trajectory, we found mutations of genes encoding other key MAPK regulators such as *BRAF* or *NF1*, receptor tyrosine kinases *MET* and *ERBB2* and, especially, *EGFR*, which was the second most frequently mutated oncogene in lung adenocarcinoma after *KRAS*. *EGFR* mutations co-occurred significantly with mutations of *CTNNB1*, which have been associated previously with therapeutic resistance in this cancer type^39^. Beyond lung cancer, evolutionary trajectories were highlighted by co-mutations in bladder and colorectal cancer. In bladder cancer, strong co-occurrence of *FGFR3*, *STAG2*, *KDM6A* and *PIK3CA* mutations defined one principal axis, which was significantly mutually exclusive with co-occurring *TP53* and *RB1* mutations. *TP53* and *RB1* co-mutations have been reported as markers of resistance to immunotherapy in bladder cancer^40^, and they predispose to transdifferentiation to small cell carcinoma in several cancer types^41^, including emerging evidence in bladder cancer^42^. This co-mutation may thus define a subpopulation of high-risk patients in this (but not only this) tumor type. In colorectal cancer, the prototypical *APC–KRAS* co-mutation^6^ was found further co-occurring with activating mutations of *PIK3CA* and LoF mutations of genes encoding transforming growth factor β signal transducers *SMAD2* and *SMAD4*, and the transcription factor *SOX9*, which are enriched in microsatellite stable tumors^5^. Conversely, tumors exhibiting microsatellite instability were enriched for co-occurring mutations in *BRAF* and in paralogs *RNF43* and *ZNRF3*, which encode transmembrane E3 ubiquitin ligases responsible for ubiquitinating and degrading Wnt receptors^43^. Although the extent to which their individual loss can activate Wnt signaling has been questioned^44^, co-occurring LoF mutations at these genes may indicate a synergistic effect upon their double loss, as supported by evidence of their concomitant downregulation in a subset of colorectal tumors (Extended Data Fig. 3b). Across several tumor types, co-mutations provided prognostically relevant patient stratifications (Extended Data Fig. 4). For example, co-occurrence of *TP53* and either *RB1* or *CDKN2A* mutations was associated with worse prognosis in both bladder and lung cancer, even when compared to other *TP53* mutant samples. Co-occurring mutations associated with worse prognosis included co-mutation in *KRAS* and *STK11* or *KEAP1* compared to other *KRAS*-mutated tumors, in lung cancer, *APC* and *KRAS* compared to other *APC*-mutated cases, in colorectal cancer, and *VHL* and *PBRM1*, *SETD2* or *BAP1*, compared to other *VHL*-mutated cases, in renal cancer (Extended Data Fig. 4). Similarly, mutually exclusive variants identified survival differences in several tumor types, such as between *KRAS* and *BRAF* mutated tumors in colorectal cancer, independently of microsatellite instability status, *KRAS* and *EGFR* in lung cancer and *BRAF* and *NRAS* in skin melanoma (Extended Data Fig. 4). Overall, we discovered significant co-mutations that were validated independently across several cohorts and that were representative of prognostically relevant evolutionary trajectories across several tumor types.

### Co-mutations as predictors of metastatic progression

**Fig. 3:**
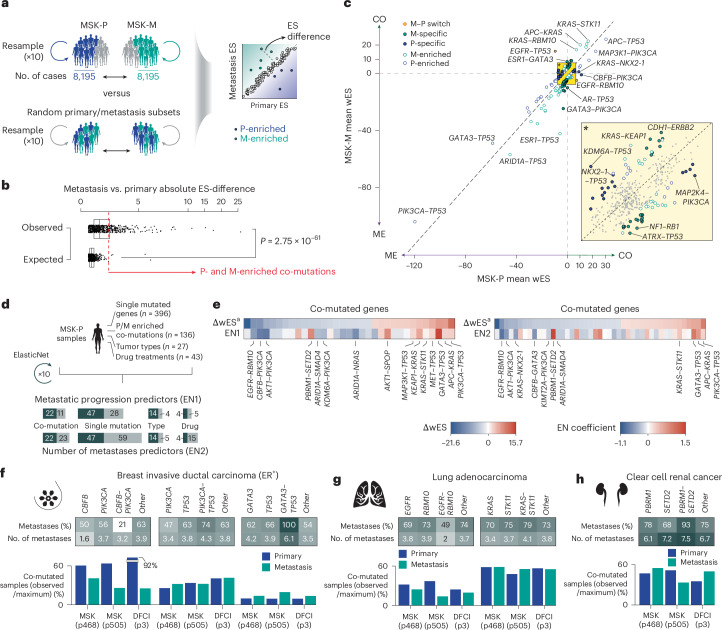
Co-mutations in metastatic versus primary tumors. **a**, Schematic representation of co-mutation comparison between primary (P) and metastatic (M) samples. **b**, Box plot comparison of the absolute value of the difference between weighted effect size (mean over ten runs) for each gene pair (*n* = 508) between P and M datasets (observed) and between randomly admixed datasets (expected). *P* value computed by two-tailed Wilcoxon test. In box plot, center line: median; box bounds: 25th and 75th percentiles; whisker ends: 25th percentile − 1.5× interquartile range (IQR) and 75th percentile + 1.5× IQR. **c**, Scatterplot comparing observed mean weighted ES (mean wES) in MSK primary tumors and observed mean wES in MSK metastases. Each point is a gene pair: P- or M-enriched: co-mutations with ΔwES >99th percentile of the expected distribution (see **b**). P- or M-specific: P- or M-enriched co-mutations significant in at least six out of ten runs in one cohort and in at most two runs in the other cohort. *EGFR–TP53* and *EGFR–RBM10* are highlighted in yellow as the only co-mutations recurrently significant in both cohorts, although with opposite patterns (M–P switch). Yellow box: enlarged version of the central part of the scatterplot; selected co-mutations are labeled. ΔwES: difference between mean wES values in primary tumors and metastasis. **d**, Schematic representation of the ElasticNet analysis highlighting the number and type of variables included in the model (top) and the resulting number of strong predictors that were retained (bottom), either by both models (dark green) or only one of the two models (light green). **e**, Heatmap of mean ΔwES and ElasticNet coefficients of co-mutations that predict metastatic progression (*n* = 33) and number of metastases (*n* = 45). Negative and positive ΔwES values indicate that co-mutations are enriched in P and M samples, respectively. Negative and positive ElasticNet coefficients indicate that co-mutations were negative or positive predictors of metastatic progression (left) or number of metastases (right). Selected co-mutations are labeled. **f**–**h**, For selected co-mutations, we report detailed information on breast (**f**), lung (**g**) and renal (**h**) cancer for the indicated tumor types and co-mutations. Top: percentage of MSK-P samples that developed metastases and mean number of metastases found in samples exhibiting either one, both or none of the specific mutations (heatmap with numeric values indicated). Bottom: percentage of samples exhibiting a given co-mutation (number of co-mutated samples divided by the maximum possible number of co-mutated samples, that is, the minimum of the two individual gene mutation counts) in P and M samples from three cohorts: two independent MSK cohorts analyzed with a different gene panel, and one DFCI cohort (barplots). ^a^Metastasis wES − Primary wES.

Next we investigated the consistency of co-mutations between primary and metastatic samples in MSK cohorts. We ran SelectSim on ten subsampled cohorts with the same number (*n* = 8,195) of primary (P) and metastatic (M) samples and distribution of tumor (sub)types, as well as on ten control cohorts of the same size, where P and M samples were mixed (Fig. 3a).

**Extended Data Fig. 5:**
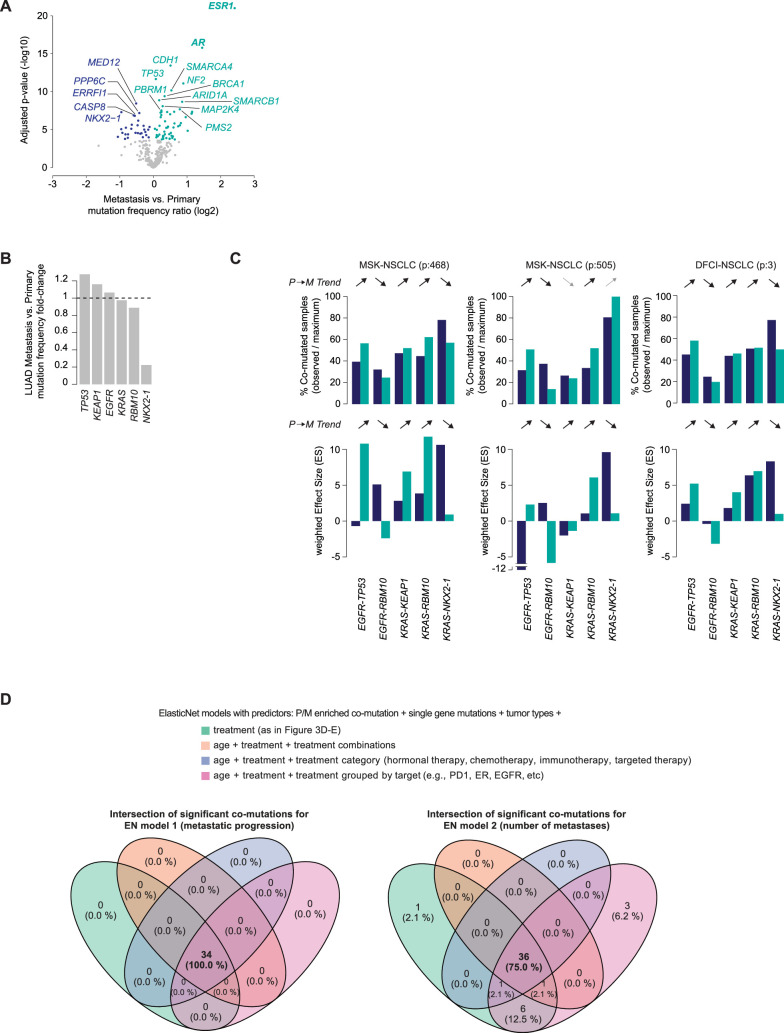
(Co-)mutation incidences between primary and metastatic samples. **A**) Volcano plot showing the fold-change (X-axis) and adjusted p-value (Benjamini-Hochberg, Wilcoxon two-tailed test, Y-axis) of individual gene mutation frequencies (n = 304) across primary and metastatic samples. Representative genes are labeled. **B**) Bar plot showing the gene mutation frequency fold change in the Lung adenocarcinoma dataset of primary vs. metastatic samples. **C**) Top: Bar plots of the percentage of co-mutation among pairs of genes in primary (dark blue) and metastatic (light blue) samples in non-small cell lung cancer in MSK (p468), MSK (p505), and DFCI (p3) dataset. Bottom: Bar plots of the co-mutation weighted effect size among pairs of genes in primary and metastatic samples in non-small cell lung cancer in MSK (p468), MSK (p505), and DFCI (p3) dataset. The arrows above each barplot indicate the direction of change: upward arrow for increasing percentage/wES from primary to metastasis, downward arrow for decreasing percentage/wES. Thin gray arrows are used to indicate directions of change that are inconsistent with the majority observed. **D**) Venn diagrams of co-mutations with strong predictive power of metastatic progression (left) or number of metastases (right) across models that include different groups of treatments as variables.

Strikingly, differences between the mean co-mutation effect sizes in P and M samples (ΔwES) were significantly higher than those in the control cohorts (Fig. 3b), indicating that co-mutations in primary tumors and metastases differed more than expected. Some of the co-mutations that were significant only among metastatic samples (M-specific) were associated with genes that were mutated more frequently in metastatic samples (Extended Data Fig. 5a), such as genes encoding for the estrogen and androgen receptors *ESR1* and *AR* (Fig. 3c), consistent with their mutations driving resistance to therapy and tumor relapse^45–47^. Among strong co-mutation differences (Supplementary Table 8) that were independent of individual gene mutation frequencies, we found co-mutations involving *EGFR*. *EGFR–RBM10* mutations were significantly co-occurrent in primary samples but significantly mutually exclusive in metastases. *RBM10* mutations exhibited instead a stronger co-occurrence with *KRAS* mutations in metastases compared to primary samples, suggesting differential synergies of *RBM10* mutations in the two genetic contexts. These genes were mutated with a similar frequency in P and M samples, especially in lung adenocarcinoma (Extended Data Fig. 5b), where these co-mutations were enriched and where they exhibited the same trends in MSK and DFCI independent cohorts (Extended Data Fig. 5c). Other significant differences involved *TP53* mutations, which were only marginally more abundant in metastases than in primary samples (48% in M versus 46% in P). For example, *EGFR–TP53* co-mutations were significantly mutually exclusive in primary samples but co-occurrent in metastases. In most cases, co-mutation effect sizes were correlated (Fig. 3c), indicating that significant differences rarely amounted to a complete change of co-mutation patterns but rather to an increase or reduction of the same co-mutation pattern. For example, although remaining less frequent than expected, co-occurring mutations of *TP53* and either *GATA3* or *PIK3CA* were more abundant in metastases, whereas the incidence of co-occurring mutations of *TP53* and *ARID1A* further decreased in metastatic disease (Fig. 3c). These observations may be indicative of a different metastatic risk in tumors exhibiting specific co-mutations.

To test this hypothesis, we asked whether co-mutations in primary samples were predictive of metastatic progression. Leveraging follow-up data available for the MSK primary cohort^48^, we implemented a machine learning strategy based on penalized regression (ElasticNet) to predict (1) whether metastatic progression had occurred or not and (2) how many metastases were detected (Fig. 3d). In total, we analyzed 12,925 primary tumor samples, 74% of which ultimately metastasized. Beyond co-mutations, we included in our model individual gene mutations, the tumor type of origin and treatment information^49^ (Supplementary Table 9).

For several co-mutations, the differences between P and M samples (ΔwES) and ElasticNet (EN) coefficients were concordant (Fig. 3e) and, importantly, co-mutations retained their predictive power even when accounting for age and treatment (Extended Data Fig. 5d). For example, co-mutations of *TP53* and either *PIK3CA* or *GATA3* were significantly more frequent in metastatic samples, as highlighted by the high ΔwES values and, when observed in primary tumors, were strong predictors of both metastatic progression and high number of metastases, as highlighted by the positive EN coefficients (Fig. 3e). In breast cancer, where these mutations are frequent^50^, primary tumors exhibiting *PIK3CA–TP53* or *GATA3–TP53* co-mutations had a higher incidence of metastatic progression (74% versus 58% for *PIK3CA–TP53*, and 100% versus 61% for *GATA3–TP53*) and had a higher number of metastases (Fig. 3f, heatmaps). Consistently, the percentage of co-mutated samples was higher among metastases than primary samples in both MSK cohorts and the independent DFCI cohort (Fig. 3f, barplots). Conversely, *PIK3CA–CBFB* co-mutations were more frequent in primary samples and a negative predictor of metastatic progression and number of metastases (Fig. 3e,f). *KRAS–STK11* and *KRAS–KEAP1* co-mutations were enriched in metastases (Fig. 3c) and positive predictors of metastatic progression, although with a moderate effect size (Fig. 3e). These trends were confirmed in lung adenocarcinoma (Fig. 3g), where these co-mutations are frequent^8^. Conversely, *EGFR–RBM10* co-mutations were enriched in primary samples in both MSK and DFCI lung adenocarcinoma cohorts and were negative predictors of metastatic spread (Fig. 3g). Finally, we observed a contradicting trend for *PBRM1–SETD2* co-mutations, which were enriched in primary tumors but positive predictors of metastatic progression (Fig. 3e). In clear cell renal cancer, where these mutations are most frequent^51^, we observed that the enrichment in primary tumors was observed only in one of the MSK datasets. In contrast, in the other MSK cohort and in the DFCI cohort, *PBRM1–SETD2* co-mutations were enriched in metastases, concordantly with ElasticNet predictions, and renal tumors harboring such co-mutation had a higher incidence of metastatic progression (93% versus 74%) (Fig. 3h). Overall, co-mutations were often significantly different between primary and metastatic samples and may constitute biomarkers to identify tumors at high risk of metastatic progression.

### Co-mutations in phenotypically normal cells

Recently, evidence of widespread somatic mutagenesis has been reported in phenotypically normal cells^2,3,52^. Hence, we wondered whether co-mutations observed in tumors were also present in normal tissues. To address this question, we analyzed somatic variants in normal urothelium^53^; 926 samples from 15 donors; skin^54,55^; 2,580 samples from 17 donors and esophageal epithelium^2,56^; and 918 samples from 12 donors. Given the low number of donors, but high number of samples per donor, we searched for co-mutations among samples, using donors as covariates, and reannotated somatic variants to retain only putative oncogenic mutations in our panel of 396 genes.

**Fig. 4:**
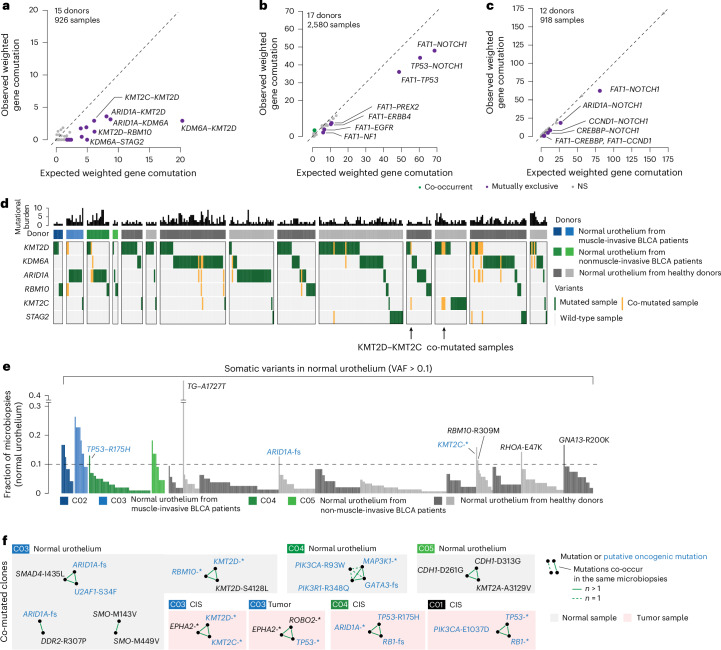
Co-mutations in normal tissue samples. **a**–**c**, Expected (*x* axis) versus observed (*y* axis) weighted co-mutation incidence among gene pairs in normal urothelium (**a**; *n* = 28), normal skin (**b**; *n* = 45) and normal esophageal epithelium (**c**; *n* = 66) samples. The most significant gene pairs are labeled. **d**, Detailed oncoprint of putative oncogenic mutations in normal urothelium samples (column) across six recurrently mutated cancer genes (rows). Mutated samples are in green if only one of the six genes is mutated, or yellow if several genes are mutated. Wild-type samples are in gray. Samples from the same donors are grouped and contoured in black. Donor types are color coded. Top: total mutation burden per sample (barplot). **e**, Barplot showing the fraction of samples of a given donor where each somatic variant (bars) was detected. Only variants exhibiting a VAF >0.1 in at least one sample were retained. Representative variants detected in more than 10% of the samples are labeled by the gene symbol where the mutation occurs followed by either the specific amino substitution for missense mutations, an asterisk for nonsense mutations or ‘fs’ for frame-shift mutations. Mutations are labeled in blue if classified as putative oncogenic, or in black otherwise. **f**, For normal urothelium samples derived from cancer patients (gray boxes), we report a selection of somatic variants detected in at least one biopsy with a VAF >0.1, and that co-occurred among each other across more than one biopsy (solid green edges). Only one variant (*PIK3CA–*R93W) was detected in a single biopsy (dashed green edge). Co-mutations from CIS and tumor samples are in red boxes.

Across all datasets, SelectSim detected significant mutually exclusive mutations, but no significant co-occurrences, except for a moderately significant pair that was detected in a single donor (Fig. 4a–c). These results were in stark contrast with significantly co-occurrent mutations detected in melanoma, bladder and esophageal cancers (Fig. 2e). Interestingly, even though normal urothelium samples exhibited somatic variants at commonly altered genes in bladder cancer, none of the significant co-occurrent patterns were found. Furthermore, some of most significant co-occurrent mutations in bladder cancer were significantly mutually exclusive in normal urothelium, such as mutations of *KDM6A* and *STAG2*, or *KMT2C* and *KMT2D* (Fig. 4a,d). These results indicated that, although oncogenic variants occur in normal tissues, their significant co-occurrence is primarily a cancer feature.

To further investigate co-mutations in normal tissues, we examined all unique somatic variants in cancer-associated genes, having a variant allele frequency (VAF) >10% in at least one normal urothelium sample. Among the 15 donors that we examined, 4 also exhibited carcinoma in situ (CIS) and/or invasive bladder cancer. In the normal tissue samples of three out of four cancer patients, more than one variant were found across >10% of the samples, indicative of the expansion of the mutated clone, whereas this was observed only in 1 out of 11 healthy donors (Fig. 4e). On average, normal tissue samples from each of the four cancer patients shared a higher fraction of mutations than samples from healthy donors (8% versus 4%), including putative oncogenic variants such as LoF mutation of *ARID1A* and a hotspot mutation of the splicing factor-encoding gene *U2AF1*, and LoF mutations at *KMT2D* and *RBM10* (Fig. 4f). Nevertheless, neither of these co-mutations were enriched in bladder cancer, or retained in matching CIS or tumor samples from these donors. Conversely, in matching tumor samples from three out five cancer patients, we found bladder cancer-associated co-mutations, such as co-occurrent mutations of *KMT2D* and *KMT2C* in C03, and of *RB1* and *TP53* in C01 and C04 (Fig. 4f). Although the relevance of these observations will require further data and experiments to be assessed, these results suggest that co-occurring oncogenic mutations are observed predominantly in tumors and may be required for malignant transformation.

## Discussion

Over the past decades, DNA mutations across the exome and genome of thousands of tumor samples have been identified, cataloged and classified based on their oncogenic capacity^19,29^. This represents an invaluable foundation for decoding the genetic bases of tumor evolution. However, cancer is not the result of individual mutations but of the concerted action of specific co-mutations, in favorable cellular contexts. As such, it is now critical to understand which combinations of genetic and nongenetic alterations contribute to tumor evolution.

Exploiting the surge of clinical sequencing data, we proposed an algorithmic framework to integrate numerous and heterogeneous tumor cohorts in an efficient and robust manner, overcoming the limitations of past approaches. Clinical sequencing datasets are increasing at a pace of >10,000 new samples annually. From a methodological perspective, with increasing data availability, a larger overlap of sequenced gene panels and standardized mutation calling procedures, we envision the possibility of robustly estimating the probability of mutating each gene in a given tumor independently of the specific dataset. This could provide analytical frameworks to learn the likelihood of co-mutations, infer driver co-mutations robustly, and ultimately chart and anticipate variant selection. In our study, co-mutations revealed distinct cancer evolutionary trajectories associated with patient prognosis, uncharacterized oncogenic variants, biomarkers of metastatic progression and malignant transformation. Improving oncogenic co-mutation catalogs will serve the rational design of (1) experimental models to better mimic the human disease and functionally characterize genetic variants in different contexts, (2) patient stratifications accounting for the composite risk of co-mutations and (3) combination therapies, exploiting synergies and vulnerabilities emerging from the copresence (or absence) of specific mutations.

**Extended Data Fig. 6:**
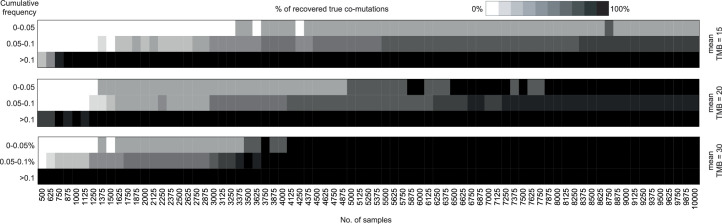
Benchmarking SelectSim on synthetic data. Fraction of true co-mutations that were recovered as a function of the number of samples in the synthetic dataset (x-axis) and mean tumor mutation burden (TMB) in the dataset (one heatmap for each mean TMB). Each row indicates the cumulative mutation frequency range of the gene pairs.

Co-mutation analyses are, however, still hampered by data sparsity and heterogeneity, which is a consequence of a high number of genes that are mutated with relatively low frequency. An increasing number of cancer-associated genes translates in the combinatorial explosion of possible co-mutations, challenging the ability to scale beyond gene pairs. Low mutation frequencies limits instead the statistical power to detect co-mutations. Using our model to generate synthetic datasets with predefined co-mutations, we found that, among gene pairs with a combined mutation frequency below 5%, co-mutations were identified only partially even with 10,000 samples (Extended Data Fig. 6), especially when the mutation burden of these samples was low. This is often the case for several cancer-associated genes and even more for individual variants. Indeed, different mutations in the same gene may induce different dependencies and, thus, co-mutations^57^. However, with the current datasets, co-mutations among unique variants would be detectable for only a handful of hotspot mutations at few oncogenes.

Somatic mutations are only one of several alteration modalities typically observed in tumor cells. Previous work has shown that copy number alterations and structural variants also exhibit significant co-occurrence and mutual exclusivity patterns^14,25^. Their integration is a much-needed next step, which will however require a proper estimation of their emergence rate as a function of their size and magnitude. Similarly, in future work, co-mutations should be studied in combination with the transcriptional and/or epigenetic states of the cells where they emerge^58^. Such multimodal analyses will be crucial to test the oncogenic competence of co-mutations in different contexts and identify cancer-promoting cell states, especially in the early phases of the disease. Indeed, somatic mutagenesis in normal tissues is prevalent and may set the seed of malignant transformation. Expanding the analysis of co-mutations in large cohorts of normal tissues with matching transcriptional and/or epigenetic profiles will be required to understand the genetic and cellular contexts that enable tumorigenesis.

Currently, precision oncology relies largely on genetic data, where discovering co-mutations may be critical to understanding and anticipating disease progression and therapeutic response. In the near future, we envision that similar approaches will leverage the convergence of multimodal data across several human tumors to discover, characterize and target oncogenic systems of co-alterations.

## Methods

### Statistics and reproducibility

No statistical method was used to predetermine sample sizes—they were determined based on dataset availability from public repositories (TCGA, GENIE). Exclusion criteria were the lack of clear tumor type annotation, patients with several samples and the number of samples of a particular tumor type smaller than 100 (GENIE). Randomization procedures are intrinsic to the algorithmic and statistical approaches adopted and presented below. This work does not include any new experiments that would require blinding. All data that were used are publicly available under the TCGA program and The American Association for Cancer Research (AACR) GENIE project.

### Human cancer datasets and annotations

#### TCGA dataset

The TCGA dataset comprises 9,082 patients with molecular and clinical annotations. We downloaded somatic point mutation data curated in the MC3 initiative^20^ and annotated the tumor samples.

#### The AACR GENIE dataset

The AACR Project, Genomics Evidence Neoplasia Information Exchange cohort (v15) (https://www.aacr.org/professionals/research/aacr-project-genie/aacr-project-genie-data/), aggregates molecular and clinical annotations from 19 cancer centers, encompassing data from 171,195 patients. For all GENIE tumor cohorts (v15), we generated genome alteration matrices (GAMs) retaining mutations classified as oncogenic or probably oncogenic in OncoKB and generating binary mutation calls for each gene and sample (Supplementary Data 1).

For co-mutation analyses, we used molecular data from the two largest cancer centers: MSK and DFCI. The MSK cohort comprises data from 63,596 patients, whereas the DFCI cohort includes 35,366 patients. Notably, several samples may be available for each patient. For our analysis, we retained samples categorized as primary or metastasis and eliminated patients with more than one sample. Next, we excluded samples lacking tumor type annotations (for example, ‘Carcinoma of unknown primary’ or ‘Unknown’). Finally, we retained only tumor types with at least 100 samples. Upon these filtering steps, we retained mutation data for 42,790 patients in the MSK cohort and 22,264 patients in the DFCI cohort. In the MSK cohort, 27,825 samples were classified as primary (MSK-P), and 14,965 as metastasis (MSK-M), whereas in the DFCI cohort, 14,657 were classified as primary (DFCI-P) and 7,607 as metastasis (DFCI-M).

Co-mutation analyses were performed on 74,136 samples from five cohorts: TCGA, MSK-P, MSK-M, DFCI-P and DFCI-M.

### Sample annotations

Tumor type and subtype annotations for the TCGA dataset were collected from Pan-Cancer Atlas publications^4^. Tumor samples from the AACR GENIE datasets were annotated using the corresponding OncoTree code provided in the original metadata (10.7303/syn53210170). In addition, tumor samples from colorectal cancer and endometrial cancer cohorts (OncoTree codes: COADREAD, COAD, READ, UCEC, UEC) were stratified further in high TMB (≥19 mutations) and low TMB (<19 mutations) consistent with their bimodal distribution of TMB and previous annotations^48^. This classification was added as a proxy of the missing microsatellite instability annotation for these datasets. In addition, for MSK and DFCI breast cancer samples lacking histological classification into invasive ductal or lobular carcinoma (IDC and ILC, respectively), we inferred ILC cases based on loss of E-cadherin (*CDH1*)—a hallmark of the disease^50^. Finally, we mapped tumor type and subtype annotations from the TCGA, MSK and DFCI cohorts manually to obtain 39 tumor classes and 119 tumor subtypes (Supplementary Table 4).

### Cancer gene selection and mutation filtering

In our analyses, we retain mutation data for a catalog of previously defined 396 cancer-associated genes^25^. To retain only putative oncogenic mutations for these genes, first, we discarded ‘synonymous’ mutations and classified the remaining as either ‘missense’ or ‘truncating.’ Missense mutations are nucleotide changes that lead to single amino acid substitutions, and truncating mutations encompass several types of putative LoF mutation: in-frame and out-of-frame insertions and deletions, nonsense and nonstop mutations, variants in splice site, splice region and translation start site. Since truncating mutations are generally considered LoF variants, we did not apply further filters on these variants and retained all truncating mutations. Among missense mutations, we only retained those classified as ‘oncogenic’ or ‘probably oncogenic’ in the OncoKB knowledge base^29^. The complete list of retained putative oncogenic mutations is provided in Supplementary Table 3.

### SelectSim algorithm

Here we describe the SelectSim algorithm in its fundamental steps: simulation of somatic mutations, estimating weighted co-mutation incidence and estimating the statistical significance of co-mutations.

### Step 1: Simulate oncogenic mutation calls under the assumption of independence among mutations

The goal of this step is to generate binary matrices under the assumption of independence among genes and samples while maintaining gene mutation frequencies and tumor mutational burdens. A GAM is defined from a set of genes G, with cardinality $\left|G\right|={n}_{g}$, and a set of tumors T, with $\left|T\right|={n}_{t}$, such that for a matrix entry at row *i* and column *j*, it holds that ${GAM}\left({i},{j}\right)=1$, if ${g}_{{i}}$ is mutated in the tumor *t*_*j*_ and ${GAM}\left({i},{j}\right)=0$ otherwise. We can approximate the probability of ${g}_{{i}}$ being mutated in the cohort T by its mutation frequency in T:

$$P\left({g}_{i}=1\right){\approx }f\left({g}_{i}\right)=\frac{1}{{n}_{t}}\mathop{\sum }\limits_{t\in T}{GAM}\left({g}_{i},t\right)$$

Similarly, the probability of a tumor ${t}_{{j}}$ to acquire a mutation is given by its mutation rate $\mu \left({t}_{{j}}\right)$, which can be approximated by its relative TMB:

$$\mu \left({t}_{{j}}\right)=\frac{{\rm{TMB}}({t}_{{j}})}{{\sum }_{t\in T}{\rm{TMB}}(t)}$$

Where the TMB of ${t}_{{j}}$ was defined as the total number of mutations across all sequenced genes in that sample, which is computed from the original mutation annotation file.

It follows that the expected number of mutations of ${g}_{{i}}$ in ${t}_{{j}}$ is given by:

$$E\left({g}_{{i}},{t}_{{j}}\right)={n}_{t}f\left({g}_{{i}}\right)\mu \left({t}_{{j}}\right)$$

By extension, for all genes and tumors:

$$E={{\bf{n}}}_{{\bf{t}}}{\bf{f}}({\bf{g}})\otimes {\rm{\mu }}={{\bf{n}}}_{{\bf{t}}}{\bf{f}}({\bf{g}})\times {\mu }^{T}$$

where ${{\bf{n}}}_{{\bf{t}}}{\bf{f}}({\bf{g}})$ is the [${n}_{g}$× 1] vector of gene mutation counts, **μ** is the [${n}_{t}$× 1] vector of sample relative TMBs and E is the [${n}_{g}$× ${n}_{t}$] matrix resulting from their outer product (⊗). Note that given the overall low gene mutation frequencies (GAMs are typically very sparse), most values in E will be between 0 and 1. In our analyses, only ~0.04% of the values were greater than 1, hence we set $\max \left(E\right)=1$.

Exploiting the sparsity of the data, we can use E to simulate a new set of binary mutation calls using a rejection-acceptance sampling (RAS) approach that assumes a Bernoulli distribution for each entry of the GAM with parameter p equal to the corresponding entry of E. Following this strategy, to simulate a value for each entry of the GAM, we draw a sample that is equal to 1 (mutated) with probability p or 0 (wild type) with probability 1 − *p*. This is implemented by uniformly drawing a number between 0 and 1, and if such number is greater or smaller than p, then the entry of simulated GAM S is set to 1 or 0, respectively. Formally,

$$\hat{S}=E-{\mathrm{unif}\left(0,1\right)}_{{n}_{g},{n}_{t}}$$

and

$$S=[\hat{S}\in {R}^{+}]$$

which is,

$$S({i},{j})=\left\{\begin{array}{l}\begin{array}{cc}1\,\mathrm{if}\,\hat{S}({i},{j}) & > \,0\end{array}\\ \begin{array}{cc}0 & \mathrm{otherwise}\end{array}\end{array}\right.$$

where ${\mathrm{unif}\left(\mathrm{0,1}\right)}_{{n}_{g},{n}_{t}}$ is a matrix of the same size as E whose elements are sampled uniformly within the [0,1] interval, ${R}^{+}$ indicates the set of strictly positive real numbers and [*i*] is the Iverson bracket that is equal to 1, if *i* is true and 0 otherwise. In addition, we modified this procedure to exactly preserve gene mutation frequencies, here referred to as ‘row-sum corrected’ RAS or rcRAS. To do so, we modified the expression above as follows:

$${S}^{\mathrm{rc}}(i,j)=\left\{\begin{array}{l}\begin{array}{cc}1\,\mathrm{if}\,\hat{S}(i,j) & > \,{m}_{i}\end{array}\\ \begin{array}{cc}0 & \mathrm{otherwise}\end{array}\end{array}\right.$$

where ${m}_{i}$ is the value of $\hat{S}(i,\cdot )$, such that exactly ${n}_{t}f\left({g}_{i}\right)$ values exist that are greater than ${m}_{i}$. Finally, out of *N* simulations, we remove from the analyses 10% of the simulated datasets where the sample mutation frequencies deviate most from the observed values (mean absolute difference of total number of mutations per sample). (Hereafter, we refer to the simulated matrix S as the matrix generated with row-sum correction, omitting the ‘rc’ suffix for simplicity.)

**Extended Data Fig. 7:**
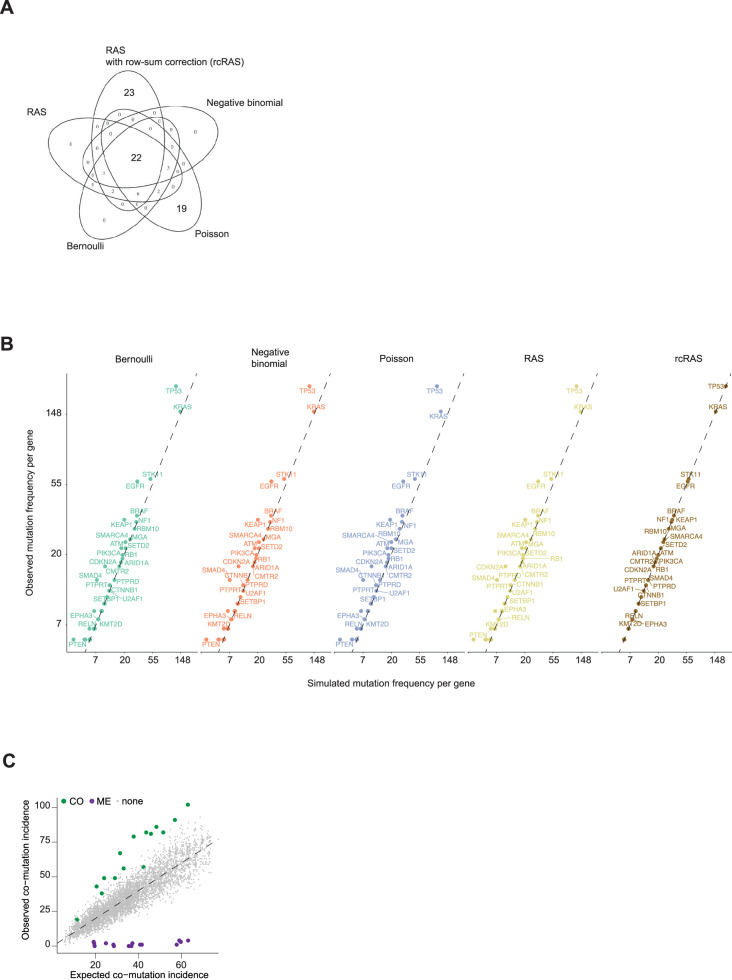
Comparison of sampling approaches to simulate gene alteration matrices. **A**) Intersection of significant co-mutations detected using 5 distinct sampling approaches to simulate gene alteration matrices. **B**) Simulated (x-axis) vs. observed (y-axis) gene mutation frequency based on 5 distinct sampling approaches. **C**) Co-mutation incidence observed in a model without evolutionary dependencies (x-axis, expected) and with 30 evolutionary dependent gene pairs (y-axis, observed), 15 synergistic pairs (green) and 15 antagonistic pairs (purple).

Notably, we benchmarked distinct sampling procedures by generating 1,000 GAMs using: (1) RAS, (2) rcRAS, (3) direct Bernoulli sampling, (4) negative binomial sampling and (5) Poisson sampling with subsequent binarization. Results obtained with the different sampling approaches exhibited a good overlap with 43% of significant co-mutations detected using more than one sampling approach and 30% (22 out of 74 in total) of them detected using all five approaches (Extended Data Fig. 7a). rcRAS recovered 23 additional co-mutations after applying row-sum correction, indeed a principal limitation of the other sampling approaches is that of inherently generating integers/binary values that do not exactly preserve gene alteration frequencies (Extended Data Fig. 7b). This was most evident for Poisson sampling, which most underestimate gene mutation frequencies, especially among recurrently mutated genes (*KRAS* and *TP53*), leading to a higher number of likely false positive hits (Extended Data Fig. 7a,b).

### Step 1b: Computing mutation expected counts accounting for variant and tumor (sub)types

To account for the presence of different tumor types and subtypes in the same dataset and different mutational processes, SelectSim generates and then integrates distinct *E* matrices.

Given an input a vector of tumor classes ${\bf{c}}=\{{c}_{1},\ldots ,{c}_{k}\}$, SelectSim separately computes $f\left(g\right)$ and μ values for each class, such that the final matrix of expected mutation counts concatenates the matrices derived for each class:

$$E=\left[{E}_{{c}_{1}},\ldots ,{E}_{{c}_{k}}\right]$$

Note that although SelectSim implementation currently supports only covariate vectors for tumor samples, the same strategy can also be implemented easily to account for gene covariates. In general, covariates that can be expressed as a partition of genes or samples, naturally translate into a partition of the E matrix in separate blocks, which can be computed and then recomposed separately.

To account instead for heterogeneous types of mutation, we need to put in place a different strategy, as this type of covariate does not partition genes or samples but instead defines different classes of the input mutation data. Taking the case of missense (${\rm{ms}}$) and truncating (${\rm{tr}}$) mutations, to account for the different rates of ${\rm{ms}}$ and ${\rm{tr}}$ mutations in genes and samples, SelectSim computes ${f}_{{\rm{ms}}}\left(g\right),{f}_{{\rm{tr}}}\left(g\right)$, ${\mu }_{{\rm{ms}}},{\mu }_{{\rm{tr}}}$ separately and, as a consequence: ${E}_{{\rm{ms}}}$ and ${E}_{{\rm{tr}}}$. The final matrix of expected mutation counts is defined as follows:

$$E=\max ({E}_{{\rm{ms}}},{E}_{{\rm{tr}}})$$

where max is the element-wise maximum function. (Note that, whereas the true expected number of mutations should be the sum of the two values, the max function was here preferred to aggregate the matrices given that we eventually use the E matrix to simulate binary mutation counts, that is a maximum of one mutation per sample is allowed, and, in any case, the thresholds used in the binarization strategy are selected to preserve the observed mutation counts.)

### Step 2: Computing the weighted co-mutation incidence

Given a GAM, the nominal incidence of co-mutation between each pair of genes is given by the matrix product:

$$\mathrm{CO}={GAM}{\,\times \,{GAM}}^{T}$$

In SelectSim, we introduced a sample weighting strategy to give higher weights to co-mutations occurring in samples with low TMB than in samples with high TMB. Precisely, we introduce a penalty vector **w** of length ${n}_{t}$ to penalize tumors in T as follows:

$${\bf{w}}=\frac{1}{1+{\max }\left[\lambda \left({{\bf{TMB}}}_{{\rm{FC}}}-\tau \right),0\right]}$$

where ${{\bf{TMB}}}_{{\bf{FC}}}$ is a vector of TMB fold changes (FCs) computed as the ratio between the TMB of each tumor and the median TMB of the cohort, and τ and λ are two parameters that define, respectively, the maximum ${{\bf{TMB}}}_{{\bf{FC}}}$ that is not penalized and the extent of penalization. In this study, we set *τ* = 1, to penalize TMBs that are higher than the median, and λ=0.3. Nevertheless, the performances of SelectSim were highly robust to parameter changes (Extended Data Fig. 1k). When a cohort comprises several tumor (sub)types, ${{\bf{TMB}}}_{{\bf{FC}}}$ is computed as the ratio between the TMB of each tumor and the mean of the median TMBs of each tumor (sub)type.

Once the penalty vector is computed, the weighted incidence of co-mutation between each pair of genes is given by:

$$\mathrm{wCO}=\left({\bf{w}}\times GAM\right){\times GAM}^{T}$$

where **w** × *GAM* is the column-wise element-wise products of $\bf{w}$ and ${GAM}$.

### Step 3: Estimating the statistical significance of co-mutations

To determine significant co-mutation patterns, we compare the observed weighted incidence of co-mutations in the real dataset ($\mathrm{wCO}$) with those expected by chance (${\mathrm{wCO}}_{\exp }$). The expected co-mutation incidence is estimated as the mean weighted co-mutation incidences obtained from *N* simulated mutation matrices $\{{S}_{1},\ldots ,{S}_{N}\}$ as defined in Steps 1 and 2:

$${\mathrm{wCO}}_{\exp }=\frac{1}{N}\mathop{\sum }\limits_{i=1}^{N}\left({\bf{w}}\times {S}_{i}\right)\times {S}_{i}^{T}$$

The weighted effect sizes associated with each pair of genes are then defined as follows:

$$\mathrm{wES}=\left(\mathrm{wCO}-{\mathrm{wCO}}_{\exp }\right)\sin \frac{\pi }{4}$$

which are the Euclidean distances between the point $\left(\mathrm{wCO}(i,j),{\mathrm{wCO}}_{\exp }(i,j)\right)$ and the diagonal $\mathrm{wCO}={\mathrm{wCO}}_{\exp }$. Note that these distances can assume a different range of values depending on the individual gene mutation frequencies as increasing mutation frequencies increases the probability of co-mutation and, thus, the possible maximal distance from the diagonal. Precisely:

$$\begin{array}{l}\min \left(\mathrm{wCO}\right)=0,\max \left(\mathrm{wCO}\right)={n}_{t}\left(\min \left(\,f\left({g}_{i}\right),f\left({g}_{j}\right)\right)\right)\\\to \max \left(\mathrm{wES}\right)=\max \left(\mathrm{wCO}\right)* \sin \displaystyle\frac{\pi }{4}\end{array}$$

To account for this dependency and make the magnitudes of $\mathrm{wES}$ values comparable across distinct gene pairs, we first compute the expected $\mathrm{wES}$ from each simulated matrix ${S}_{i}$ and define ${\mathrm{wES}}_{\exp }$ as the mean of such expected values; then, we define a normalized effect size as follows:

$$n\mathrm{ES}=\mathrm{sign}\left(\mathrm{wES}\right)* \max \left(\left|\mathrm{wES}\right|-\left|{\mathrm{wES}}_{\exp }\right|,0\right)$$

Note that if the expected weighted effect size is greater in magnitude than the observed weighted effect size, we set the normalized value to 0 to avoid changes of sign of the effect size when the observed values are negligible (nonsignificant).

The statistical significance of $n\mathrm{ES}$ values are then computed by empirically estimating a predefined threshold’s FDR^59,60^. Precisely, SelectSim estimates expected $n\mathrm{ES}$ values from each simulated matrix ${S}_{i}$ (as previously done for $w\mathrm{ES}$). Then, for a given threshold, *α*, the number of false positives (FP) is defined as the mean across all simulated matrices of the number of gene pairs with $n\mathrm{ES}$ > *α* in each simulated matrix. The number of true positives (TP) is the number of gene pairs with $n\mathrm{ES}$ > *α* in the real dataset. The FDR associated with *α* is then FP/(TP + FP). In this study, we selected *α* such that FDR < 0.1 or FDR < 0.25 to determine significant co-mutations, depending on the analysis (‘Results’). Given the vast number of performed statistical tests (which correspond to the number of gene pairs), the procedure for FDR risks overestimating the number of false discoveries, especially when $f\left(g\right)$ is highly variable. To counterbalance this risk and further alleviate the dependency between $n\mathrm{ES}$ and $f\left(g\right)$, we compute the FDR of $n\mathrm{ES}$ scores within categories of gene pairs that were determined by the cumulative mutation frequency of the genes $f\left({g}_{i}\right)+f\left({g}_{j}\right)$. In this work, we chose four categories defined by the following cumulative frequency intervals: (0,0.02), (0.02,0.05), (0.05,0.10) and (0.10,1). Nevertheless, performances were robust to different numbers of intervals (Extended Data Fig. 1l).

### Application of SelectSim on different datasets

#### The Cancer Genome Atlas

From the TCGA MC3 mutation annotation file, we generated separate missense and truncating mutation matrices (GAMs) for 9,082 samples for 396 cancer genes. TMBs were computed separately for missense and truncating alterations. Genes mutated in fewer than nine samples (frequency <0.1%) in the combined GAM of missense and truncating mutations were omitted (Supplementary Data 2).

#### MSK-P, MSK-M, DFCI-P and DFCI-M

Samples were sequenced within each of the four cohorts with different gene panels. We ran SelectSim independently for each gene panel for each cohort, subsetting the cohort to the samples sequenced by the panel in question. We defined the ‘all’ panel consisting of the genes common among all the panels, where all samples from a given tumor-specific class or pancancer class were analyzed together (Supplementary Table 2). In the analyses, TMBs were estimated from the panel of sequenced genes, only tumor types with >30 samples, and only genes that were mutated in at least 0.1% of cases (missense + truncating mutations) were retained (Supplementary Data 2).

### Generation of a compendium of co-mutation patterns in primary cancers

To consolidate significant co-occurrent and mutually exclusive gene mutations and develop a comprehensive catalog of independently validated hits, we retained gene pairs that were found significant (eFDR < 0.25) with the same pattern (co-occurrent or mutually exclusive) in at least two primary tumor cohorts, among TCGA, MSK-P and DFCI-P (hereafter referred to as ‘consistent hits’). Given several gene panels in the MSK-P and DFCI-P cohorts, we ran SelectSim on each sample set that was analyzed by the same gene panel (panel-specific analyses) and on all samples testing the intersection of the gene panels (all-panel analysis). Gene pairs that were testable and significant in the all-panel analysis were retained, gene pairs that were significant only in panel-specific analyses were retained only if they were not testable in the all-panel analysis. Results from the all-panel analysis were given higher priority as these were derived from the largest sample size.

Pancancer consistent hits were found significant in at least two separate pancancer analyses. For tumor class-specific analyses, we analyzed harmonized tumor classes (Supplementary Table 6) and retained 19 tumor classes that were present in all three cohorts. Pancancer and tumor class-specific consistent hits are reported in Supplementary Table 7. Note that although skin cutaneous melanoma and uveal melanoma were initially considered as subgroups (covariates) of a unique tumor class (‘melanoma’), given these are largely distinct tumor types, results in Fig. 2 are presented separately for the two cohorts.

### Co-mutation analysis for primary versus metastatic samples

To compare co-mutations in primary versus metastatic samples, we selected the MSK cohorts analyzed by the largest gene panels—MSK 505 and MSK 468—which have 306 genes in common. To identify co-mutations that were enriched in specific populations, we compared MSK-P and MSK-M samples as follows:

Step 1: we first filtered MSK-P and MSK-M to common tumor types and ensured each had more than 30 samples. We then selected a number of samples for each tumor type corresponding to 80% of minimum number of samples between the MSK-M and MSK-P cohorts for that tumor type. This led to a total of 8,196 samples across 50 tumor types. In this way, we generated ten subsampled cohorts for P and M samples, ensuring that the tumor type composition was identical in both datasets. SelectSim was then run on each subsampled cohort (ten for P and ten for M). Step 2: as a null model, we generated 20 subsampled cohorts of the same size (*n* = 8,196 samples), admixing 50% of P samples and 50% of M samples. Ten subsampled cohorts were labeled as ‘primary’ and the other ten as ‘metastasis.’ SelectSim was then run on each subsampled cohort. Step 3: the union of significant hits from primary and metastatic cohorts were extracted, and we computed the mean weighted co-mutation effect size ($\mathrm{wES}$) of these gene pairs in the ten P runs (${\mathrm{wES}}_{{\rm{P}}}$) and in the ten M runs (${\mathrm{wES}}_{{\rm{M}}}$). The same procedure was followed for the randomized P and M cohorts. Step 4: observed ${{\Delta}}{\mathrm{wES}}$ values were computed as the differences ${\mathrm{wES}}_{{\rm{M}}}-{\mathrm{wES}}_{{\rm{P}}}$ for each gene pair. Expected ${{\Delta}}{\mathrm{wES}}$ values were computed as the differences obtained for the randomized P and M cohorts. Step 5: P- and M-enriched gene pairs were determined by comparing observed and expected $\left|{{\Delta}}{\mathrm{wES}}\right|$ values, as those with $\left|{{\Delta}}{\mathrm{wES}}\right|$ values exceeding the 99th percentile of the expected distribution. Step 6: finally, we classified a gene pair as M-specific (P-specific) if it was found significant in at least six M (P) subsampled cohorts and in fewer than two P (M) subsampled cohorts.

To compare individual gene frequencies, we compared the alteration frequencies for each gene in the ten subsampled M cohorts versus the ten subsampled P cohorts. Alteration frequencies were compared with a two-tailed *t*-test, and genes obtaining adjusted *P* values <0.05 were considered significant.

### ElasticNet analysis for primary versus metastatic samples

We ran the elastic net regression analysis using the MSK primary cohort, where curated clinical annotations and metastatic spread information were available through the MSK-MET cohort^48^ (12,925 samples). As explanatory variables, we included the tumor type of the samples (*n* = 27), the mutational status of 396 cancer-associated genes, 136 co-mutations classified as P- or M-enriched and 43 drug treatments based on the annotations that were available through MSK-CHORD project. The variables were retained if the (co)mutational event occurred in at least five samples. We designed two elastic net regression models (‘glmnet’ R package), logistic and Gaussian, to explain two dependent variables, the binary presence of metastasis (number of metastases >0) and the number of metastases in a sample, respectively. We repeated the regression ten times to ensure more stable results, as the cross-validation steps for determining the optimal penalization parameters introduce some randomness. We calculate the mean coefficient across the ten regressions for each variable and record the number of times it was selected (that is, the corresponding coefficient is nonzero). We then filter the variables, retaining only those selected ten times. Heatmaps in Fig. 4f report the row-normalized values (scaled with the sum of absolute values in a row).

### Co-mutation analysis for normal tissue samples

Somatic mutations in phenotypically normal tissues were retrieved from five different studies examining normal urothelium^53^ (for which we retained 926 samples and 15 donors), skin^54,55^ (2,580 samples and 17 donors) and esophageal epithelium^2,56^ (918 samples and 12 donors). For all studies, we retrieved annotated variant calls resulting from targeted sequencing and provided in their respective publications, except for the study by Lawson and colleagues^52^, which provides only variant calls that we annotated using the online variant effect predictor tool (https://grch37.ensembl.org/Homo_sapiens/Tools/VEP). We used SelectSim to test co-mutation patterns among individual samples, using donors as covariates, and analyzed each tissue type separately, integrating data from studies analyzing the same tissue. While constructing the GAMs, we retained only putative oncogenic mutations within our panel of 396 genes. We retain genes (rows) in the GAM only if they are mutated in at least two samples. Samples from ref. ^52^ were retained only if classified as ‘normal urothelium.’

To analyze mutations shared across several normal urothelium microbiopsies (Fig. 4e,f), we interrogated unique variants that were detected with a VAF >0.1 in at least one microbiopsy and, for each variant, we computed the fraction of microbiopsies from a given donor in which that variant was detected. All variants detected in at least 10% of the microbiopsies of a given donor were detected in at least three microbiopsies.

### Reporting summary

Further information on research design is available in the Nature Portfolio Reporting Summary linked to this article.

## Online content

Any methods, additional references, Nature Portfolio reporting summaries, source data, extended data, supplementary information, acknowledgements, peer review information; details of author contributions and competing interests; and statements of data and code availability are available at 10.1038/s41588-026-02661-4.

## Supplementary information

Supplementary Information Supplementary Note, Figs. 1–5 and Table 1.

Reporting Summary

Peer Review File

Supplementary Table Table 1. Methods and features overview. Table 2. Description of the GENIE cohorts. Table 3. Catalog of putative oncogenic mutations across 396 cancer-associated genes. Table 4. Sample annotations for TCGA, MSK and DFCI cohorts. Table 5. SelectSim complete set of results for all pancancer and tumor-specific runs. Table 6. List of primary samples used in the consistent co-mutation analysis (Fig. 2). Table 7. SelectSim complete set of results for the consistent co-mutation analysis (part 1). Set of consistent co-mutations (part 2). Table 8. Primary versus metastasis enrichment of gene mutations and co-mutations. Table 9. ElasticNet predictor matrix (part 1) and models’ coefficients (part 2).

## Data Availability

We retrieved publicly available mutational data for the TCGA dataset from https://gdc.cancer.gov/about-data/publications/Pathways-2018 and for the AACR Project Genomics Evidence Neoplasia Information Exchange cohort (version 15) from 10.7303/syn53210170. Data for the MSK-CHORD dataset was retrieved from https://www.cbioportal.org/study/summary?id=msk_chord_2024. Additional datasets were collected from the supplementary material of the corresponding publications. Supplementary Data 1 (GAMs of the GENIE cohorts) and Supplementary Data 2 (preprocessed input for SelectSim of all used cohorts) are available via Zenodo at 10.5281/zenodo.13752869 (ref. ^61^) Additional data including input and results of the analyses in this manuscript are available via Zenodo at 10.5281/zenodo.13769785 (ref. ^62^).

## Code availability

SelectSim is implemented as an R Package and is available via GitHub at https://github.com/CSOgroup/SelectSim (ref. ^60^) and via Zenodo at 10.5281/zenodo.19680236 (ref. ^59^). Additional scripts to reproduce the primary analyses presented in this manuscript are available at https://github.com/CSOgroup/SelectSim_analysis.
