# Supplementary material for: Evolving patterns of co-mutations from tumor initiation to metastatic progression
Source: Nat Genet. 2026 Jul 10;58(8):2053–62. doi: 10.1038/s41588-026-02661-4 (PMC13447111; doi:10.1038/s41588-026-02661-4)
Supplement: Supplementary file 1 [file 41588_2026_2661_MOESM1_ESM.pdf]

nature genetics

Supplementary information https://doi.org/10.1038/s41588-026-02661-4

Evolving patterns of co-mutations from tumor initiation to metastatic progression

In the format provided by the authors and unedited

Supplementary Note

1) Analysis of tumor purity and variant allele frequency

Low tumor purity and variant allele frequencies can affect mutation calling and discovery of co-mutation patterns, subsequently biasing any downstream analysis. To verify that the results of our study are not systematically affected by these confounders, we retrieved tumor purity estimates and variant allele frequencies (VAF) when available in our cohorts and tested whether these values were associated with mutation and co-mutation incidences, and, thus, with SelectSim scores.

First of all, tumor purity may affect our results by "hiding" true cancer mutations, i.e., mutations may be missed in samples with low purity, especially if sub-clonal. While this may certainly be true in specific cases, to affect our results this should be true systematically and result in a positive correlation between the number of mutations detected in a sample and its purity. We tested this hypothesis in both TCGA and MSK cohorts, but in both cases no correlation was found (Spearman's $cor_{TCGA} = -0.042$, $cor_{MSK} = 0.0071$, **Fig. SN1A**).

Next, we specifically tested potential biases introduced by variable VAF for the mutations that we analyzed. Indeed, VAF can vary because of both tumor purity and tumor clonality. For each significantly co-occurrent (CO) or mutually exclusive (ME) gene pair, we examined VAF values in samples exhibiting a mutation in only one of the two genes (single-mutant) or in both genes (double-mutant). VAF differences between these two groups would invalidate our results in the following cases:

- <u>Significant lower VAF in double-mutant samples vs. single-mutant samples</u> may suggest that although double-mutant samples harbor mutations at both genes, these may be sub-clonal and possibly not occurring in the same cells. If so, such evidence may invalidate significant co-occurrence patterns. (Significant mutual exclusivity would not be invalidated, as such a scenario would make the mutual exclusivity even stronger.)

- <u>Significant higher VAF in double-mutant samples vs. single-mutant samples</u> may suggest that mutation incidence in single-mutant samples is underestimated and therefore additional variants may be present that were missed. If so, such evidence may invalidate significant mutual exclusivity patterns. (Significant co-occurrence would not be invalidated, as such a scenario would make the co-occurrence even stronger.)

To perform this analysis, we analyzed all CO and ME gene pairs comprising at least 10 double-mutant and 10 single-mutant samples and that were found significant in the pan-cancer TCGA dataset. Expected VAF differences between single- and double-mutated groups (i.e., the null distribution) were estimated by permuting single- and double-mutant sample labels. Upon comparing VAF values between single-mutant and double-mutant samples, we found no evidence of systematic bias (**Fig. SN1B**): only 3 hits out of 232 barely exceeded the 95% confidence interval of the null distribution and entered into the "problematic conditions" that we described above (**Fig. SN1B**). Nevertheless, we examined these 3 cases in more detail: in all 3 cases, the difference in VAF was modest and the mean VAF values were always above 0.25, unlikely to correspond to true sub-clonal variants (**Fig. SN1C**). (In our opinion, the only dubious case may be the co-occurrence of *CDKN2A* and *KRAS* mutations, which in double mutant samples have a mean VAF close to 0.25, yet these mutations are among the most recurrent in pancreatic and lung cancer and have been already reported as co-occurrent[1,2].

Overall, while we cannot exclude that in some samples a few variants may have been missed or co-occur in different clones, *these effects are not systematically observed and are unlikely to invalidate our results*.

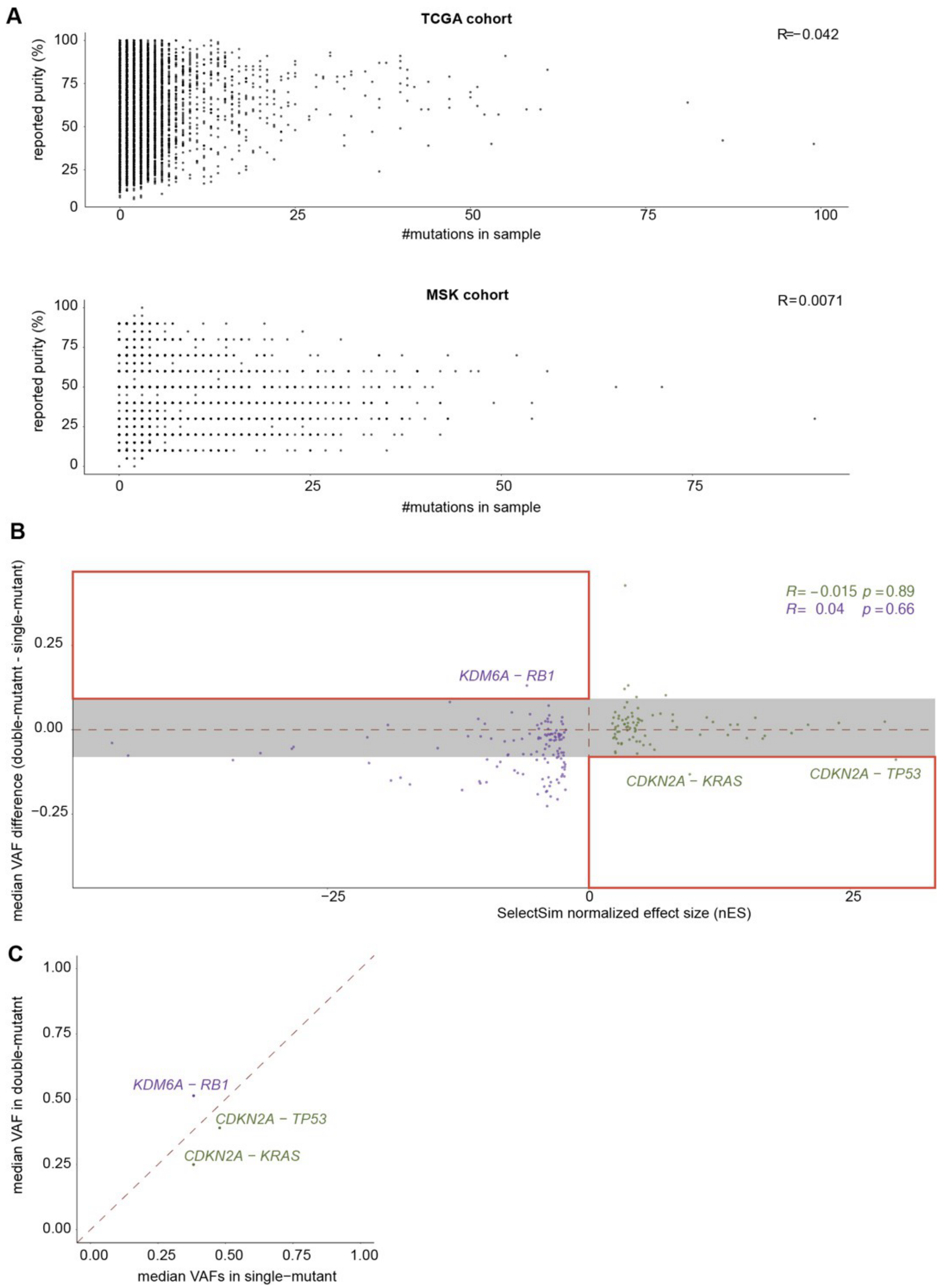

Figure SN1: **A)** Number of mutations per sample (X-axis) vs. estimated tumor purity per sample (Y-axis) in the TCGA (top) and MSK (bottom) cohorts. **B)** SelectSim normalized effect size (nES, X-axis) vs. difference of the median variant allele frequencies (VAF) between double- and single-mutant samples (Y-axis) for significant co-mutations in the TCGA cohort. **C)** Median VAF for selected co-mutation in single- (X-axis) vs double-mutant (Y-axis) TCGA samples.

2) Effects of neutral/synonymous mutations on SelectSim

In our analyses, we compared how applying SelectSim on a GAM based on driver genes with putative oncogenic mutations compares to applying SelectSim on a GAM based on synonymous mutations (**Figure 1C**). However, synonymous and non-synonymous mutations may not occur at the same rate, hence altering individual gene mutation frequencies. In our dataset, synonymous and non-synonymous mutation frequencies for each gene were correlated, but exhibited also substantial deviations (**Figure SN2A**). In particular, a small set of frequently mutated cancer genes had significantly more oncogenic mutations than synonymous ones (**Figure SN2A**).

To assess the effect of introducing neutral mutations while preserving gene mutation frequencies, we built multiple datasets where for each gene we introduced a progressively increasing percentage of neutral mutations, decreasing by the same amount the number of oncogenic mutations, thus, to preserve the total number of variants per gene. In practice, at first, we ran SelectSim retaining for each gene only oncogenic mutations. Next, for each gene, we substituted K% of oncogenic mutations with the same number of synonymous variants or missense variants classified as neutral (for this analysis, we excluded all truncating mutations) and repeated the SelectSim analysis. We repeated this analysis for K = 10, 20, 30, 50, 70, 80, 90. Results are shown in **Suppl. Figure 2A**. Note that, even in this setting, it was not possible to preserve the mutation frequencies of a small subset of oncogenes and tumor suppressors for all values of K. In particular, 9 genes exhibited a high frequency of oncogenic mutations (HF genes) and not enough synonymous or putative neutral missense mutations for large values of K (**Figure SN2B**). Nevertheless, we found that introducing 20% neutral variants allowed to largely preserve alteration frequencies for all genes and was already sufficient to reduce the number of significant co-mutations by 40% (**Figure SN2C**). Importantly, even co-mutation patterns that involved only one or none of the HF genes reduced and eventually disappeared with increasing percentages of neutral variants (**Figure SN2C**). In addition, we repeated the analysis for “randomly” sampled genes using again only missense variants and selecting a subset of 172 genes with matching mutation frequencies (±5%). Also in this case, several significant co-mutations were detected for cancer-associated genes but not for the randomly sampled ones (**Figure 1E**).

Overall, while perfectly matching gene alteration frequencies between oncogenic and neutral mutation is not always possible, we show that already relatively small percentages of neutral variants are sufficient to reduce co-mutation patterns as nicely captured by SelectSim, further highlighting the ability of our method to detect co-mutations emerging as a result of evolutionary dependencies.

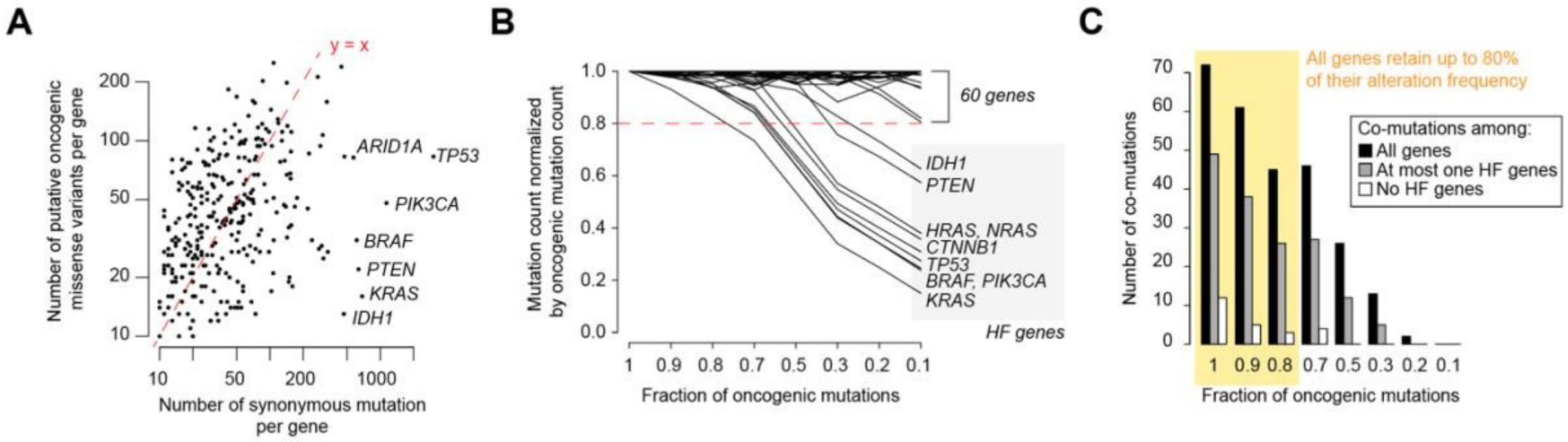

Figure SN2: **A)** Number of synonymous vs putative oncogenic mutations per gene in the TCGA cohort for the subset of cancer associated genes used in this study. Data reflect the analysis shown in Figure 1D. **B)** Total number of mutation per gene (y-axis) across datasets that were built by mixing different K% of putative oncogenic mutations (x-axis) and (100-K)% of synonymous or putative neutral missense mutations, when available, or maximum possible number, when not enough were available. For 9 genes (labeled) not enough synonymous or putative neutral missense mutations were found to maintain the total number of mutations equal to at least 80% of the total number of oncogenic mutations (here used as a reference to normalize the total mutation count on the y-axis). **C)** Number of significant co-mutations detected by SelectSim in the different datasets among all genes (black bars), among at most one HF gene (i.e., co-mutations exclusively involving HF genes were excluded - gray bars), and among non-HF genes (white bars). The yellow box comprises the datasets where all genes had a total mutation count greater than 80% of the total oncogenic mutation count.

3) Enrichment of co-mutations in samples with double-hit or single-hit mutations

Cancer-associated genes and, in particular, tumor suppressor genes often require a so-called "double-hit" to alter cell phenotypes, i.e. two independent alterations that inactivate both alleles. Given that in SelectSim we do not discriminate between single-hit and double-hit alterations, here we examined whether and how this omission affects our results.

We focus our analysis on significant co-occurrent mutations in the lung adenocarcinoma and pan cancer datasets from TCGA, selecting those that involved at least one tumor suppressor. Within these patterns, we computed the fraction of samples that exhibited 2 hits for the tumor suppressor gene(s), either among all samples where at least one of the 2 genes were mutated (sample union), or only among the samples where both genes were co-mutated (sample intersection). The rationale was to assess whether the "double-vs-single hit fraction" among samples harboring mutations in both genes was higher or lower than among samples harboring mutations in at least one gene. For this analysis, we classified a sample as harboring a "double-hit" alteration for a given gene, if that sample harbored either more than one mutation, or one mutation and one copy number loss (-1, -2 GISTIC calls) on that gene. Otherwise, a sample is said to harbor a "single-hit" alteration for that gene. Overall, this comparison showed a high correlation between double-vs-single hit fractions among all mutated samples and among co-mutated samples, suggesting that co-mutation patterns are not biased for either single or double hit samples (**Figure SN3**). Notably, the only points that deviated from the diagonal were co-mutations involving tumor suppressors mutated at high frequency, especially TP53 (**Figure SN3**). However, these deviations can be explained by the fact that the mutation frequency of TP53 is up-to-2 orders of magnitude higher than those of most other genes, and as such the double-to-single hit sample fractions for samples with at least one mutation is dominated by TP53 mutant samples and remain fairly constant; conversely the double-to single hit sample ratio of co-mutated samples exhibit more variability consistent with more variable co-mutation frequencies. Overall, these results indicate that co-mutations are not enriched in double or single hit samples and discriminating

among double and single hits is unlikely to increase the signal-to-noise ratio, but it would increase data sparsity and reduce statistical power.

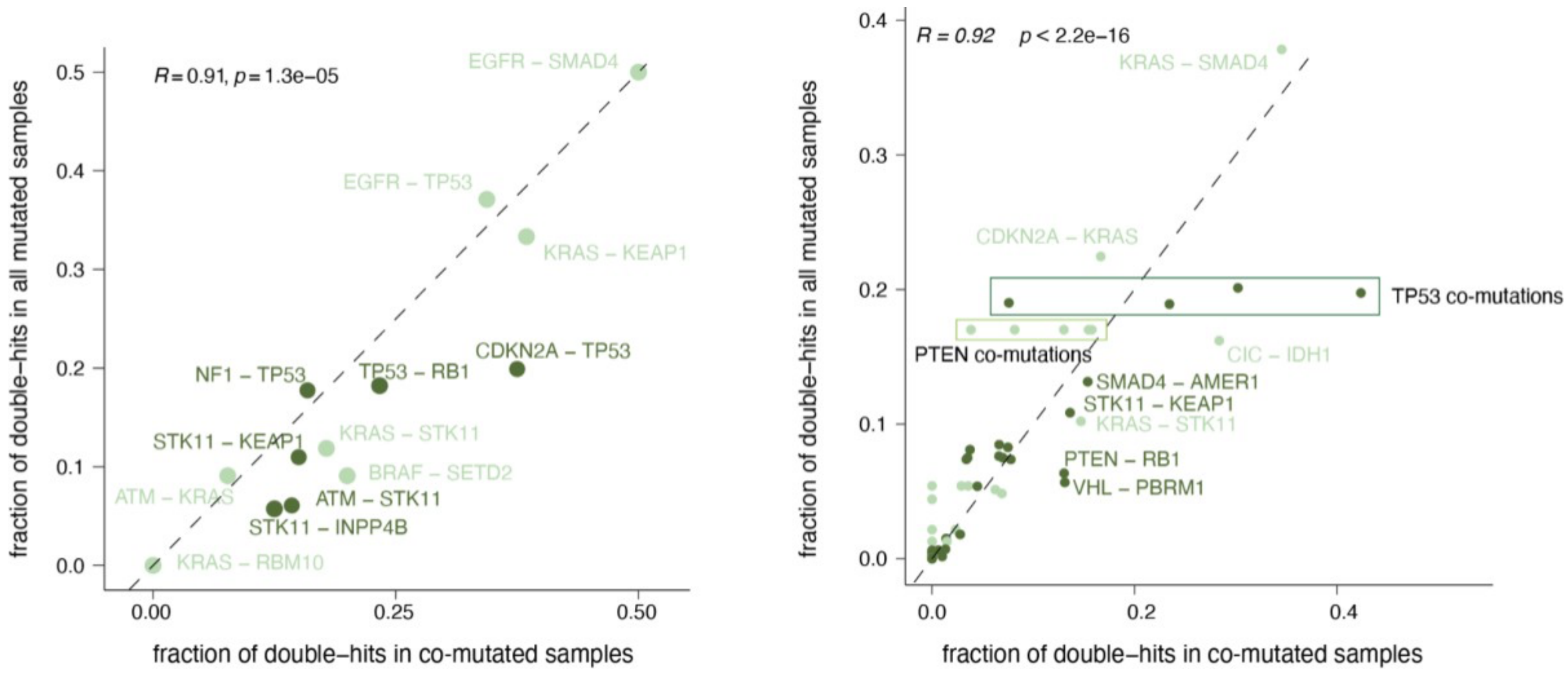

Figure SN3: Fraction of samples exhibiting a double hit event for tumor suppressors among co-mutated samples (x-axis) and among all mutated samples (y-axis). Each dot is a significant co-occurrently mutated pair of genes in the TCGA lung adenocarcinoma dataset (left) or in the TCGA pan-cancer dataset (right). Spearman's correlation coefficient and associated p-value are reported. Dark green dots refer to co-mutations between 2 tumor suppressors, and light green to co-mutations between 1 tumor suppressor and another gene.

4) Alternative models to account for treatment covariates in ElasticNet

In our study, we evaluated co-mutations as potential biomarkers of metastasis (**Figure 3**). The ElasticNet model included, besides co-mutations, several types of covariate predictors: single gene mutations, tumor types, and drug treatments. Drug treatments in particular could significantly impact the risk of metastatic progression, however these annotations are incomplete in our datasets and can be aggregated using different strategies. Here, we elaborate in more detail on these analyses.

At first, we considered treatment as a categorical variable assuming as values the reported drug[3] (**Table SN1**). Upon comparing the results of our model including or excluding drug treatments, we found that the ElasticNet coefficients for co-mutations were largely coincident (**Figure SN4A**). This result was consistent with the range of values of the ElasticNet coefficients obtained by treatment variables, which was was narrower than the one exhibited by co-mutation coefficients, indicating lower predictive values (**Figure SN4B**).

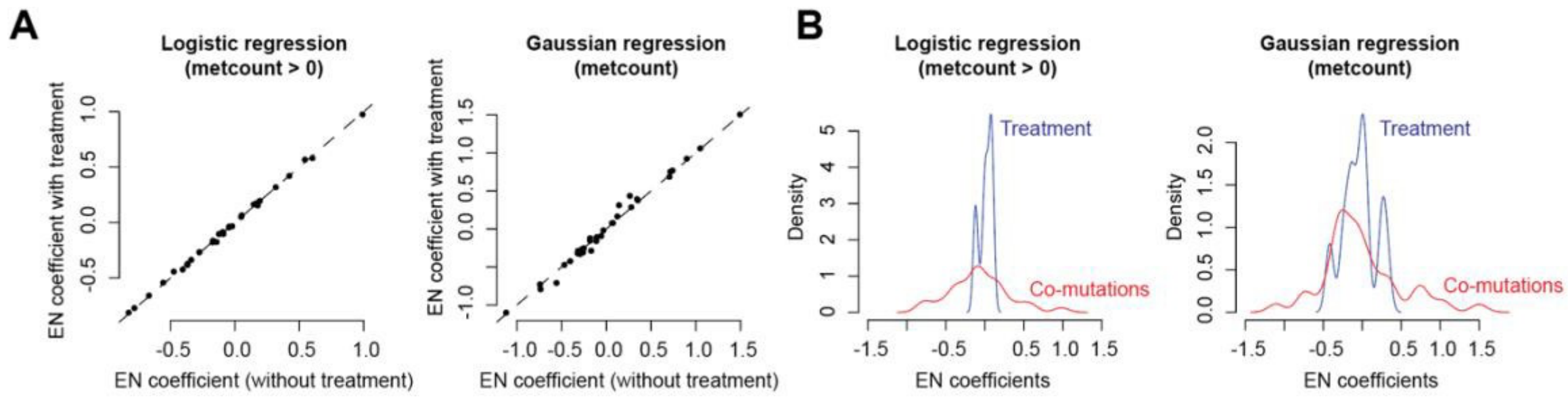

Figure SN4: **A)** Scatterplots of EN coefficient obtained by co-mutations when excluding (x-axis) or including (y-axis) treatment as a covariate. Results are shown for both logistic (left) and gaussian (right) regression models. **B)** Density distributions of EN coefficient obtained by co-mutations (red) or treatment (blue) for logistic (left) and gaussian (right) regression models.

Next, we wished to explore whether encoding the treatment predictors into specific drugs could have been inducing low coefficients only due to the small number of samples associated with each drug. As an alternative, we grouped predictors into broader treatment categories. We repeated the ElasticNet analysis grouping the treatment variables either based on their common joint administration (drug combinations), their targets (PD1, ER, AR, EGFR, PGR, VEGF, HER2, MTOR, CDK4/6, general chemotherapy), or based on their broad categorization (immunotherapy, hormonal therapy, targeted therapy, chemotherapy) (**Table SN1**). The stratification by target highlighted potentially interesting associations, such as lower metastatic risk for anti-PD1 -based treatment and increased metastatic risk for anti-VEGF (**Fig. SN5A**), whereas most of the other variables exhibited modest effect sizes (ElasticNet coefficients). In these analyses, we included age and smoking status as covariates, but neither of those exhibited strong predictive power (**Fig. SN5B**). Lastly, we examined the co-mutations that were found as predictive in these different ElasticNet runs. Strikingly, the results were highly consistent, with 34 out of 34 (100%) co-mutations found predictive of metastatic progression in all analyses, and 36 out of 48 co-mutations found predictive of the number of metastases in all analyses (**Fig. SN5C** and **Supplementary Fig. 5D**). Overall, while treatment annotations might be incomplete, we robustly associated co-mutations with metastatic risk independently of the type of treatment received by the patient.

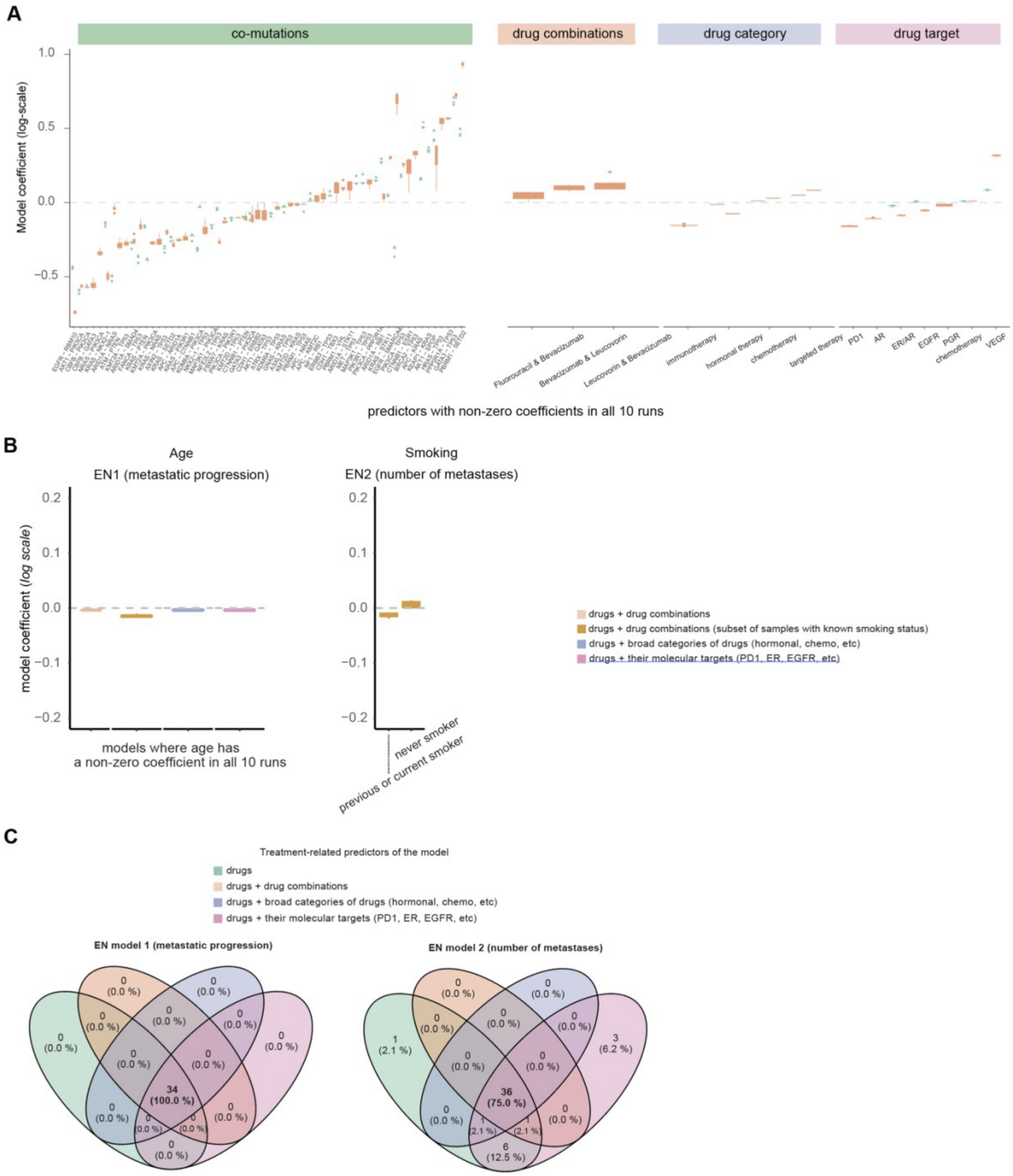

Figure SN5: **A)** ElasticNet coefficients of grouped variables: co-mutations, drug combinations, broad classes of drugs, and drugs grouped based on their molecular target. **B)** ElasticNet coefficients of age and smoking status in different models. Only variables with non-zero coefficients across all 10 repetitions of model training are plotted in **A)** and **B)** panels. **C)** Venn diagrams of co-mutations with strong predictive power across models that include different groups of treatments as variables.

DRUG NAME	TREATMENT TYPE	DRUG TARGET
ABIRATERONE	hormone	ar
BICALUTAMIDE	hormone	ar
ENZALUTAMIDE	hormone	ar
DEGARELIX	hormone	ar
ZOLEDRONIC ACID	targeted	bone
ABEMACICLIB	targeted	cdk4/6
PALBOCICLIB	targeted	cdk4/6
CAPECITABINE	chemo	chemo
CARBOPLATIN	chemo	chemo
CISPLATIN	chemo	chemo
CYCLOPHOSPHAMIDE	chemo	chemo
DOCETAXEL	chemo	chemo
DOXORUBICIN	chemo	chemo
ERIBULIN	chemo	chemo
ETOPOSIDE	chemo	chemo
FLOXURIDINE	chemo	chemo
FLUOROURACIL	chemo	chemo
GEMCITABINE	chemo	chemo
IRINOTECAN	chemo	chemo
LEUCOVORIN	chemo	chemo
METHOTREXATE	chemo	chemo
OXIPLATIN	chemo	chemo
PACLITAXEL	chemo	chemo
PEMETREXED	chemo	chemo
VINORELBINE	chemo	chemo
PACLITAXEL PROTEIN BOUND	chemo	chemo
ERLOTINIB	targeted	egfr
OSIMERTINIB	targeted	egfr
PANITUMUMAB	targeted	egfr
ANASTROZOLE	hormone	er
EXEMESTANE	hormone	er
FULVESTRANT	hormone	er
LETROZOLE	hormone	er
TAMOXIFEN	hormone	er
LEUPROLIDE	hormone	er/ar
PERTUZUMAB	targeted	her2
TRASTUZUMAB	targeted	her2
EVEROLIMUS	targeted	mtor
NIVOLUMAB	immuno	pd1
PEMBROLIZUMAB	immuno	pd1
MEGESTROL	hormone	pgr
BEVACIZUMAB	targeted	vegf

Table SN1: Treatment annotations used in the ElasticNet analysis

5) Comparison of SelectSim performances with other methods

Unless stated otherwise, all comparisons were conducted using SelectSim and methods to identify co-occurrence and mutual exclusivity without requiring additional prior knowledge (de-novo). In total, beyond SelectSim, 6 methods were selected that met these criteria and previously demonstrated good performances[4]: SELECT[5], DISCOVER[6], weSME/CO[7], cooccur[8], Coselens[9], and GAMToC[10] (**Supplementary Table 1**). In addition, we assessed precision and recall of a standard two-way Fisher's test, which is currently adopted to estimate co-mutation in the cBioPortal.

<u>Robustness Analyses.</u> We tested all methods both at pan-cancer and individual tumor levels, with lung adenocarcinoma (LUAD) chosen as the representative tumor type. However, only SelectSim, SELECT, and DISCOVER allow users to input sample covariates; hence, in the pan-cancer settings, results from the other tools should be ignored as inflated by false positive hits driven by the associations between gene mutations and tumor lineages. To assess the robustness and accuracy of the methods, we evaluated two metrics: 1) A recovery score, which quantifies their sensitivity in recovering significant hits in sub-sampled cohorts, and 2) Mathew Correlation Coefficient (MCC), which accounts at once for false positives and negatives, and true positives and negatives[5]. We first executed all methods on the TCGA pan-cancer and LUAD datasets, comprising 9082 and 502 samples, respectively. Subsequently, we subsampled 5 times the pan-cancer cohort and 10 times the LUAD cohort, retaining 80% of the total samples at each iteration. The methods were run on the sub-sampled cohorts, and significant hits detected in each sub-sampled cohort by each method were compared with their corresponding ground truths. The recovery score is the percentage of true significant hits recovered in at least *n* sub-sampled cohorts, with n = 1, … , 5 for the pan-cancer cohort and n = 1, … , 10 for the LUAD cohort. The MCC metric was instead computed for each sub-sampled cohort, and value distributions were compared across different methods.

<u>Dependency on the Tumor Mutation Burden (TMB).</u> To test whether significant co-occurrent mutations were not due to co-mutations occurring in samples with high TMB, we compared the log normalized TMB of co-mutated samples vs. wild-type samples for significantly co-occurrent mutations with at least 3 co-mutated samples, which were detected in the TCGA LUAD cohort and the TCGA pan-cancer cohort. TMB values were compared using the Cohen's D effect size. In the pan-cancer cohort, comparison included only tools accounting for sample covariates.

<u>Generation of artificial dataset with true co-mutations</u>. To create a set of "true" co-mutations that can be used to benchmark co-mutation discovery tools, we develop a model that generates mutations under the hypothesis of conditional selection of variants. The model requires to predefine: the total number of genes n and a range of gene mutation frequencies $\boldsymbol{f} = [f_{min}, f_{max}]$, the total number of samples m and a range of tumor mutation burdens $\boldsymbol{t} = [t_{min}, t_{max}]$, and a set of interdependent gene pairs $dG = \{ (g_1, g_2), \dots, (g_r, g_s)\}$, such that if $(g_i, g_j) \in dG$, then mutating g_i (or g_j) will either promote (*synergistic interaction*, $sign(g_i, g_j) = 1$) or inhibit (*antagonistic interaction*, $sign(g_i, g_j) = -1$) mutations of g_j (resp. g_i). dG gene pairs are uniformly sampled from the gene set with the constraint that each gene can be involved in at most one interdependent gene pair. (While this constraint does not reflect observations in real datasets, where a gene can be involved in multiple co-mutations, it simplified the generation of artificial datasets, by avoiding the risk of generating inconsistent interactions, and it did not affect any downstream analysis.) Next, n gene mutation frequencies and m tumor mutation burdens are sampled within the predefined ranges of values and assigned to each gene and sample.

To generate one sample, the model iteratively assigns one mutation to one of the genes with probabilities proportional to their mutation frequencies. At each iteration, once a mutation is assigned to a gene g_i, the probability of mutating that gene is set to 0 and if g_j exists such that $(g_i, g_j) \in dG$, then:

$$p(g_j = 1)^{k+1} = p(g_j = 1)^k + sign(g_i, g_j) * \varepsilon * p(g_j = 1)^k$$

Where $p(g_j = 1)^k$ is the probability of mutating the gene g_j at iteration k, and ε is a parameter indicating the amount by which a probability is increased or decreased. Lastly, all non-zero probabilities are rescaled to sum to 1. This procedure is repeated until the number of mutations in the sample is equal to its predefined tumor mutation burden.

Results presented in this manuscript were based on an artificial dataset comprising 1000 tumor samples with a number of mutations varying between 5 and 30, across 100 genes, with mutation frequencies varying between 0.05 and 0.2. In total, 30 gene pairs were selected as interdependent, with 15 synergistic interactions, giving rise to 15 co-occurrently mutated gene pairs, and 15 antagonistic interactions, giving rise to 15 gene pairs mutated in a mutually exclusive manner. We set $\varepsilon = 0.95$, but highly similar results were obtained with $\varepsilon = 0.75, 0.85, 0.95$.

To demonstrate that this model effectively generates co-occurrent and mutually exclusive mutation pairs consistent with the predefined set of interdependencies, we generated a second dataset of the same size, using the same gene mutation frequencies and tumor mutation burdens, but without specifying any interdependent gene pairs (i.e. $dG = \{\emptyset\}$). By comparing the co-mutation incidence of each gene pair in the two datasets, we could see that co-mutations incidences differ substantially only among gene pairs that were set as interdependent in the first dataset (**Extended Data Fig. 7C**). Since, these differences were not constant among gene pairs and especially for gene pairs with low mutation frequencies, differences among co-mutation incidences were smaller, we decided to assign a weight to each co-mutation in the precision and recall analysis shown in **Fig. 1B**, such that each tool is penalized less if it fails to recover a true interdependent gene pair whose co-mutation incidence was only moderately different from the expected one. Precisely, the weight of a co-mutation equals the absolute difference between the observed (in the model with true interdependent gene pairs) and expected (averaged across the 100 generated models without any interdependent gene pairs) number of samples where the two genes are co-mutated. All weights were scaled by the maximal weight across all gene pairs.

In the benchmarking on artificial dataset, we excluded the tool Coselens, since it requires MAF (mutation annotation file) as input, rather than a genome alteration matrix, like that generated by our model.

<u>Co-mutations analysis using synonymous variants.</u> We ran SelectSim, SELECT, DISCOVER, weSME/CO, cooccur, and GAMToC on a GAM built from the TCGA pan-cancer using only synonymous mutations across 396 cancer-associated genes. Genes mutated in less than 9 samples were removed. Coselens could not be used in this analysis since it already uses synonymous variants as input.

<u>Permutation vs. Simulation analysis.</u> The comparison between the time required for GAM permutations and GAM simulations was done independently of the tools adopting these approaches, using custom scripts that isolate these specific steps. For GAM permutations, we used the BiRewire R package[11] that was previously adopted in SELECT[5]. BiRewire is a highly optimized library that implements edge rewiring of a graph while preserving node degrees[12], and it was proposed to optimize the edge-switching technique introduced for binary matrix permutations that preserve the marginals of both rows and columns[13]. GAM simulations were implemented using a custom R script. The computational time analysis was performed 3 times for each approach, each time requiring the generation of 100 random matrices and taking as input: the default GAM provided with the BiRewire package or a series of random GAMs containing 500 genes and a number of samples varying from 500 to 10000.

<u>DISCOVER and SelectSim comparison.</u> DISCOVER determines significant mutual exclusivity and co-occurrence by estimating the probability of a gene being mutated in a sample using a similar

approach to the one described for SelectSim. However, in DISCOVER, the observed and predicted number of co-mutations are not weighted by the individual sample TMB, unlike in SelectSim. As a result, DISCOVER did not report significant co-occurrence among cancer mutations. To test if this could be due to the weighting strategy, we extracted the mutation probability generated by DISCOVER and used it to generate simulation matrices, which we could use to run SelectSim with sample weighting. These analyses were performed in parallel with SelectSim, the original version of DISCOVER, and the modified version of DISCOVER using the same LUAD dataset as input.

Co-mutation analysis for OncoKB and Random genes. First, we calculated the total number of recurrent missense mutations for all genes sequenced in TCGA, defined as missense mutations occurring at the same residue of the same gene in at least 2 samples. Next, we selected a set of genes within our catalog of 396 cancer-associated genes (*cancer genes*) and a set of randomly picked genes not included in our catalog (*random genes*), such that for each cancer gene with *n* number of putative oncogenic missense mutation (based on OncoKB), a random gene exists with *n* number of recurrent missense mutations. This led to the selection of 172 cancer genes and 173 random genes with matching alteration frequencies. Genomic alteration matrices (GAMs) were built for each gene set and TCGA samples. SelectSim was run independently on each GAM.

Admixture Co-mutation analysis for oncogenic and silent mutations. We filtered TCGA dataset to retain only missense and synonymous mutations for downstream analyses. Mutations were defined as “putative neutral” if synonymous or if missense but not classified as “oncogenic” or “likely oncogenic in OncoKB. Missense mutations were defined as putative oncogenic if classified as “oncogenic” or “likely oncogenic in OncoKB. Next, we generated a series of datasets including different proportions of putative neutral and putative oncogenic variants. In each dataset, a proportion (K%) of putative oncogenic mutations in each gene was replaced with an equal proportion (or the maximum available) of putative neutral variants in that gene. Datasets were generated for K = 100, 90, 80, 70, 50, 30, 20, 10, and 0. SelectSim was run independently on each dataset.

REFERENCES

1. Skoulidis, F. *et al.* Co-occurring Genomic Alterations Define Major Subsets of KRAS-Mutant Lung Adenocarcinoma with Distinct Biology, Immune Profiles, and Therapeutic Vulnerabilities. *Cancer Discov.* **5**, 860–877 (2015).
2. Stefanoudakis, D. *et al.* Significance of TP53, CDKN2A, SMAD4 and KRAS in Pancreatic Cancer. *Curr. Issues Mol. Biol.* **46**, 2827–2844 (2024).
3. Jee, J. *et al.* Automated real-world data integration improves cancer outcome prediction. *Nature* **636**, 728–736 (2024).
4. Mina, M., Iyer, A. & Ciriello, G. Epistasis and evolutionary dependencies in human cancers. *Curr. Opin. Genet. Dev.* **77**, 101989 (2022).
5. Mina, M. *et al.* Conditional Selection of Genomic Alterations Dictates Cancer Evolution and Oncogenic Dependencies. *Cancer Cell* **32**, 155-168.e6 (2017).
6. Canisius, S., Martens, J. W. M. & Wessels, L. F. A. A novel independence test for somatic alterations in cancer shows that biology drives mutual exclusivity but chance explains most co-occurrence. *Genome Biol.* **17**, 261 (2016).
7. Kim, Y.-A., Madan, S. & Przytycka, T. M. WeSME: uncovering mutual exclusivity of cancer drivers and beyond. *Bioinformatics* **33**, 814–821 (2017).
8. Griffith, D. M., Veech, J. A. & Marsh, C. J. cooccur: Probabilistic Species Co-Occurrence Analysis in R. *J. Stat. Softw.* **69**, 1–17 (2016).
9. Iranzo, J., Gruenhagen, G., Calle-Espinosa, J. & Koonin, E. V. Pervasive conditional selection of driver mutations and modular epistasis networks in cancer. *Cell Rep.* **40**, 111272 (2022).
10. Melamed, R. D., Wang, J., Iavarone, A. & Rabadan, R. An information theoretic method to identify combinations of genomic alterations that promote glioblastoma. *J. Mol. Cell Biol.* **7**, 203–213 (2015).
11. Gobbi, A. *et al.* Fast randomization of large genomic datasets while preserving alteration counts. *Bioinformatics* **30**, i617–i623 (2014).
12. Milo, R., Kashtan, N., Itzkovitz, S., Newman, M. E. J. & Alon, U. On the uniform generation of random graphs with prescribed degree sequences. Preprint at https://doi.org/10.48550/arXiv.cond-mat/0312028 (2004).
13. Ciriello, G., Cerami, E., Sander, C. & Schultz, N. Mutual exclusivity analysis identifies oncogenic network modules. *Genome Res.* **22**, 398–406 (2012).
