# Supplementary material for: Evolving patterns of co-mutations from tumor initiation to metastatic progression
Source: Nat Genet. 2026 Jul 10;58(8):2053–62. doi: 10.1038/s41588-026-02661-4 (PMC13447111; doi:10.1038/s41588-026-02661-4)
Supplement: Supplementary file 2 [file 41588_2026_2661_MOESM2_ESM.pdf]

nature portfolio

Corresponding author(s): Giovanni Ciriello

Last updated by author(s): Apr 21, 2026

Reporting Summary

Nature Portfolio wishes to improve the reproducibility of the work that we publish. This form provides structure for consistency and transparency in reporting. For further information on Nature Portfolio policies, see our Editorial Policies and the Editorial Policy Checklist.

Statistics

For all statistical analyses, confirm that the following items are present in the figure legend, table legend, main text, or Methods section.

n/a	Confirmed	
☐	☒	The exact sample size (n) for each experimental group/condition, given as a discrete number and unit of measurement
☒	☐	A statement on whether measurements were taken from distinct samples or whether the same sample was measured repeatedly
☐	☒	The statistical test(s) used AND whether they are one- or two-sided<br>*Only common tests should be described solely by name; describe more complex techniques in the Methods section.*
☐	☒	A description of all covariates tested
☐	☒	A description of any assumptions or corrections, such as tests of normality and adjustment for multiple comparisons
☐	☒	A full description of the statistical parameters including central tendency (e.g. means) or other basic estimates (e.g. regression coefficient) AND variation (e.g. standard deviation) or associated estimates of uncertainty (e.g. confidence intervals)
☐	☒	For null hypothesis testing, the test statistic (e.g. F, t, r) with confidence intervals, effect sizes, degrees of freedom and P value noted<br>*Give P values as exact values whenever suitable.*
☒	☐	For Bayesian analysis, information on the choice of priors and Markov chain Monte Carlo settings
☒	☐	For hierarchical and complex designs, identification of the appropriate level for tests and full reporting of outcomes
☐	☒	Estimates of effect sizes (e.g. Cohen's d, Pearson's r), indicating how they were calculated

Our web collection on statistics for biologists contains articles on many of the points above.

Software and code

Policy information about availability of computer code

Data collection	No software was used. All data were collected by third parties.
Data analysis	Multiple R (v. 4.3.2) packages were used in the analysis (cf. GitHub repository linked below). The SelectSim is implemented as an R Package and is available at: https://github.com/CSOgroup/SelectSim and https://doi.org/10.5281/zenodo.19680236. Additional scripts to reproduce the primary analyses presented in this manuscript are available here: https://github.com/CSOgroup/SelectSim_analysis.

For manuscripts utilizing custom algorithms or software that are central to the research but not yet described in published literature, software must be made available to editors and reviewers. We strongly encourage code deposition in a community repository (e.g. GitHub). See the Nature Portfolio guidelines for submitting code & software for further information.

Data

Policy information about availability of data

All manuscripts must include a data availability statement. This statement should provide the following information, where applicable:

- Accession codes, unique identifiers, or web links for publicly available datasets
- A description of any restrictions on data availability
- For clinical datasets or third party data, please ensure that the statement adheres to our policy

We retrieved publicly available mutational data for The Cancer Genome Atlas (TCGA) dataset from https://gdc.cancer.gov/about-data/publications/Pathways-2018, and for The American Association for Cancer Research (AACR) Project Genomics Evidence Neoplasia Information Exchange cohort (version 15) from https://

doi.org/10.7303/syn53210170. Data for the MSK-CHORD dataset was retrieved from: https://www.cbioportal.org/study/summary?id=msk_chord_2024. Additional datasets were collected from the supplementary material of the corresponding publications. Supplementary Data 1 (genome alteration matrices of the GENIE cohorts) and Supplementary Data 2 (preprocessed input for SelectSim of all used cohorts) are available at: https://doi.org/10.5281/zenodo.13752869 . Additional data including input and results of the analyses in this manuscript are available at: https://doi.org/10.5281/zenodo.13769785 .

Research involving human participants, their data, or biological material

Policy information about studies with human participants or human data. See also policy information about sex, gender (identity/presentation), and sexual orientation and race, ethnicity and racism.

Reporting on sex and gender	Sex and gender were not analyzed, neither used an inclusion or exclusion criteria.
Reporting on race, ethnicity, or other socially relevant groupings	Race, ethnicity, or other socially relevant groupings were not analyzed, neither used an inclusion or exclusion criteria.
Population characteristics	The relevant covariate for categorization of samples is the type of tumor (primary or metastasis) as given by annotations in the TCGA and AACR-GENIE cohorts.
Recruitment	All data that was used is publicly available.
Ethics oversight	All data that was used is publicly available under The Cancer Genome Atlas program and The American Association for Cancer Research (AACR) GENIE project.

Note that full information on the approval of the study protocol must also be provided in the manuscript.

Field-specific reporting

Please select the one below that is the best fit for your research. If you are not sure, read the appropriate sections before making your selection.

☒ Life sciences ☐ Behavioural & social sciences ☐ Ecological, evolutionary & environmental sciences

For a reference copy of the document with all sections, see nature.com/documents/nr-reporting-summary-flat.pdf

Life sciences study design

All studies must disclose on these points even when the disclosure is negative.

Sample size	No sample-size calculation was performed. We used all available samples (after data exclusions described in the Methods section) from the TCGA dataset (~9,000 samples) and GENIE datasets (MSK Primary: ~28,000; MSK Metastasis: ~15,000; DFCI Primary: ~14,500; DFCI Metastasis: ~7,500). The sizes of these datasets were sufficient for all statistical analyses.
Data exclusions	For TCGA dataset, samples with no clear tumor lineage annotation were excluded as described in Mina et al. (2020). For GENIE datasets, samples without clear tumor type or primary/metastasis annotations were excluded; also, we excluded samples coming from patients with multiple samples, and samples of tumor types with less than 100 samples, as described in the Methods section.
Replication	Independent cohorts and datasets (one from TCGA and two from GENIE) were used to assess the reproducibility of the results. These analyses are presented and discussed in the main text. No new experiments were performed, as data come from public repositories.
Randomization	Randomization procedures are intrinsic to the algorithmic and statistical approaches adopted and presented in the Methods section.
Blinding	N/A. This work does not include any new experiment.

Reporting for specific materials, systems and methods

We require information from authors about some types of materials, experimental systems and methods used in many studies. Here, indicate whether each material, system or method listed is relevant to your study. If you are not sure if a list item applies to your research, read the appropriate section before selecting a response.

Materials & experimental systems

n/a	Involved in the study
☒	☐ Antibodies
☒	☐ Eukaryotic cell lines
☒	☐ Palaeontology and archaeology
☒	☐ Animals and other organisms
☒	☐ Clinical data
☒	☐ Dual use research of concern
☒	☐ Plants

Methods

n/a	Involved in the study
☒	☐ ChIP-seq
☒	☐ Flow cytometry
☒	☐ MRI-based neuroimaging

Plants

Seed stocks	*Report on the source of all seed stocks or other plant material used. If applicable, state the seed stock centre and catalogue number. If plant specimens were collected from the field, describe the collection location, date and sampling procedures.*
Novel plant genotypes	*Describe the methods by which all novel plant genotypes were produced. This includes those generated by transgenic approaches, gene editing, chemical/radiation-based mutagenesis and hybridization. For transgenic lines, describe the transformation method, the number of independent lines analyzed and the generation upon which experiments were performed. For gene-edited lines, describe the editor used, the endogenous sequence targeted for editing, the targeting guide RNA sequence (if applicable) and how the editor was applied.*
Authentication	*Describe any authentication procedures for each seed stock used or novel genotype generated. Describe any experiments used to assess the effect of a mutation and, where applicable, how potential secondary effects (e.g. second site T-DNA insertions, mosiacism, off-target gene editing) were examined.*
