# Supplementary material for: Evolving patterns of co-mutations from tumor initiation to metastatic progression
Source: Nat Genet. 2026 Jul 10;58(8):2053–62. doi: 10.1038/s41588-026-02661-4 (PMC13447111; doi:10.1038/s41588-026-02661-4)
Supplement: Supplementary file 3 [file 41588_2026_2661_MOESM3_ESM.pdf]

nature portfolio

Peer Review File

Evolving patterns of co-mutations from tumor initiation to metastatic progression

Corresponding Author: Professor Giovanni Ciriello

This file contains all editorial decision letters in order by version, followed by all author rebuttals in order by version.

Version 0:

Decision Letter:

27th Mar 2025

Dear Professor Ciriello,

Your Analysis, "Evolving patterns of co-mutations from tumor initiation to metastatic progression" has now been seen by 3 referees. You will see from their comments below that while they find your work of interest, some important points are raised. We are interested in the possibility of publishing your study in Nature Genetics, but would like to consider your response to these concerns in the form of a revised manuscript before we make a final decision on publication.

We therefore invite you to revise your manuscript taking into account all reviewer and editor comments. Please highlight all changes in the manuscript text file. At this stage we will need you to upload a copy of the manuscript in MS Word .docx or similar editable format.

We are committed to providing a fair and constructive peer-review process. Do not hesitate to contact us if there are specific requests from the reviewers that you believe are technically impossible or unlikely to yield a meaningful outcome.

When revising your manuscript:

*1) Include a “Response to referees” document detailing, point-by-point, how you addressed each referee comment. If no action was taken to address a point, you must provide a compelling argument. This response will be sent back to the referees along with the revised manuscript.

*2) If you have not done so already please begin to revise your manuscript so that it conforms to our Analysis format instructions, available
here.
Refer also to any guidelines provided in this letter.

*3) Include a revised version of any required Reporting Summary: https://www.nature.com/documents/nr-reporting-summary.pdf
It will be available to referees (and, potentially, statisticians) to aid in their evaluation if the manuscript goes back for peer review.
A revised checklist is essential for re-review of the paper.

Please be aware of our guidelines on digital image standards.

EXTENDED DATA FIGURES

When re-submitting your manuscript, please ensure that any supplementary figures and tables that are crucial to the manuscript’s conclusions are converted into Extended Data figures and tables to increase visibility of these data. Extended Data figures and tables are online-only (present in the online PDF and full-text HTML versions of the paper), peer-reviewed display items that provide essential background to the article but are not included in the main article due to space

constraints. A maximum of ten Extended Data display items (figures and tables) is permitted.

Please use the link below to submit your revised manuscript and related files:

Link Redacted

Note: This URL links to your confidential home page and associated information about manuscripts you may have submitted, or that you are reviewing for us. If you wish to forward this email to co-authors, please delete the link to your homepage.

We hope to receive your revised manuscript within four to eight weeks. If you cannot send it within this time, please let us know.

Please do not hesitate to contact me if you have any questions or would like to discuss these revisions further.

Nature Genetics is committed to improving transparency in authorship. As part of our efforts in this direction, we are now requesting that all authors identified as ‘corresponding author’ on published papers create and link their Open Researcher and Contributor Identifier (ORCID) with their account on the Manuscript Tracking System (MTS), prior to acceptance. ORCID helps the scientific community achieve unambiguous attribution of all scholarly contributions. You can create and link your ORCID from the home page of the MTS by clicking on ‘Modify my Springer Nature account’. For more information please visit please visit www.springernature.com/orcid.

We look forward to seeing the revised manuscript and thank you for the opportunity to review your work.

Sincerely,

Safia Danovi, PhD
Senior Editor, Nature Genetics
ORCiD: 0009-0007-7822-5479

Referee expertise:

Referee #1: cancer genomics, computational

Referee #2: cancer genomics

Referee #3: cancer genomics, computational, metastasis

Reviewers' Comments:

Reviewer #1 (Remarks to the Author):

Iyer et al examines co-mutational occurrence across a large collection of public cancer datasets. Even though the article attempts to solve some bottlenecks in co-exclusivity and co-occurrence analysis it is hard to call the approaches to these problems and results novel, as they have been extensively studied across multiple publications and cancer genomic consortia. Tools for permutation or simulation based co-occurrence analysis had been used for many years and even exists in public cancer online portals, such as cBioPortal. Most of the earlier tests are well calibrated over Q-Q space and are not at all prohibitive computationally. Based on novelty and conceptual advances this study does not seem to be appropriate for Nat Genetics, but rather for a more specialized bioinformatics journal.

Reviewer #2 (Remarks to the Author):

In the manuscript “Evolving patterns of co-mutations from tumor initiation to metastatic progression” Iyer et al. present a new approach to analyze the patterns of co-mutations, i.e. pairs of genes that undergo mutation (or lack of) at a frequency that significantly deviates from what would be expected assuming independent mutational propensity and selection effects across genes.

The study of co-mutations is essential for better understanding the somatic evolutionary trajectories towards tumorigenesis and metastasis, with a clear potential to discover new cancer vulnerabilities and prognostic biomarkers, thereby improving therapy in the clinic. Furthermore, the evidence provided by recent sequencing studies of healthy tissues, revealing not just the presence of cancer mutations but also the emergence of not yet well understood clonal dynamics driven by those mutations, demands more nuanced genomic characterizations to understand the effects of these variants in the context of somatic evolution in normal tissues as well as their role in the progression towards premalignancy and cancer. In this regard, the goal of the paper is central to cancer genomics.

The manuscript presents SelectSim, a new method to identify co-mutations from catalogs of mutations obtained from cohorts of sequenced samples, which the authors put to use to characterize co-mutation patterns in tumors, differences between primary and metastatic tumors, whether these differences bear prognostic value as well as differences between the co-mutation patterns arising in normal urothelium and bladder cancer. To address these questions the authors make use of whole-exome and panel sequencing data in tumors (MSK, DFCI) and whole-exome sequencing of laser microdissected samples from morphologically normal urothelium.

The study is well conducted, providing a sound rationale and adequate controls. The study with tumors, both the atlas and primary vs metastasis, is robust and underscores the value of massive collection, sequencing and annotation of tumor samples in the clinic. Although it hints towards intriguing leads of tumor initiation patterns, the sequencing study with the MNU dataset is not conclusive. The manuscript is well-written and the analyses and results are described clearly. However, there are a few important aspects of the study that would require further clarification or justification.

Main comments:

The study asks whether the readout SelectSim is mainly determined by selection and devises an analysis using GAMs based on a large set of driver genes with putative oncogenic vs synonymous mutations. How different are the respective GAMs in terms of sparsity or total counts? How much can this difference affect the comparison?

In general, how does sparsity or total count determine the readout of SelectSim? If true, would it then make sense to downsample so that the levels of sparsity are comparable when comparing SelectSim readouts?

In applying the filter of potentially oncogenic mutations across the chosen panel of genes, how many putative driver mutations does each sample have? How much of a problem can it be to annotate as potentially oncogenic mutations found in genes with no known driver association in the tumor type of the sample? Would it be a good idea to mask those counts?

How does the counts of putative driver mutations compare with the expected number of driver mutations per sample according to prior knowledge about the number of driver mutations per sample? This can be a simple though compelling sanity check.

Expanding on this, I wonder whether the synonymous mutation data per gene can be exploited to assess the amount of positive selection, e.g. via dN/dS type of approach, to provide another level of assessment of whether the total number of putative driver mutations per gene might be inflated, hence corrected.

If the number of putative drivers accounted for is too high, that would mean that neutral or passenger mutations leak into the model. How much then may the neutral mutation heterogeneity confound the analysis of co-mutations? The method's expectation is made proportional across genes and samples, but this proportionality might not hold neutral mutation wise.

To what extent the results could be affected by the fact that there are different driver mechanisms implying that some genes require a single hit while others require a double hit, with one of the hits not necessarily being a mutation, e.g. deletion? Should (and how) the observed frequencies at each driver gene be adjusted for the expected mechanism of action of the driver gene?

The fact that large genes such as TTN and MUC16 show up as significant co-mutations in the randomly generated gene set, highlights the concerns made in my two previous comments: for one, whether the putative oncogenicity filter allows too many mutations, thereby leaking neutral mutation counts in the GAMs; for second, the fact that double hits representing a single oncogenic event may make a difference in the SelectSim readout.

In silico simulation of GAMs (l.806-807). I found this sampling method somewhat counterintuitive. For example, how would that differ from drawing from a Poisson or negative binomial distribution, then binarizing the integer output? How do the dispersions compare?

Assuming that the mutation catalogs from MSK and DFCI contain germline mutations, the co-mutation analysis involving this dataset implies a mix of cancer predisposition variants and somatic mutations. I wonder whether any significant co-mutations found upon this dataset are enriched in germline mutations, pointing towards early driver events in the multistage tumorigenic transformation process.

Although rooted in intuition, the conceptualization of the term "trajectory" is vague. The manuscript should provide some sort of formal or operational definition.

The prognostic value of co-mutations as biomarkers of metastasis is original and convincing. I wonder whether these prognostic effects result via an interaction with the type of treatment of the primary tumor, or else they are predictors regardless of the treatment.

Why did authors not include the sequencing study of normal urothelial cells by Lawson et al? Would the results yielded by the MNU analysis reproduce? This would provide solid support for the main takes of this otherwise interesting part of the study.

Although one of the justifications for sequencing LCMs is to call somatic mutations from oligoclonal samples, there is still a

chance for low VAF clones to escape detection. Claiming that co-mutations are not present may be oblivious of the fact that the co-mutation might be there albeit in small clones.

Regarding the comparison with bladder tumors, it might be a good idea, in order to understand the tumor promoting (or protecting) role of co-mutations and their involvement in early vs late tumor evolutionary trajectories, to stratify the bladder tumors into non-muscle-invasive and muscle-invasive subtypes.

Minor comments:

How is the random gene generation (featured in Figure 1D, third panel) done?
Figure 2F, what does a connection between two gene sets mean?
Figure 4E are the donor labels right?
I would suggest to use another notation for the outer product, rather than “x”
l.803-804: what does (E)=1 mean?
l.820: how is the m_i value specified? this sentence is very confusing, please clarify
Why 80% subsampling in the metastasis vs primary analysis?
Supplementary fig 2A: APC-TP53 colorectal cancer? What are the grey and black dots? non-significant vs significant? What is going on in the case of colorectal cancer? Can this apparently incoherent TP53-APC co-mutation be attributed to mixed tumor subtypes?

Reviewer #3 (Remarks to the Author):

This paper introduces SelectSim, a method to identify pairs of somatic mutations that are either signficantly co-occurring or signficantly mutually exclusive. Briefly, SelectSim takes as input a binary sample x gene matrixand for each pair of mutations computes an effect size (negative for mutex, positive for co-occuring, 0 for no effect) and statistical significance. This is done by simulating alternative binary matrices from a null model derived from the data. Rather than using a permutation test or computing p-values exactly, SelectSim generates alternative binary matrices (without stringently enforcing row/column marginals). The results of real data are strong. My main criticism is that the method and underlying null model needs to be better described, and that a simulation study (with known ground truth) is warranted, especially given the heuristic nature of SelectSim.

1. Better description of the method

- It seems that the method is attempting to generate alternative binary marices under the assumption of independence among genes and samples while maintaining gene mutation frequences and tumor mutational burdens (for samples). In other words, sample binary matrices with fixed row and column marginals. It would be good to state this upfront in Methods. Also, it is important to discuss current approaches for sampling such constrained matrices (e.g. MCMC based on the swap/knight's tour algorith, or sequential importance sampling.) Then, please discuss how SelectSim differs from these standard approaches.

- Additionally, I found the mathematical description missing important definitions.

- line 787: define TMB(t_j). Is it \sum_{g \in G} GAM(g,t_j)?

- line 798 (and also later): Use \times rather than the letter x to indicate matrix multiplication.

- line 803: "We set (E) = 1." -- What does this mean?

- line 820: what is m_i? The current description is unclear.

- Finally, I would like to see a more intuitive description of why substracting uniform distribution from the expected mutation frequencies (obtained assuming indepedent and distinct Bernoulli random variables) followed by binarization is a reasonable approximation for obtaining binary matrices adhering to fixed marginals.

2. Experimental evaluation using simulations with known ground truth

- I think the paper would be greatly improved if there would be a simulation study with ground-truth pairs of mutually exclusive and/or co-occurring pairs of mutations. While I appreciate the comparison on real data (with down sampling experiments), I think a more direct comparison to ground truth would be more compelling. Ideally such a comparison would be used to compare against competing methods as well.

- Please use simulations to assess the effect of the different parameters and threshold choices of SelectSim (such as \tau and \lambda, as well as the four categories listed in line 925).

2. More detailed comparisons against standard approaches of generating binary matrices with fixed marginals.

- The paper mentions a permutation-based approach for generating binary matrices with fixed row and column marginals (BiRewire). The only results I could find is a modest reduction (by at most a factor of 2) in running time. What about the

results? Are they in agreement? Ideally, this would be assessed with simulations with known ground truth.

3. More detailed discussion of the limitations of the approach.

- Beyond pairs

- Intra-tumor heterogeneity, subclonal mutations may confound analysis

- How to more naturally account for difference between mutation types

- Power analysis?

- Mutation level instead of gene level

Minor:

- what is the difference between "statistically signifcant reccurent mutations" (line 60) and "more or less frequent than expected by chance" (line 63)?

- line 76: include citations after "few approaches"

- Include a feature table to discuss features and limitations of SelectSim as well as other methods. There are approaches that consider fixed row and column sums. One such approach is:

[1] Mark DM Leiserson, Matthew A Reyna, Benjamin J Raphael, A weighted exact test for mutually exclusive mutations in cancer, Bioinformatics, Volume 32, Issue 17, September 2016, Pages i736–i745, https://doi.org/10.1093/bioinformatics/btw462

Version 1:

Decision Letter:

2nd Sep 2025

Dear Professor Ciriello,

Your Analysis, "Evolving patterns of co-mutations from tumor initiation to metastatic progression" has now been seen by 3 referees. You will see from their comments below that while they find your work of interest, some important points are raised. We are interested in the possibility of publishing your study in Nature Genetics, but would like to consider your response to these concerns in the form of a revised manuscript before we make a final decision on publication.

We therefore invite you to revise your manuscript taking into account all remaining reviewer comments. Please highlight all changes in the manuscript text file. At this stage we will need you to upload a copy of the manuscript in MS Word .docx or similar editable format.

We are committed to providing a fair and constructive peer-review process. Do not hesitate to contact us if there are specific requests from the reviewers that you believe are technically impossible or unlikely to yield a meaningful outcome.

When revising your manuscript:

*1) Include a “Response to referees” document detailing, point-by-point, how you addressed each referee comment. If no action was taken to address a point, you must provide a compelling argument. This response will be sent back to the referees along with the revised manuscript.

*2) If you have not done so already please begin to revise your manuscript so that it conforms to our Analysis format instructions, available
here.
Refer also to any guidelines provided in this letter.

*3) Include a revised version of any required Reporting Summary: https://www.nature.com/documents/nr-reporting-summary.pdf
It will be available to referees (and, potentially, statisticians) to aid in their evaluation if the manuscript goes back for peer review.
A revised checklist is essential for re-review of the paper.

Please be aware of our guidelines on digital image standards.

EXTENDED DATA FIGURES

When re-submitting your manuscript, please ensure that any supplementary figures and tables that are crucial to the manuscript's conclusions are converted into Extended Data figures and tables to increase visibility of these data. Extended Data figures and tables are online-only (present in the online PDF and full-text HTML versions of the paper), peer-reviewed display items that provide essential background to the article but are not included in the main article due to space constraints. A maximum of ten Extended Data display items (figures and tables) is permitted.

Please use the link below to submit your revised manuscript and related files:

Link Redacted

Note: This URL links to your confidential home page and associated information about manuscripts you may have submitted, or that you are reviewing for us. If you wish to forward this email to co-authors, please delete the link to your homepage.

We hope to receive your revised manuscript within four to eight weeks. If you cannot send it within this time, please let us know.

Please do not hesitate to contact me if you have any questions or would like to discuss these revisions further.

Nature Genetics is committed to improving transparency in authorship. As part of our efforts in this direction, we are now requesting that all authors identified as 'corresponding author' on published papers create and link their Open Researcher and Contributor Identifier (ORCID) with their account on the Manuscript Tracking System (MTS), prior to acceptance. ORCID helps the scientific community achieve unambiguous attribution of all scholarly contributions. You can create and link your ORCID from the home page of the MTS by clicking on 'Modify my Springer Nature account'. For more information please visit please visit www.springernature.com/orcid.

We look forward to seeing the revised manuscript and thank you for the opportunity to review your work.

Sincerely,

Safia Danovi, PhD
Senior Editor, Nature Genetics
ORCiD: 0009-0007-7822-5479

Referee expertise:

Referee #1:

Referee #2:

Referee #3:

Reviewers' Comments:

Reviewer #1 (Remarks to the Author):

I appreciate the authors detailed answer to earlier comments. I certainly appreciate the authors expertise in co-mutational analysis. New normal tissue co-mutational analyses is novel.
What still remains unclear from the manuscript and responses to comments:
1) What is the conceptual advances in SelectSim that were not available in the 2020 Nat Gen SELECT paper? Some performance improvement? It would be great to see this in very specific terms of the model changes and math.

2) AF or CCF are not employed as a covariate, though it would seem as very important. Purity of the sample (not used) can also lead to falsely missed co-cooccurrence patterns. Those are main drivers of heterogeneity.

3) Authors mention that mutational processes/signatures co-variates are implied, but are not really discussed in the manuscript. No known mutational processed are used even if available for some cohorts.

4) Given extensive prior work in the field and by the current group on the topic it is very important to highlight in the text and discussion what has been known as interaction between mutations, what is found due to the novel model presented, what is found just by the sheer increase in available data to 70k tumors. Very importantly authors mention incorrect results from other studies that are rectified with the new approaches - could those be enumerated and explained - this seems very important to correct the field if this is the case.

5) Would be great if survival analysis/ coxph is accomplished in regards to co-occurrence on at least the cohorts that have clinical annotations.

6) Are metastasis specific pairs due to progression? or they really are mutually exclusive first and then become co-mutated?

7) The field has always been interested in more than pairs - i.e. triplets, quads. Does this method and new large dataset answer those questions?

8) Would be great to see detailed Q-Q plots for all scenarios.

9) discussion should be expanded to cover all the above topics and really highlight important novel findings

Reviewer #1 (Remarks on code availability):

The code repo seemed to have not been updated during revision?

Reviewer #2 (Remarks to the Author):

General assessment

The manuscript has been substantially revised, with clear improvements compared with the previous version. The performance analysis of the co-mutation method is more robust. The study with somatic mutations in normal tissues, which has been completely reformulated, is more compelling.

The study does not only provide a tool that is bound to be of practical utility in somatic evolution genomic studies, but it also portrays a relevant case in the form of metastasis risk assessment based on differential co-mutation enrichments, and it also provides a novel and in this reviewer's opinion important insight into the question of whether and to which extent the somatic co-mutation patterns in normal tissues recapitulate or resemble the genomic features known in tumor samples.

The results of the normal tissue study make sense in the light of a multistage model of carcinogenesis whereby a relatively limited repertoire of sequential genomic events would allow the carcinogenic transformation, suggesting that in normal tissues a significantly more diverse repertoire of mutant combinations are possible, hinting towards a model whereby the clonal landscape is dominated by competition and convergent evolution. Unlike with the first review, I am more convinced that the results of this analysis are at the same time robust enough and interesting in the context of somatic genomic studies.

The authors have addressed to a substantial extent the concerns and comments raised by the reviewers. This reviewer is overall satisfied with the answers provided to their comments in the first review. I commend the authors for their work in improving the study and thoroughly replying to the reviews.

I noted down a couple of additional comments which would not necessarily require a direct answer – as far as this reviewer is concerned – but would nevertheless appreciate if the authors and editors consider them for the sake of improvement, should the manuscript be published.

Specific additional comments

- Regarding the regression analysis of metastatic risk, I wonder whether encoding the treatment into 40+ treatment types may be slicing the treatment factor too thinly for any single feature or set of features to show a consistent effect. One alternative would be grouping into broader treatment categories, which could also be tricky, given the heterogeneity of treatments and therapeutic effects, but also considering the critical importance of treatment combinations and regiment. The authors should consider, before drawing a negative conclusion regarding the treatment effects, whether the analysis can be conducted with broader treatment features classes to catch any potential interactions with treatment.

- Did the authors consider or conduct at some point this metastatic risk regression analysis by adjusting for age and/or some relevant factor or exposures such as smoking, whenever this metadata is available? I wonder whether there might be an age component, which the current analysis does not clarify, as to whether some co-mutations predict progression into metastasis. For example, do pro-metastatic co-mutations arise more frequently in older vs younger patients, or do they carry a stronger predictive effect in older vs younger patients, or vice versa? What about other exposures for which there is metadata in GENIE? I think it would be important to clarify, and I believe there is an opportunity to do so at little extra cost, if the clinical metadata permits, whether this risk effect is independent of age.

- Although the writing has improved – particularly when it comes to the method descriptions, where the previous version had some issues – there are still some parts that could be written more clearly. As an example to illustrate the type of concern, this reviewer found the explanation in p.23 where the fundamentals of the main statistical approach are laid out, still a bit troublesome. Despite the unavoidable subjective component of this assessment, I can point out to three problems that

permeate this part of the manuscript: i) Non-standard concepts. The notions of proposal and desired distribution, the concept of a fictitiously drawn sample, are not standard and were not particularly helpful for this reviewer. ii) Notational inconsistencies. S is given two non-equivalent definitions, confusing the audience: wouldn't it be preferable to just denote them differently? iii) Non-specific descriptions. The alluded construction with the plus/minus epsilon that pursues to attain some prescribed marginals by tuning the rejection threshold can (and should) be described in a completely univocal way.

Minor issues

- Two equations in page 22 have some typo / formatting errors
- Instead of "true", it feels more appropriate to use something like "ground truth" or "synthetic" to refer to data that you are simulating.
- Please, be mindful to always use an appropriate numerical scale for the axes of the plots, e.g. Supp Fig 6B

Reviewer #3 (Remarks to the Author):

I thank the authors for the detailed point-by-point response. My previous comments have been addressed. I have no further comments.

Reviewer #3 (Remarks on code availability):

I have reviewed the GitHub page. The README is clear, with clear installation instructions, and links to further documentation including an example. Simulations are available on zenodo.

Version 2:

Decision Letter:

8th Dec 2025

Dear Professor Ciriello,

Your Analysis, "Evolving patterns of co-mutations from tumor initiation to metastatic progression" has now been seen by 2 referees. You will see from their comments below that while they find your work of interest, some important points are raised. We are interested in the possibility of publishing your study in Nature Genetics, but would like to consider your response to these concerns in the form of a revised manuscript before we make a final decision on publication.

We therefore invite you to revise your manuscript taking into account all reviewer comments. Please highlight all changes in the manuscript text file. At this stage we will need you to upload a copy of the manuscript in MS Word .docx or similar editable format.

We are committed to providing a fair and constructive peer-review process. Do not hesitate to contact us if there are specific requests from the reviewers that you believe are technically impossible or unlikely to yield a meaningful outcome.

When revising your manuscript:

*1) Include a "Response to referees" document detailing, point-by-point, how you addressed each referee comment. If no action was taken to address a point, you must provide a compelling argument. This response will be sent back to the referees along with the revised manuscript.

*2) If you have not done so already please begin to revise your manuscript so that it conforms to our Analysis format instructions, available
here.
Refer also to any guidelines provided in this letter.

*3) Include a revised version of any required Reporting Summary: https://www.nature.com/documents/nr-reporting-summary.pdf
It will be available to referees (and, potentially, statisticians) to aid in their evaluation if the manuscript goes back for peer review.
A revised checklist is essential for re-review of the paper.

Please be aware of our guidelines on digital image standards.

EXTENDED DATA FIGURES

When re-submitting your manuscript, please ensure that any supplementary figures and tables that are crucial to the manuscript's conclusions are converted into Extended Data figures and tables to increase visibility of these data. Extended Data figures and tables are online-only (present in the online PDF and full-text HTML versions of the paper), peer-reviewed display items that provide essential background to the article but are not included in the main article due to space constraints. A maximum of ten Extended Data display items (figures and tables) is permitted.

Please use the link below to submit your revised manuscript and related files:

Link Redacted

Note: This URL links to your confidential home page and associated information about manuscripts you may have submitted, or that you are reviewing for us. If you wish to forward this email to co-authors, please delete the link to your homepage.

We hope to receive your revised manuscript within four to eight weeks. If you cannot send it within this time, please let us know.

Please do not hesitate to contact me if you have any questions or would like to discuss these revisions further.

Nature Genetics is committed to improving transparency in authorship. As part of our efforts in this direction, we are now requesting that all authors identified as 'corresponding author' on published papers create and link their Open Researcher and Contributor Identifier (ORCID) with their account on the Manuscript Tracking System (MTS), prior to acceptance. ORCID helps the scientific community achieve unambiguous attribution of all scholarly contributions. You can create and link your ORCID from the home page of the MTS by clicking on 'Modify my Springer Nature account'. For more information please visit please visit www.springernature.com/orcid.

We look forward to seeing the revised manuscript and thank you for the opportunity to review your work.

Sincerely,

Safia Danovi, PhD
Senior Editor, Nature Genetics
ORCiD: 0009-0007-7822-5479

Reviewers' Comments:

Reviewer #1 (Remarks to the Author):

I appreciate the authors's revisions and they addressed most of the previously raised points.
One last remaining point is

"""8) Would be great to see detailed Q-Q plots for all scenarios.
We have added detailed Q-Q plots for all datasets in the new Supplementary Figure 2B. """

The authors seem to have provided (Sup Fig 2B) a Q-Q plot vs Normal Distribution (or similar), this it's not what was asked - Please provide a proper plot(s) of observed p-values vs. expected uniform quantiles -log(p).
I am a bit surprised if the authors have not done it themselves previously as one of the best ways to check if the test is well-calibrated against the null model and that the p-values are not inflated/deflated. This is very important for us to be able to assess the test calibration properly. https://stats.stackexchange.com/questions/92124/how-to-interpret-a-qq-plot-of-p-values

Reviewer #2 (Remarks to the Author):

The authors have addressed all the concerns raised by the reviewers.

Version 3:

Decision Letter:

Our ref: NG-AN67697R2

12th Mar 2026

Dear Dr. Ciriello,

Thank you for submitting your revised manuscript "Evolving patterns of co-mutations from tumor initiation to metastatic progression" (NG-AN67697R2). It has now been seen by the original referees and their comments are below. The reviewers find that the paper has improved in revision, and therefore we'll be happy in principle to publish it in Nature Genetics, pending minor revisions to satisfy the referees' final requests and to comply with our editorial and formatting guidelines.

If the current version of your manuscript is in a PDF format, please email us a copy of the file in an editable format (Microsoft Word or LaTex)-- we can not proceed with PDFs at this stage.

We are now performing detailed checks on your paper and will send you a checklist detailing our editorial and formatting requirements soon. Please do not upload the final materials and make any revisions until you receive this additional information from us.

Thank you again for your interest in Nature Genetics Please do not hesitate to contact me if you have any questions.

Sincerely,

Cong Zhao, PhD
Research Cross-Journal Editorial Team
Nature Genetics
https://orcid.org/0009-0008-5833-6662

Editorial comments:

Author list: affiliations should be numbered (not annotated with letters)
Word counts: Ensure abstract, main text and figure legends comply with NG's standards (Abstract~150 words; Main text~ 4000 words; Methods~3,000 words; Main Figure legends & Supplementary Figures legends ~250 words).
Any very equation-heavy sections of Methods should be moved to the Supplementary Note. Excess text of Main text and Methods can be included as part of the Supplementary Note (not as a separate “Supplementary Methods” file). There's no word limit for Supplementary Note.
References should be split into those for the main text and methods-only
Add “Author contribution”
Code availability: add COI
Supplementary Figures (n=7) and Supplementary Note (n=1) should be combined into 1 PDF. Supplementary Tables (n=9) should be uploaded as one file “Supplementary Table” with multiple tabs.

Reviewer #1 (Remarks to the Author):

I appreciate the authors performing null-model adequacy check and diagnostics.
I have no further comments.

Open Access This Peer Review File is licensed under a Creative Commons Attribution 4.0 International License, which

permits use, sharing, adaptation, distribution and reproduction in any medium or format, as long as you give appropriate credit to the original author(s) and the source, provide a link to the Creative Commons license, and indicate if changes were made.
In cases where reviewers are anonymous, credit should be given to 'Anonymous Referee' and the source.
The images or other third party material in this Peer Review File are included in the article's Creative Commons license, unless indicated otherwise in a credit line to the material. If material is not included in the article's Creative Commons license and your intended use is not permitted by statutory regulation or exceeds the permitted use, you will need to obtain permission directly from the copyright holder.
To view a copy of this license, visit https://creativecommons.org/licenses/by/4.0/

Reviewers' Comments:

Reviewer #1 (Remarks to the Author):

Iyer et al examines co-mutational occurrence across a large collection of public cancer datasets. Even though the article attempts to solve some bottlenecks in co-exclusivity and co-occurrence analysis it is hard to call the approaches to these problems and results novel, as they have been extensively studied across multiple publications and cancer genomic consortia. Tools for permutation or simulation based co-occurrence analysis had been used for many years and even exists in public cancer online portals, such as cBioPortal. Most of the earlier tests are well calibrated over Q-Q space and are not at all prohibitive computationally. Based on novelty and conceptual advances this study does not seem to be appropriate for Nat Genetics, but rather for a more specialized bioinformatics journal.

As the reviewer pointed out, the problem of searching for "unexpected" co-mutation patterns has been tackled by several approaches (especially for what concerns mutually exclusive alterations), and this type of analyses have indeed been done in the context of large cancer genomics consortia, in particular TCGA and ICGC. We are very much aware of that, as we have worked on this problem for several years, both developing some of the first methods to search for mutually exclusive and co-occurrent alterations (see MEMo, ref: 2012 and SELECT refs: 2017, 2020), and we performed several of these analyses across multiple TCGA studies, including the TCGA pan-cancer atlas dataset (2018). Recently, we reviewed advances and still outstanding challenges in the field, discussing and comparing more than 20 distinct approaches (2022). This is however far from a solved problem. Major challenges still concern the high numbers of false positive results and misinterpretation due to inaccurate accounting of confounders (e.g., most tools work on individual tumor types and ignore heterogenous mutation rates), and the statistical power required for these analyses, which made impossible so far to gather sufficiently large independent datasets to validate the findings. Here, we discuss some of these problems, that we have better quantified in this revised version of the manuscript through new analyses, and highlight the main novelties introduced in this study, both in terms of method development and biological results.

From a methodological perspective, whereas several approaches have been developed to detect co-occurrent or mutually exclusive alterations, very few can detect both in an unbiased fashion and even less can account for covariates such as heterogeneous mutation rates, distinct tumor subtypes, and mutational processes. As highlighted in our recent review and in the benchmarking that we did in this study, only DISCOVER, SELECT (i.e., the tool that we previously developed), and SelectSim (the new tool that we present here) can account for heterogeneous tumor mutation burdens and tumor subtypes while searching for both co-occurrence and mutual exclusivity. DISCOVER however overestimates co-mutation rates in its null models and as such it fails to detect significant co-occurrences. In this revised version of the manuscript, we have introduced a **new model to generate artificial data** where we can impose evolutionary dependencies between gene pairs such that, if one gene in the pair acquires a mutation, the probability of mutating the second gene of the pair either increases or decreases. By generating artificial tumor samples with this model, evolutionary dependent pairs give rise to "true" co-occurrent and

mutually exclusive mutation patterns. We used this ground truth to compare 7 different approaches and demonstrate that SelectSim outperformed all of them in terms of precision and recall. Notably, almost all tools, demonstrated a high inflation of false positives obtaining good recall but very poor precision (see new **Figure 1B**). DISCOVER correctly detected the true mutually exclusive pairs, but again it failed to recover true co-occurrences, demonstrating that what was previously reported as a lack of co-occurrent mutations in cancer is actually a consequence of intrinsic limitations of this algorithm. Finally, we should note that what is implemented in the cBioPortal is actually a simple two-sided Fisher's test, which exclusively accounts for gene mutation frequencies, but *completely ignores heterogeneous tumor mutation rate and tumor subtypes* (the assumption is that all tumor samples are equally likely to have a mutation in the queried genes). We included the Fisher's test in our benchmarking against the ground truth, showing again the superior performance of SelectSim (**Figure 1B**). Overall, from a methodological point of view, SelectSim represents a significant advancement to the state-of-the-art, where current approaches are severely affected by false positives, especially in large and complex datasets where distinct tumor types and subtypes are admixed, with heterogeneous tumor mutation rates and mutational processes. *Analyzing these complex datasets is however critical to understand the impact of co-mutations on disease initiation and progression*.

From a cancer biology point of view, this study introduced 3 key novel results:

1. By integrating sequencing data for more than 70,000 tumor samples, we were able for the first time to <u>discover and validate</u> co-mutation patterns across multiple independent datasets, while retaining unprecedented statistical power. These results represent a **reference atlas of co-mutation patterns in human cancer** that simply didn't exist before and could have not been achieved with any of the other tools (indeed previous manuscripts in this line of research have typically focused on one or few tumor types, analyzed independently. Even our previous pan-cancer analyses were exclusively based on TCGA, which is considerably smaller than the meta-cohort that we analyzed here, and <u>did not include independent datasets for validation</u>).

2. Our comparison of co-mutation patterns between primary and metastatic samples (which became possible only with the large meta-cohort that we assembled) identified **new candidate biomarkers of disease progression**, which were confirmed in independent datasets. This analysis was highlighted and appreciated by the other reviewers and following one of their suggestions, now integrates treatment information as covariate. Even including treatment as covariate, *several co-mutations remain significant predictors confirming the results we initially presented*.

3. Lastly, we have significantly expanded the **co-mutation analysis in normal tissues**, comprising now more than 3500 samples from 44 donors and 3 major tissue types. This is something that was was made possible by the ability of SelectSim to easily introduce new covariates in the model (as explained in detail in the new Methods section, here we had to run SelectSim across individual samples rather than patients/donors, and we used donors as covariate). The results show that putative oncogenic mutations in normal

tissues often exhibit significant mutual exclusivity, but they never exhibit co-occurrence patterns. Even more, gene pairs that are mutated in a co-occurrent manner in tumor samples, were found significantly mutually exclusive in normal tissues. These results (which are discussed at greater length in the new manuscript and in response to a direct request from reviewer 2) indicate that whereas individual oncogenic mutations can be observed in normal tissues, their significant co-occurrence is primarily a cancer-associated feature.

Overall, we believe that these results well highlight the novelty of our study and its relevance to understand tumorigenesis and tumor progression, and we hope that we can convince this reviewer that this manuscript deserves to be published in Nature Genetics.

Reviewer #2 (Remarks to the Author):

In the manuscript "Evolving patterns of co-mutations from tumor initiation to metastatic progression" Iyer et al. present a new approach to analyze the patterns of co-mutations, i.e. pairs of genes that undergo mutation (or lack of) at a frequency that significantly deviates from what would be expected assuming independent mutational propensity and selection effects across genes.

The study of co-mutations is essential for better understanding the somatic evolutionary trajectories towards tumorigenesis and metastasis, with a clear potential to discover new cancer vulnerabilities and prognostic biomarkers, thereby improving therapy in the clinic. Furthermore, the evidence provided by recent sequencing studies of healthy tissues, revealing not just the presence of cancer mutations but also the emergence of not yet well understood clonal dynamics driven by those mutations, demands more nuanced genomic characterizations to understand the effects of these variants in the context of somatic evolution in normal tissues as well as their role in the progression towards premalignancy and cancer. In this regard, the goal of the paper is central to cancer genomics.

The manuscript presents SelectSim, a new method to identify co-mutations from catalogs of mutations obtained from cohorts of sequenced samples, which the authors put to use to characterize co-mutation patterns in tumors, differences between primary and metastatic tumors, whether these differences bear prognostic value as well as differences between the co-mutation patterns arising in normal urothelium and bladder cancer. To address these questions the authors make use of whole-exome and panel sequencing data in tumors (MSK, DFCI) and whole-exome sequencing of laser microdissected samples from morphologically normal urothelium.

The study is well conducted, providing a sound rationale and adequate controls. The study with tumors, both the atlas and primary vs metastasis, is robust and underscores the value of massive collection, sequencing and annotation of tumor samples in the clinic. Although it hints towards intriguing leads of tumor initiation patterns, the sequencing study with the MNU dataset is not conclusive. The manuscript is well-written and the analyses and results are described clearly.

However, there are a few important aspects of the study that would require further clarification or justification.

We thank the reviewer for the overall positive assessment of our work and their constructive suggestions, which we believe helped us improve our manuscript. While discussed in more detail in response to their specific comments, we'd like here to summarize the major new analyses introduced in this revised manuscript:

- In response to various comments from the reviewers, we more thoroughly assessed our method by testing multiple sampling procedures (i.e., drawing from distinct distributions) and designing a new model to generate artificial datasets where we predefined evolutionary dependencies among gene pairs such that a mutation in one of the two genes, lead to increase or decrease the probability of mutating the second one. These dependencies ultimately lead to the emergence of "true" co-mutation patterns. This artificial ground truth was used to assess the performances of multiple tools and demonstrate the superior performance of SelectSim.

- We comprehensively assessed the influence of our variant annotations (based on OncoKB), testing the effect of introducing variable percentages of putative neutral mutations in our data, while preserving gene alteration frequencies, and comparing OncoKB annotations with unsupervised strategies, such dNdS and AlphaMissense.

- Lastly, we completely updated our analysis of co-mutations in normal tissues, including now 5 new datasets and executing SelectSim with a different setting, i.e. using individual samples, rather than patients or donors, as units of analysis and donors as covariates. Across all datasets, these analyses led to one key finding: *whereas individual oncogenic mutations can be observed in normal tissues, significantly co-occurrent oncogenic variants are primarily cancer-associated features*.

Below, we respond in more detail to all the reviewer's comments and concerns, which we have now entirely addressed.

(PS: The reviewer may note a few changes in the reported numbers of co-mutations in the various analyses, with respect to the original figures. Indeed, we identified a small bug in the code that led us to re-run all the analyses. The results are entirely consistent with what was previously reported but a few numbers have now changed. All figures and code repositories have been updated accordingly.)

Main comments:

The study asks whether the readout SelectSim is mainly determined by selection and devises an analysis using GAMs based on a large set of driver genes with putative oncogenic vs synonymous mutations. How different are the respective GAMs in terms of sparsity or total counts? How much can this difference affect the comparison? In general, how does sparsity or total count determine

the readout of SelectSim? If true, would it then make sense to downsample so that the levels of sparsity are comparable when comparing SelectSim readouts?

The reviewer is correct, sparsity indeed affects the ability of our and other tools to detect significant co-mutation patterns. We should note however that downsampling is not necessarily the solution, indeed even if synonymous mutations do not necessarily occur at the same frequency of non-synonymous ones, that doesn't mean that they are scarcer. In our original analysis, 55% of the genes had more synonymous mutations than putative oncogenic ones, however a small set of frequently mutated cancer genes had significantly more oncogenic mutations than synonymous ones (**Figure R1a**).

To assess the effect of introducing neutral mutations while preserving gene mutation frequencies, we introduced a new analysis beside those originally presented. Specifically, for each gene we considered a progressively increasing (decreasing) percentage of neutral (oncogenic) mutations, in such a way that the total number of mutations remained the same. For example, at first, we ran SelectSim retaining for each gene only oncogenic mutations (i.e. *n* oncogenic mutations). Next, for each gene, we substituted K% of oncogenic mutations with the same number of synonymous variants or missense variants classified as neutral (for this analysis, we excluded all truncating mutations) and repeated the SelectSim analysis. Even in this setting, it was not possible to preserve the mutation frequencies of a small subset of oncogenes and tumor suppressors for all values of K. In particular, 9 genes exhibited a high-frequency of oncogenic mutations (HF genes) and not enough synonymous of putative neutral missense mutations for large values of K (**Figure R1b**). Nevertheless, we found that introducing 20% neutral variants allowed to largely preserve alteration frequencies for all genes and was already sufficient to reduce the number of significant co-mutations by 40% (**Figure R1c**). Importantly, even co-mutation patterns that involved only one or none of the HF genes reduced and eventually disappeared with increasing percentages of neutral variants (**Figure R1c**). These results now appear in the new **Suppl. Figure 2A**. In addition, we repeated the analysis for "randomly" sampled genes using again only missense variants and selecting a subset of 172 genes with matching mutation frequencies (±5%). Also in this case, several significant co-mutations were detected for cancer-associated genes but not for the randomly sampled ones (see new **Figure 1E**).

Overall, while perfectly matching gene alteration frequencies between oncogenic and neutral mutation is not always feasible, we show that already relatively small percentages of neutral variants are sufficient to reduce co-mutation patterns as nicely captured by SelectSim, further highlighting the ability of our method to detect co-mutations emerging as a result of evolutionary dependencies.

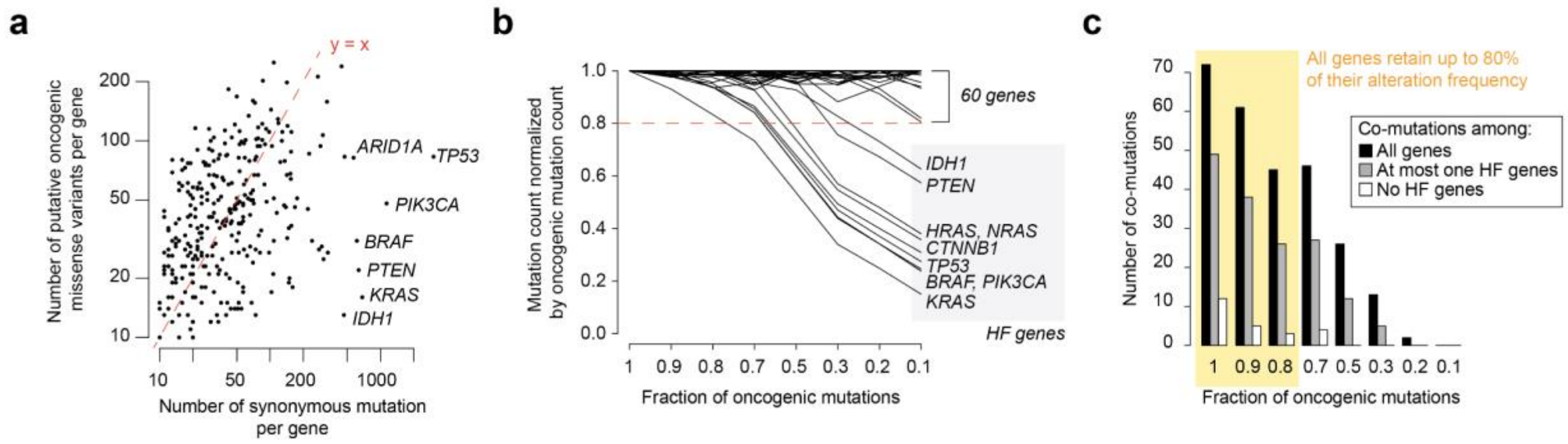

Figure R1: a) Number of synonymous vs putative oncogenic mutations per gene in the TCGA cohort for the subset of cancer associated genes used in this study. Data reflect the analysis shown in Figure 1D. **b)** Total number of mutation per gene (y-axis) across datasets that were built by mixing different k% of putative oncogenic mutations (x-axis) and (100-k)% of synonymous or putative neutral missense mutations, when available, or maximum possible number, when not enough were available. For 9 genes (labeled) not enough synonymous or putative neutral missense mutations were found to maintain the total number of mutations equal to at least 80% of the total number of oncogenic mutations (here used as reference to normalize the total mutation count on the y-axis). **c)** Number of significant co-mutations detected by SelectSim in the different datasets among all genes (black bars), among at most one HF gene (i.e., co-mutations exclusively involving HF genes were excluded - gray bars), and among non-HF genes (white bars). The yellow box comprises the datasets where all genes had a total mutation count greater than 80% of the total oncogenic mutation count.

In applying the filter of potentially oncogenic mutations across the chosen panel of genes, how many putative driver mutations does each sample have?

To respond to the direct question raised above by the reviewer, we compared the total number of mutations per sample and the total number of mutations per sample that were retained upon our filtering (truncating + OncoKB-filtered missense mutations). As expected, the total number of retained mutations per sample is significantly lower than the total number of observed mutations (**Figure R2a**), with 85% of the samples having more than 1 putative driver mutations and 5% having more than 40 (mean = 12, median = 5) (**Figure R2b**).

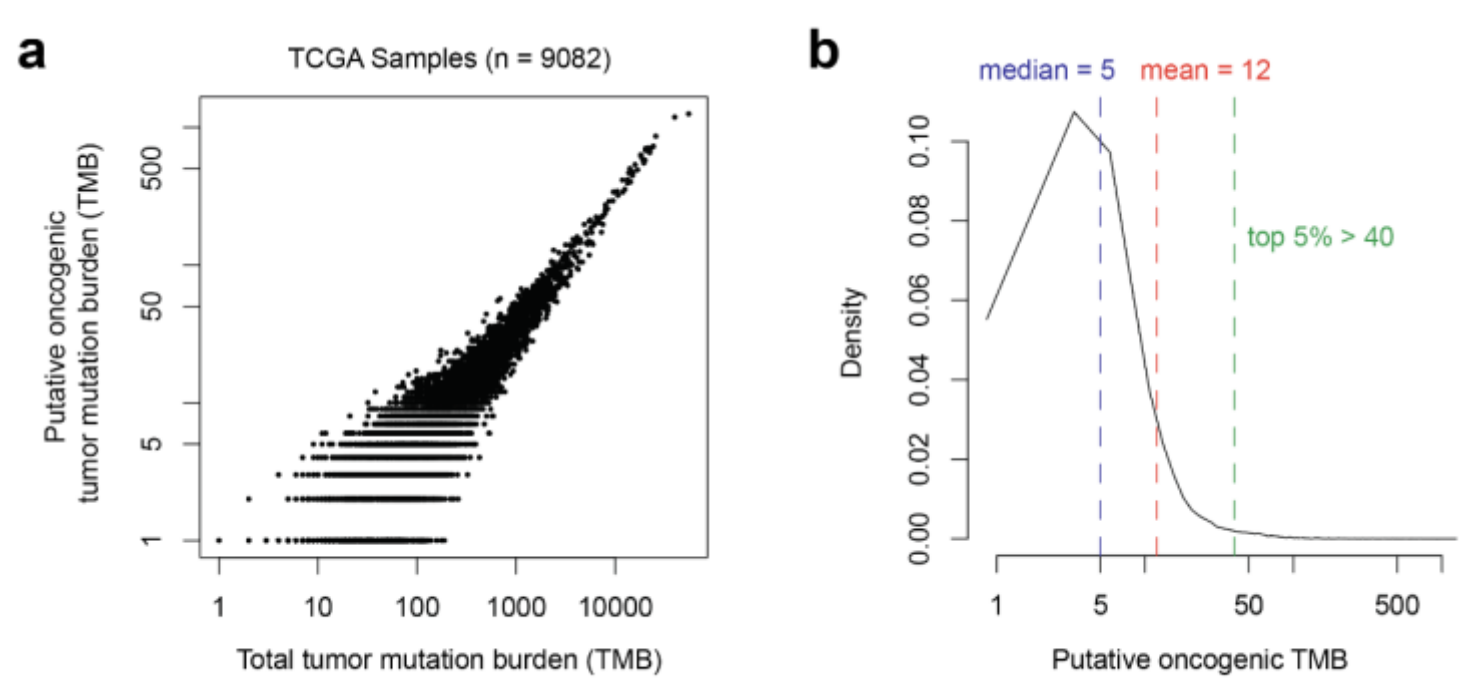

Figure R2: a) Total number of mutations per sample (a.k.a. tumor mutation burden or TMB) (x-axis), vs total number of mutations per sample upon retaining only putative oncogenic variants (y-axis). **b)** Density distribution of the TMB computed retaining only putative oncogenic variants. Median (blue), mean (red), and top 5-percentile (green) are shown.

We would like to add that here and in subsequent comments, the reviewer is concerned with the variant annotations used in this study. Indeed, the classification of a somatic mutation as putative oncogenic or neutral is still imperfect and it invariably affects the results of our analyses (as also shown above). This is particularly challenging for single amino-acid substitution (missense mutations), which are the most commonly observed type of mutations and largely associated with genetic instability and tumor mutation rates. We wish however to highlight that, unless otherwise specified, in this study we have filtered missense mutations based on variant annotations reported in OncoKB, which integrates manual curation from medical and scientific professionals, literature curation, and clinical trial evidence. To the best of our knowledge, OncoKB is among the most reliable sources for variant annotations and it is currently adopted in several molecular tumor boards (OncoKB was also granted recognition by the Food and Drug Administration - FDA).

How much of a problem can it be to annotate as potentially oncogenic mutations found in genes with no known driver association in the tumor type of the sample? Would it be a good idea to mask those counts?

If we understand correctly, the reviewer is alluding to the possibility of specific variants that have been classified as oncogenic in one tumor type, but not in another. This is an interesting point, but this analysis is complex to perform for various reasons. First, such evidence is rare and, in most cases, if a specific variant is oncogenic only in specific tumor types, it is rarely found in others and thus should not affect the co-mutation analysis. Second, even if a variant that is annotated as oncogenic in one tumor type, it is found in another that lacks such annotation, we would argue that this variant is still likely to be functional and should be included in the analysis. For example, BRAF V600K is annotated as Level 1 oncogenic mutation in OncoKB only for Melanoma. Yet this mutation is recurrent also in lung and thyroid cancer, it is known to activate BRAF and hence it is likely to be oncogenic also in these tumor types. In these cases, lack of annotation for a specific tumor type may more likely reflect lack of information rather than evidence of that variant not being functional. Finally, retaining only variants that were annotated as oncogenic specifically in the tumor type of a given sample is likely to increase even more the sparsity of our dataset, significantly reducing our statistical power. Overall, as also supported by the new analyses discussed before, we believe that our variant curation provides a good trade-off between accurate selection of oncogenic variants and a reasonable combination of strongly validated and inferred functional evidence.

How does the counts of putative driver mutations compare with the expected number of driver mutations per sample according to prior knowledge about the number of driver mutations per sample? This can be a simple though compelling sanity check.

I think this comment stems from some misunderstanding concerning our putative driver mutation filtering, we apologize for this. As discussed in response to the previous comments, we used OncoKB to define “putative driver mutations”; we did not develop a new approach for variant annotations. As such, the number of putative drivers that we obtained across the many different datasets is by definition what is “expected according to prior knowledge”, being OncoKB a curated

summary of such prior knowledge (i.e., data curation from scientists and medical experts, literature curation, and clinical trial evidence).

Expanding on this, I wonder whether the synonymous mutation data per gene can be exploited to assess the amount of positive selection, e.g. via dN/dS type of approach, to provide another level of assessment of whether the total number of putative driver mutations per gene might be inflated, hence corrected.

We understand the concern from this reviewer and to address this point we used two alternative unbiased approaches to define putative driver variants: dNdS (using the specific *sitednds* function since originally dNdS was developed to infer genes or domains mutated more frequently than expected, rather than individual residues) and AlphaMissense, an alternative unbiased variant annotator that was recently developed leveraging deep-learning models (Cheng et al Science, 2023). As the reviewer pointed out, dNdS exploits synonymous mutations to infer positive selection, whereas AlphaMissense unbiasedly predicts variant pathogenicity based on predicted protein structural changes induced by a given mutation, sequence conservation, and public annotations. Sitednds predicted a smaller number of oncogenic missense mutations than those annotated in OncoKB, nevertheless the total mutation counts per sample were highly correlated exhibiting only small differences (**Figure R3a**). Conversely, AlphaMissense predicted a higher number of pathogenic variants per sample compared to OncoKB annotations even when only variants with AlphaMissense scores > 0.9 were retained (**Figure R3b**). When we ran SelectSim with variants annotated based on these tools, we found overall good concordance, with 73% shared significant co-mutations between results obtained with OncoKB and dNdS annotations, and 62% between results obtained with OncoKB and AlphaMissense annotations. Overall, while the use of these unbiased approaches may allow to include additional variants, the results are largely consistent, and we feel that OncoKB annotations are better supported by clinical and biological evidence being the only annotation dataset currently used in the clinic to call oncogenic variants.

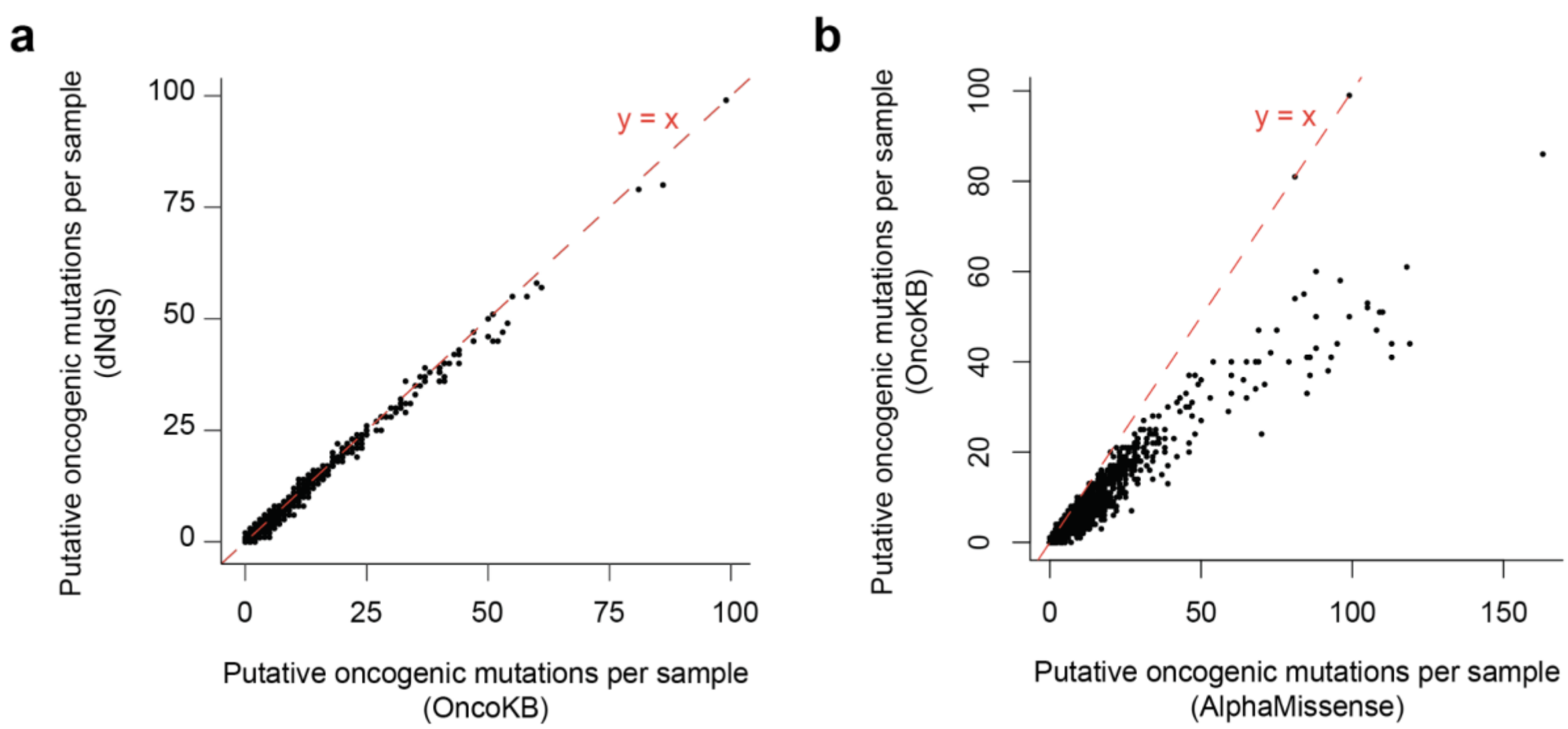

Figure R3: Total number of putative oncogenic missense mutations per sample based on OncoKB and either dNdS (**a**) or AlphaMissense (**b**) annotations.

If the number of putative drivers accounted for is too high, that would mean that neutral or passenger mutations leak into the model. How much then may the neutral mutation heterogeneity confound the analysis of co-mutations? The method's expectation is made proportional across genes and samples, but this proportionality might not hold neutral mutation wise.

The reviewer is correct, if neutral mutations leak into the model, co-mutation patterns will be affected. As already discussed in response to a previous comment, we were able to test this hypothesis and demonstrate that increasing percentages of putative neutral mutations introduced in our input data to substitute a corresponding number of putative oncogenic variants lead to reduced and eventually disappearing co-mutation patterns. This analysis was introduced in response to a previous comment from this reviewer along with a summary of its result in **Figure R1, R4** and the new **Suppl. Figure 2A**. Here, we just briefly summarize the approach and provide an additional visualization of the results (also in **Suppl. Figure 2A**): starting from the TCGA dataset where only putative oncogenic missense mutations were considered, we generated new input datasets where K% of oncogenic mutations were substituted with K% (or max detected) of neutral variants (including synonymous and putative neutral missense mutations). SelectSim was run on all datasets separately and nicely it showed that the number of significant co-mutations decreases as the fraction of neutral variants increases, till none are found significant (**Figure R4**).

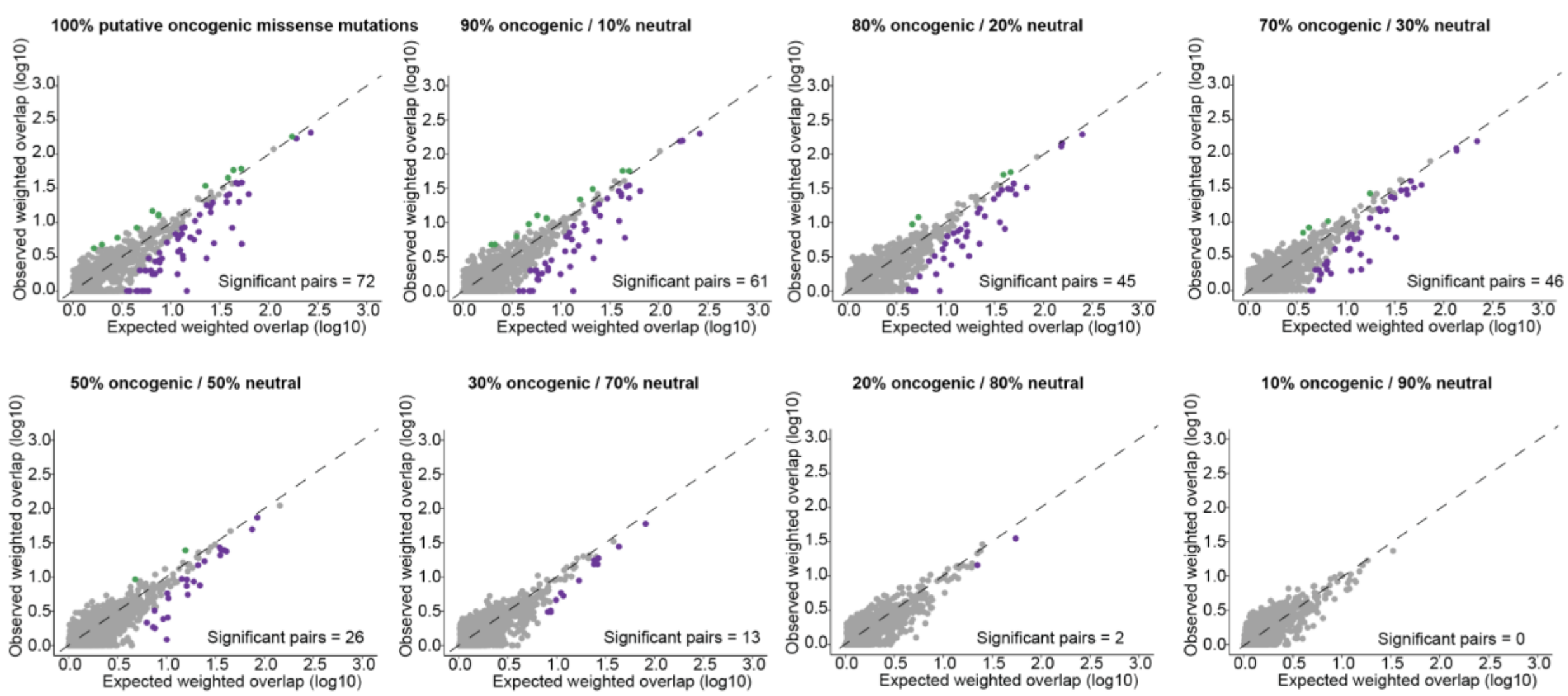

Figure R4: Observed weighted co-mutation (x-axis) vs Expected weighted co-mutation (y-axis) across 8 datasets comprising different proportions of oncogenic and neutral mutations for the same set of genes.

To what extent the results could be affected by the fact that there are different driver mechanisms implying that some genes require a single hit while others require a double hit, with one of the hits not necessarily being a mutation, e.g. deletion? Should (and how) the observed frequencies at each driver gene be adjusted for the expected mechanism of action of the driver gene?

This is an interesting question, especially for what concerns tumor suppressors, which often require two hits to be fully inactivated. To address this question, we retrieved all significant co-occurrent mutation patterns in the lung adenocarcinoma and pan-cancer datasets from TCGA, selecting those that involved at least one tumor suppressor. Within these patterns, we computed the fraction of samples that exhibited 2 hits for the tumor suppressor gene(s), either among all samples where at least one of the 2 genes were mutated (sample union), or only among the samples where both genes were co-mutated (sample intersection). *The rationale was to assess whether the "double-vs-single hit fraction" among co-mutated samples was higher or lower than among all mutated samples*, indicating that a bias exists for co-mutations to appear preferentially between single or double hits.

For this analysis, we classified a sample as "double-hit" for a given gene, if that sample harbored either more than one mutation, or one mutation and a copy number loss (-1, -2 GISTIC calls) on that gene. Otherwise, a sample is called "single-hit" for that gene. Overall, this comparison showed a high correlation between double-vs-single hit fractions among all mutated samples and among co-mutated samples, suggesting that co-mutation patterns are not biased for either single or double hit samples (**Figure R5**). Notably, the only points that deviated from the diagonal were co-mutations involving tumor suppressors mutated at high frequency, especially *TP53* (**Figure R5**). However, these deviations can be explained by the fact that the mutation frequency of *TP53* is up-to-2 orders of magnitude higher than those of most other genes, as such the double-to-single hit sample fractions for samples with at least one mutation is dominated by *TP53* mutant samples and remain fairly constant, conversely the double-to-single hit sample ratio of co-mutated samples exhibit more variability consistent with more variable co-mutation frequencies. Overall, these results indicate that co-mutations are not enriched in double or single hit samples and discriminating among double and single hits in our analyses is unlikely to increase the signal-to-noise ratio, but it would only increase data sparsity and reduce statistical power.

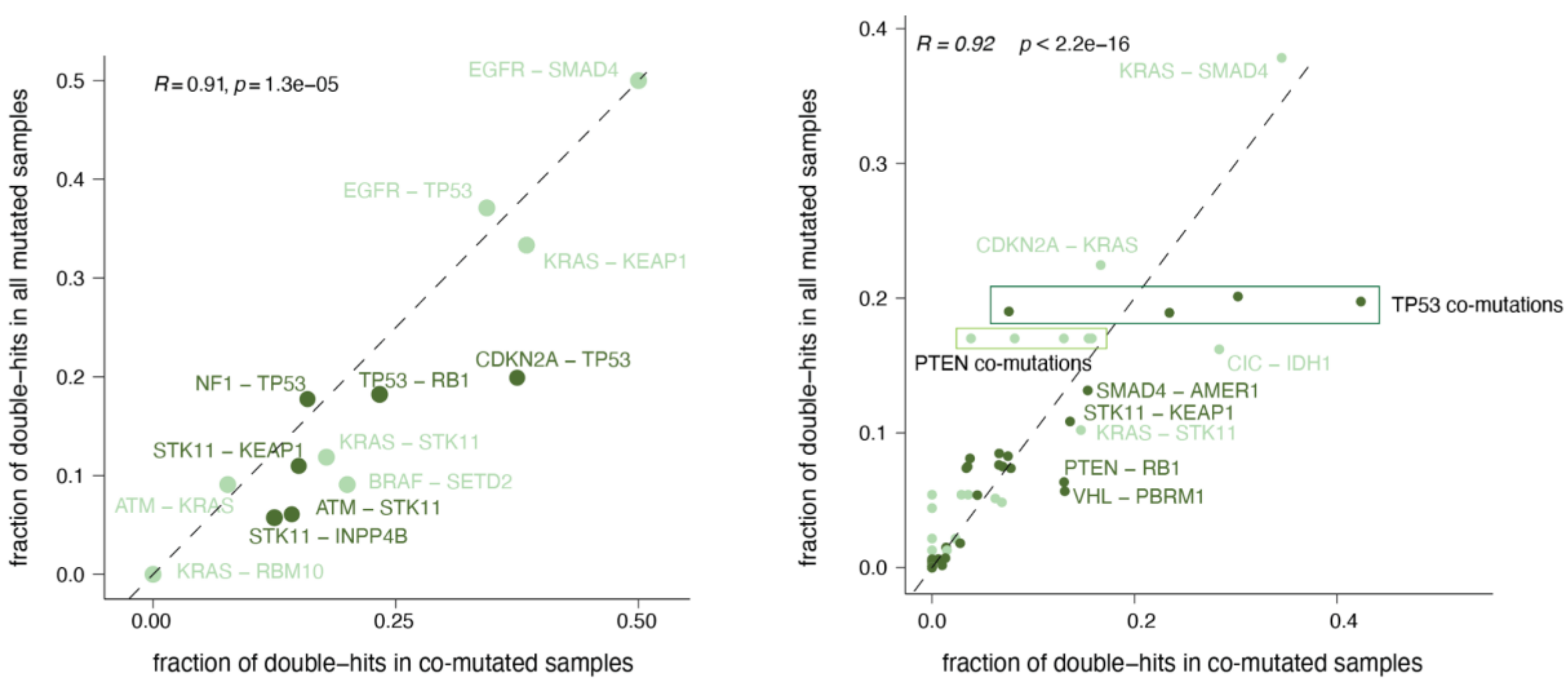

Figure R5: Fraction of samples exhibiting a double hit event for tumor suppressors among co-mutated samples (x-axis) and among all mutated samples (y-axis). Each dot is a significant co-occurrently mutated pair of genes in the TCGA lung adenocarcinoma dataset (left) or in the TCGA pan-cancer dataset (right).

Spearman's correlation coefficient and associated p-value are reported. Dark green dots refer to co-mutations between 2 tumor suppressors, and light green to co-mutations between 1 tumor suppressor and another gene.

The fact that large genes such as TTN and MUC16 show up as significant co-mutations in the randomly generated gene set, highlights the concerns made in my two previous comments: for one, whether the putative oncogenicity filter allows too many mutations, thereby leaking neutral mutation counts in the GAMs; for second, the fact that double hits representing a single oncogenic event may make a difference in the SelectSim readout.

We understand the reviewer's concern, but we think the issue with the few false positive hits involving large genes such as *TTN* and *MUC16* is a different one. First, as shown in the comparison with AlphaMissense and dNdS, our filtering is quite stringent and the number of retained oncogenic mutations is unlikely to be inflated. Second, as shown in the analysis with different admixing of putative oncogenic and neutral mutations, leaking of neutral mutations is more likely to reduce co-mutation patterns (**Figure R4**) than to generate new (false) ones. Third, the problem with these very large genes is that they have a very high number of missense mutations, which are enriched in tumors with a very high tumor mutation burden.

In particular, these genes typically harbor multiple mutations per sample, even after retaining only missense mutations recurrently affecting the same residue. As a consequence, their alteration frequency is underestimated by our binary alteration matrices, which simply define a sample as wild-type or mutated for a given gene, independently of how many mutations are present for that gene. Hence, the null model generates fewer co-mutations than observed, which eventually score as significantly co-occurrent. Cancer genes exhibiting such a high number of mutations per samples upon OncoKB filtering are however never observed, and very large genes such *TTN* and *MUC16* are never included into the list of cancer genes that we analyze with SelectSim. For these reasons, the "single oncogenic event" approximation (i.e., binary alteration matrix), while imperfect, is reasonable for the scope of our analyses and the vast majority of genes.

In-silico simulation of GAMs (l.806-807). I found this sampling method somewhat counterintuitive. For example, how would that differ from drawing from a Poisson or negative binomial distribution, then binarizing the integer output? How do the dispersions compare?

We apologize for the lack of clarity. We have now more extensively explained the statistics behind our procedure and compared it against other sampling approaches, as also suggested by another reviewer.

Briefly, the method that we use is a modification of rejection-acceptance sampling that assumes a Bernoulli distribution for each entry of the GAM (genome alteration matrix) with parameter p equal to the corresponding entry of the template matrix (i.e. the matrix of expected mutation counts resulting from the outer product of the vector of gene mutation counts and the vector of normalized tumor mutation burdens - see Methods). Specifically, our "proposal distribution" is a trivial distribution with only one value 1 with probability 1, and the "desired distribution" is the Bernoulli distribution in the template matrix. Subtraction with the uniformly distributed sample is

part of the rejection approach according to which one should keep or reject the “fictitiously” drawn sample equal to 1 depending on whether its probability mass (equal to Bernoulli p) is higher or lower, respectively, than the uniform number. In addition, we modified this procedure to exactly preserve gene mutation frequencies. To do so, among all matrix entries we don’t keep those higher than their respective uniformly drawn numbers, but we keep those higher than their respective uniformly drawn numbers minus or plus an epsilon value selected to ensure that the row sums of the simulated alteration matrices are identical to the observed ones (*row sum correction*).

To fully address the reviewer’s concern, we benchmarked distinct sampling procedures by generating 1000 GAMs using: 1) our rejection-acceptance approach with and 2) without row sum correction, 3) direct Bernoulli sampling, 4) negative binomial sampling, and 5) Poisson sampling with subsequent binarization. After applying SelectSim using different sets of GAMs, we observed that results obtained with the different sampling approaches exhibited a good overlap with 43% of significant co-mutations detected using more than one sampling approach and 30% (22 out of 74 in total) of them detected using all 5 approaches (**Figure R6a**). The main differences stemmed from: **1)** applying row sum correction, which recovered 23 additional co-mutations, indeed a major limitation of the other sampling approaches is that of inherently generating integers/binary values and not exactly preserving gene alteration frequencies (**Figure R6b**); and **2)** from the results obtained with Poisson sampling, which most underestimate gene mutation frequencies, especially among recurrently mutated genes (*KRAS* and *TP53*), leading to a higher number of likely false positive hits.

Finally, we calculated log-likelihoods of all generated GAMs according to the template matrix and their assumed underlying distributions. Our rejection approach with row sum correction obtained the lowest values, i.e. GAMs generated by our approach are the most likely instances of the null model (**Figure R6c**).

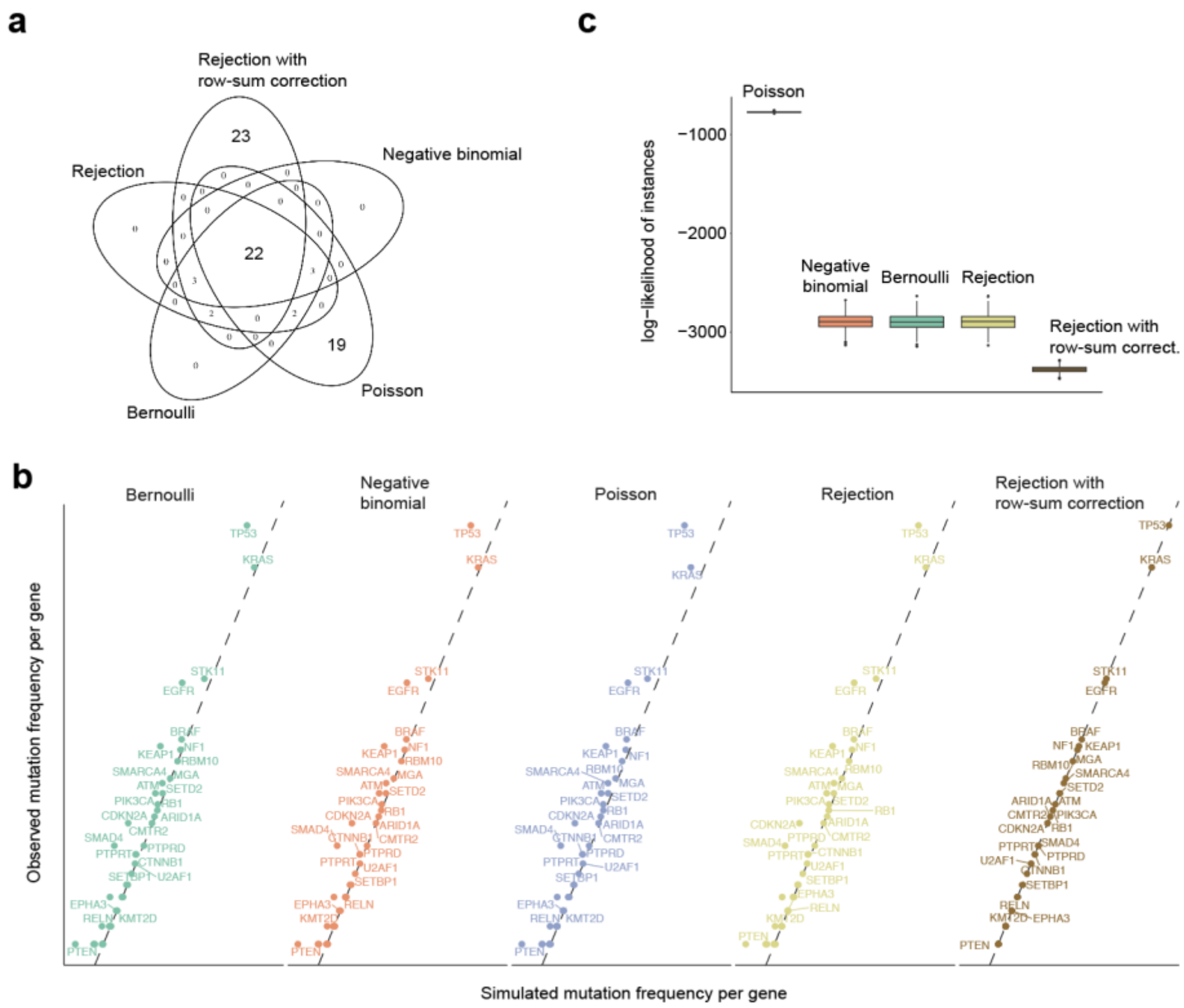

Figure R6: a) Intersection of significant co-mutation detected using 5 distinct sampling approaches to simulate gene alteration matrices. b) Simulated (x-axis) vs. observed (y-axis) gene mutation frequency based on 5 distinct sampling approaches. c) Log-likelihood values of GAMs generated using 5 distinct sampling approaches.

Assuming that the mutation catalogs from MSK and DFCI contain germline mutations, the co-mutation analysis involving this dataset implies a mix of cancer predisposition variants and somatic mutations. I wonder whether any significant co-mutations found upon this dataset are enriched in germline mutations, pointing towards early driver events in the multistage tumorigenic transformation process.

This would be indeed an interesting analysis to do, but unfortunately the MSK and DFCI datasets were filtered to eliminate putative germline variants before their release, as outlined in the AACR-GENIE public data guide. (Details are discussed in the section "Germline Filter" at page 6).

Although rooted in intuition, the conceptualization of the term "trajectory" is vague. The manuscript should provide some sort of formal or operational definition.

We agree that the term was not properly defined in our context. We amended the text to avoid confusion stating: "In several cases, co-mutated gene sets highlighted distinct evolutionary trajectories of the disease, here defined as a set of co-occurring mutations that are likely to have emerged and been co-selected during the evolution of the disease".

The prognostic value of co-mutations as biomarkers of metastasis is original and convincing. I

wonder whether these prognostic effects result via an interaction with the type of treatment of the primary tumor, or else they are predictors regardless of the treatment.

We thank the reviewer for the positive assessment of these analyses. To test to what extent treatment predicts metastatic progression or could explain our previous predictions, we revised the ElasticNet analyses previously presented in **Figure 3** including treatment as covariates. Treatment data was retrieved from the MSK-CHORD dataset. While the new figure panels now reflect results including treatment as covariate, we found that the results were largely coincident, and the elastic net coefficients associated with co-mutations in the new analysis were very strongly correlated with those of the previous analysis (**Figure R7a**). Moreover, the range of values of the elastic net coefficients obtained by treatment variables was narrower than the one exhibited by co-mutation coefficients, indicating lower predictive values (**Figure R7b**). Overall, these results indicate that the prognostic effects that we identified in our analyses are largely independent of the type of treatment of the primary tumor. The updated results are in **Figure 3**.

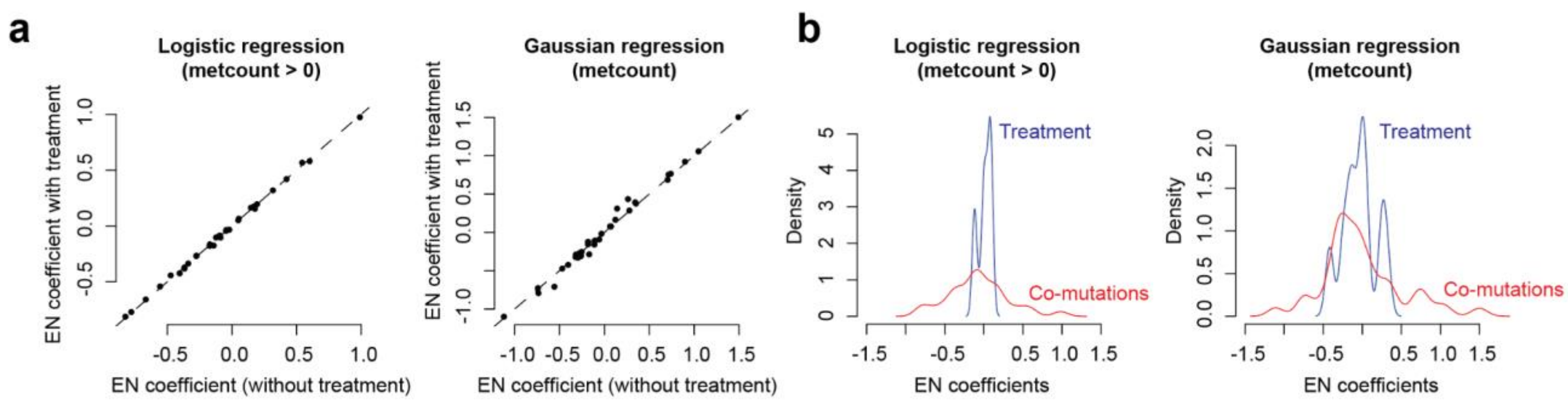

Figure R7: **a)** Scatterplots of EN coefficient obtained by co-mutations when excluding (x-axis) or including (y-axis) treatment as a covariate. Results are shown for both logistic (left) and gaussian (right) regression models. **b)** Density distributions of EN coefficient obtained by co-mutations (red) or treatment (blue) for logistic (left) and gaussian (right) regression models.

Why did authors not include the sequencing study of normal urothelial cells by Lawson et al? Would the results yielded by the MNU analysis reproduce? This would provide solid support for the main takes of this otherwise interesting part of the study. Although one of the justifications for sequencing LCMs is to call somatic mutations from oligoclonal samples, there is still a chance for low VAF clones to escape detection. Claiming that co-mutations are not present may be oblivious of the fact that the co-mutation might be there albeit in small clones. Regarding the comparison with bladder tumors, it might be a good idea, in order to understand the tumor promoting (or protecting) role of co-mutations and their involvement in early vs late tumor evolutionary trajectories, to stratify the bladder tumors into non-muscle-invasive and muscle-invasive subtypes.

Following this and an earlier comment from the reviewer, we ended up completely revising our analysis of co-mutations in normal tissues, which indeed was preliminary and not accounting for several large, published datasets, like the Lawson et al. study.

These analyses however required a change in setting. First of all, most studies of somatic mutagenesis in normal tissues have made use of multiple samples/biopsies for a limited number of donors, as opposed to cancer genomics studies where 1 or a few samples are available for a large number of patients (that was the initial reason for choosing the Li et al study, which provided multiple donors with one or few samples per donor). In addition, the mutation frequency of cancer genes in these samples is typically considerably lower than in cancer, increasing data sparsity. To cope with these challenges, we decided to test co-mutations directly among different samples, rather than donors/patients, and use donors as covariates (i.e., we estimated gene mutation frequencies and normalized sample mutation burden within each donor, similarly to what we did for tumor subtypes in the TCGA/MSK/DFCI datasets – as a result we ended up excluding the Li et al study that we originally analyzed, as it cannot be analyzed in this setting).

In addition, to compare co-mutation patterns between normal tissues and tumors from the TCGA/MSK/DFCI cohorts that originated from the same tissue types, we retained somatic variants for 396 cancer associated genes and adopted the same variant filtering that we used in the tumor datasets (unless otherwise specified). As a result, among 8 different studies that we examined, we eventually ran SelectSim on 5 of them, where enough samples/donors and mutations were available. These included the Lawson study suggested by the reviewer, from which we focused on 926 normal urothelium samples from 15 donors, the Abby-2023 and Martincorena-2018 studies from which we retained in total 918 normal esophagus epithelial samples from 12 donors, and the Fowler-2021 and Martincorena-2015 studies from which we retained in total 2580 normal skin samples from 17 donors.

We ran SelectSim on the 3 tissue types separately, integrating datasets analyzing the same tissue type, and in all of them we found multiple significantly mutually exclusive mutations, but no significant co-occurrence (except for one marginally significant hit that came from only one donor). Such results were in stark contrast with results in melanoma, esophageal, and bladder cancer, where multiple significant co-occurrent mutations were found.
In particular, normal urothelium samples exhibited putative oncogenic mutations at genes that are also frequently altered in bladder cancer. However, none of the significantly co-occurrent mutations in bladder cancer were found co-occurring in normal samples. Even more, gene pairs that were among the most significantly concurrently mutated in bladder cancer (such as *KDM6A-STAG2* and *KMT2C-KMT2D*) were found to be mutated in a significant mutually exclusive manner in normal urothelium samples (see new **Figure 4** in the manuscript). *Hence, co-occurrence of oncogenic mutations seems primarily a cancer-associated feature*.

Interestingly, the normal urothelium dataset included normal samples derived from cancer patients, from whom matching carcinoma in situ (CIS) and invasive tumor samples were available. Here, we observed that normal urothelium samples derived from cancer patients shared more mutations than samples from healthy donors and exhibited co-occurring variants that were often shared among multiple biopsies, suggestive of clonal expansion of co-mutated clones. Nevertheless, these co-mutations were not found in matching CIS and tumor samples, which instead exhibited bladder cancer-associated co-occurrent mutations that were not found in normal samples, such as co-occurring mutations at *KMT2D* and *KMT2C* or at *TP53* and *RB1*. Although

preliminary, these results warrant further investigation to explore, for example, whether cell states in normal tissues exist that allow to tolerate co-mutations, promote their emergence, and eventually increase the risk of acquiring those required for tumorigenesis (we discuss these points in the Discussion section).

While additional datasets will be needed to corroborate these findings, these results indicate that while individual oncogenic mutations can be observed in normal tissues, significantly co-occurrent oncogenic variants are primarily observed in cancer, consistent with the hypothesis that specific combinations of variants are required for tumorigenesis.

Minor comments:

How is the random gene generation (featured in Figure 1D, third panel) done?

This analysis has now been updated to better preserve gene mutation frequencies. Specifically, we now focused exclusively on missense mutations, and we randomly sampled 172 genes whose number of recurrent missense mutations (i.e., single amino-acid substitution occurring in the same position in at least 2 samples) matched the number of putative oncogenic missense mutations of 172 cancer-associated genes included in our catalog. (In the original, analysis we sampled these genes following a broad categorization of frequency intervals, which however sometimes led to large differences among gene mutation frequencies.) We found numerous significant co-mutations among cancer genes, and only 2 among randomly sampled genes, again involving very large genes (*TTN-MUC16* and *TTN-CSMD3*). We discuss the source of the problem in the manuscript, explaining why these are likely false positives.

Figure 2F, what does a connection between two gene sets mean?

We have now clarified in the text and figure label that a connection between two gene sets means that each gene from one set is mutated concurrently (green edge) or exclusively (purple edge) with each other gene in the other set.

Figure 4E are the donor labels right?

Figure 4 has been completely updated to reflect the new analyses.

I would suggest to use another notation for the outer product, rather than “x”

We substituted the letter “x” with the symbol for the outer product, ⊗, where needed, otherwise, we indicated the product between vectors or matrices with the × symbol rather than the letter “x” as also suggested by reviewer 3. E.g.:

$$E = n_t \boldsymbol{f}(g) \otimes \boldsymbol{\mu} = n_t \boldsymbol{f}(g) \times \boldsymbol{\mu}^T$$

l.803-804: what does (E)=1 mean?

We apologize for the typo, the line meant to read: “max(E) = 1”. We amended the text.

l.820: how is the m_i value specified? this sentence is very confusing, please clarify

We apologize for the lack of clarity, and we have now amended the text to better explain this step. Basically, the introduction of m_i is required for the row sum correction of our rejection-acceptance sampling procedure, which we discussed in more detail in response to previous comments from this reviewer. The classical rejection-acceptance sampling procedure would have m_i = 0 for all rows, i.e., one should accept the “fictitiously” drawn sample equal to 1 if its probability mass (equal to Bernoulli p) is higher than the uniformly drawn number, which corresponds to the subtraction with the uniformly distributed sample being greater than 0. By asking this subtraction to be greater than m_i (instead of zero), we can accept more or less “fictitiously” drawn samples, such that their total number is equal to the number of original mutations of the gene in row “i”. This is a rather simple strategy to preserve gene alteration frequencies, but it turned out to be an extremely effective one as shown in **Figure R6**.

Why 80% subsampling in the metastasis vs primary analysis?

The number of metastases and primary samples differ across tumor types and larger datasets provide greater statistical power. Hence, a fair comparison required to run SelectSim on datasets of the same size. We decided to fix this size to 80% of the size of the smaller dataset (metastasis dataset) as a trade-off between maintaining the datasets as big as possible and allow for some heterogeneity across the resampled runs, to increase the robustness of the results.

Supplementary fig 2A: APC-TP53 colorectal cancer? What are the grey and black dots? non-significant vs significant?

In that case the dots were colored differently simply to facilitate the visual separation between tumor types: if dots related to one tumor type are in black, dots related to the next one will be in gray and vice versa. This figure has however been updated.

What is going on in the case of colorectal cancer? Can this apparently incoherent TP53-APC co-mutation be attributed to mixed tumor subtypes?

As mentioned above, this analysis has been updated and led now to more consistent results. Note that certain variability still exists, which is more likely related to the different datasets than distinct subtypes. Indeed, tumor subtypes (when annotated as in colorectal cancer) are always used as covariates in SelectSim. Here, however we reported results for all individual cohorts (analyzed with different gene panels in different years) within MSK, DFCI, and TCGA, and a certain degree of heterogeneity is to be expected.

Reviewer #3 (Remarks to the Author):

This paper introduces SelectSim, a method to identify pairs of somatic mutations that are either significantly co-occurring or significantly mutually exclusive. Briefly, SelectSim takes as input a binary sample x gene matrix and for each pair of mutations computes an effect size (negative for mutex, positive for co-occuring, 0 for no effect) and statistical significance. This is done by simulating alternative binary matrices from a null model derived from the data. Rather than using a permutation test or computing p-values exactly, SelectSim generates alternative binary matrices (without stringently enforcing row/column marginals). The results of real data are strong. My main criticism is that the method and underlying null model needs to be better described, and that a simulation study (with known ground truth) is warranted, especially given the heuristic nature of SelectSim.

We thank the reviewer for the overall positive assessment of our work and their constructive suggestions, which we believe helped us improve our manuscript. While discussed in more detail in response to their specific comments, we'd like here to summarize the major new analyses introduced in this revised manuscript:

- In response to various comments from both reviewers, we improved the description of the method and more thoroughly assessed SelectSim simulations by testing multiple sampling procedures (i.e., drawing from distinct distributions),

- We now introduce a new model to generate artificial datasets where we can predefine evolutionary dependencies among gene pairs such that a mutation in one of the two genes, leads to increase or decrease the probability of mutating the second one. These dependencies ultimately lead to the emergence of "true" co-mutation patterns. This artificial ground truth was used to assess the performances of multiple tools and demonstrate the superior performance of SelectSim.

- Lastly, we completely updated our analysis of co-mutations in normal tissues, including 5 new datasets and executing SelectSim with a different setting, i.e. using individual samples, rather than patients or donors, as units of analysis, and donors as covariates. Across all datasets, these analyses led to one key finding: *whereas individual oncogenic mutations can be observed in normal tissues, significantly co-occurrent oncogenic variants are primarily cancer-associated features*.

Below, we respond in more detail to all the reviewer's comments and concerns, which we have now entirely addressed.

(PS: The reviewer may note a few changes in the reported numbers of co-mutations in the various analyses, with respect to the original figures. Indeed, we identified a small bug in the code that led us to re-run all the analyses. The results are entirely consistent with what was previously reported but a few numbers have now changed. All figures and code repositories have been updated accordingly.)

1. Better description of the method

- It seems that the method is attempting to generate alternative binary matrices under the assumption of independence among genes and samples while maintaining gene mutation frequencies and tumor mutational burdens (for samples). In other words, sample binary matrices with fixed row and column marginals. It would be good to state this upfront in Methods.

The reviewer is correct in their interpretation, and we realized our description of the method was often lacking the required clarity. In response to remarks from both reviewers, we have now revised the method description in both the Results and Methods sections.

Also, it is important to discuss current approaches for sampling such constrained matrices (e.g. MCMC based on the swap/knight's tour algorithm, or sequential importance sampling.) Then, please discuss how SelectSim differs from these standard approaches.

To address this comment, we'd like to offer a clarification and present new comparisons with sequential importance sampling (SIS). First of all, the MCMC-based swap algorithm mentioned by the reviewer is indeed what is adopted in SELECT, the method that we previously developed (Mina et al. 2017) and that we included in the benchmarking comparisons in this study. SELECT adopts the MCMC swap approach as implemented in the package BiRewire (Gobbi et al 2022), which is inspired by our previous work (Ciriello et al 2012) and by the edge-swapping algorithm for graph randomization originally introduced by Ron Milo and Uri Alon (Milo et al 2002). The fundamental difference with SelectSim is that the MCMC/swap approach is a data permutation strategy that preserves the matrix marginals, conversely SelectSim employs a data simulation strategy that preserves the matrix marginals. Both approaches provide high sensitivity and specificity (*this is discussed in more detail below, when we introduce a new ground truth model*), but SelectSim was more robust to data resampling and better captured co-mutations emerging through co-selection (see updated **Figure 1** and **Suppl. Figure 1**).

Next, we implemented and tested the SIS approach suggested by the reviewer, as an alternative to generate expected matrices of alterations. At first, we used the LearnBayes package in R, but its running time proved prohibitive, growing exponentially with the number of samples and becoming unfeasible for our dataset. Using this package, we managed to compare null instances generated with SIS and our approach in a small dataset of 20 lung adenocarcinoma samples. Only 10% "outlier" instances generated with SIS had log-likelihood lower than those obtained with our approach. It should be noted that SIS by definition uses a "proposal distribution" to draw from and then accepts instances as if they were from the desired distribution. The implementation in the LearnBayes package is limited to multivariate t-distribution as the proposal. To overcome this limitation, we drew samples using our approach ("rejection-acceptance with row sum correction" see more detailed description below) as the proposal distribution, and re-sampled them according to a SIS procedure that we reimplemented. This approach did not change log-likelihood of the generated null instances and neither the results of SelectSim when applied to these sets of nulls returning the same set of co-mutations as significant.

Finally, as pointed out in various comments from both reviewers, we previously failed to properly explain our sampling strategy and compare it with other sampling strategies based on different distributions. To clarify our approach, what we are doing is using a “rejection-acceptance sampling” that assumes a Bernoulli distribution for each entry of the gene alteration matrix (GAM) with parameter *p* equal to the corresponding entry of the template matrix (i.e. the matrix of expected mutation counts resulting from the outer product of the vector of gene mutation counts and the vector of normalized tumor mutation burdens - see Methods). Specifically, our “proposal distribution” is a trivial distribution with only one value 1 with probability 1, and the “desired distribution” is the Bernoulli distribution with parameter in the template matrix. Subtraction with the uniformly distributed sample is part of the rejection approach according to which one should accept or reject the “fictitiously” drawn sample equal to 1 depending on whether its probability mass (equal to Bernoulli p) is higher or lower, respectively, than the uniform number. In addition, we modified this procedure to exactly preserve gene mutation frequencies. To do so, among all matrix entries we don’t keep those higher than their respective uniformly drawn numbers, but we keep those higher than their respective uniformly drawn numbers minus or plus a small epsilon value selected to assure that the row sums of the simulated alteration matrices are identical to the observed ones (*row sum correction*). We have now compared our rejection-acceptance sampling with multiple sampling approaches based on Bernoulli, Poisson, and Negative Binomial distributions. Results of this comparison are presented in response to a direct question from Reviewer 2 and copied here for this reviewer’s perusal.

“To fully address the reviewer’s concern, we benchmarked distinct sampling procedures by generating 1000 GAMs using: 1) our rejection-acceptance approach with and 2) without row sum correction, 3) direct Bernoulli sampling, 4) negative binomial sampling, and 5) Poisson sampling with subsequent binarization. Assumption of a different statistical distribution determines the way the template matrix is handled. After applying SelectSim using different sets of GAMs, we observed that results obtained with the different sampling approaches exhibited a good overlap with 43% of significant co-mutations detected using more than one sampling approach and 30% (22 out of 74 in total) of them detected using all 5 approaches (**Figure R6a**). The main differences stemmed from: **1)** applying row sum correction, which recovered 23 additional co-mutations, indeed a major limitation of the other sampling approaches is that of inherently generating integers/binary values and not exactly preserving gene alteration frequencies (**Figure R6b**); and **2)** from the results obtained with Poisson sampling, which most underestimate gene mutation frequencies, especially among recurrently mutated genes (*KRAS* and *TP53*), leading to a higher number of likely false positive hits.

Finally, we calculated log-likelihoods of all generated GAMs according to the template matrix and their assumed underlying distributions. Our rejection approach with row sum correction obtained the lowest values, i.e. GAMs generated by our approach are the most likely instances of the null model (**Figure R6c**).”

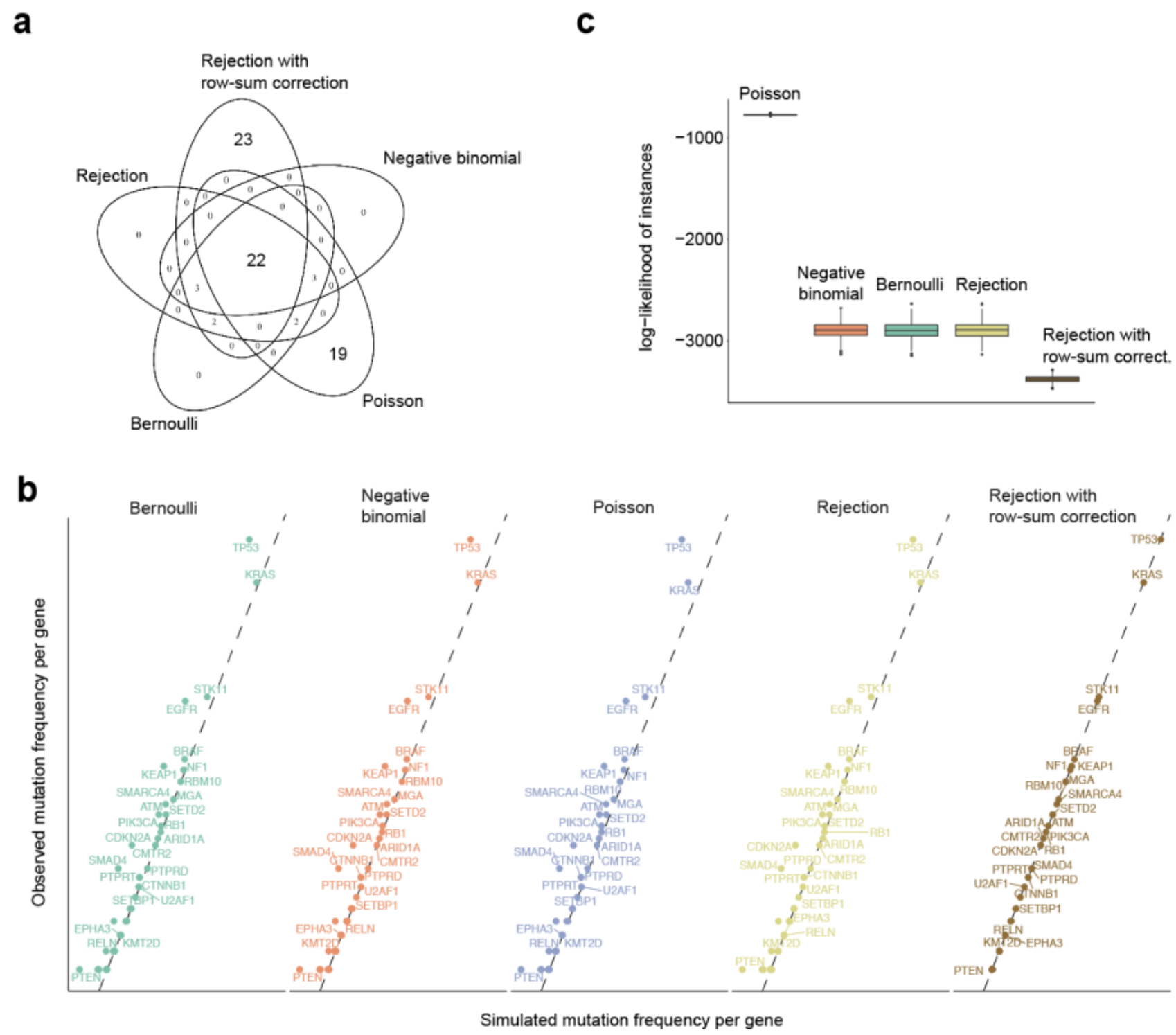

Figure R6: a) Intersection of significant co-mutation detected using 5 distinct sampling approaches to simulate gene alteration matrices. b) Simulated (x-axis) vs. observed (y-axis) gene mutation frequency based on 5 distinct sampling approaches. c) Log-likelihood values of GAMs generated using 5 distinct sampling approaches."

Overall, we think we have now well demonstrated the efficacy and efficiency of our rejection-acceptance sampling.

- Additionally, I found the mathematical description missing important definitions.

We apologize for this lack of clarity; the required details and definition are provided below and have been included in the revised Methods section.

- line 787: define TMB(t_j). Is it \sum_{g \in G} GAM(g,t_j)?

The TMB of each sample is computed as the total number of mutations across all sequenced genes for that sample. Hence, it may comprise genes and variants that were not retained in the GAM. The rationale is that the GAM reflects the set of putative oncogenic mutations in the dataset (i.e., mutation under positive selection), whereas the TMB (i.e. all mutations) is used as a proxy of the overall mutation rate in a sample. TMB values are derived directly from the corresponding Mutation Annotation File (MAF) of the dataset and are provided as a separate input file to SelectSim.

- line 798 (and also later): Use \times rather than the letter x to indicate matrix multiplication.

We substituted the letter “x” with the symbol for the outer product, ⊗, where needed, and otherwise, we indicated the product between vectors or matrices with the × symbol rather than the letter “x” as also suggested by the other reviewer. E.g.:

$$E = n_t \boldsymbol{f}(g) \otimes \boldsymbol{\mu} = n_t \boldsymbol{f}(g) \times \boldsymbol{\mu}^T$$

- line 803: "We set (E) = 1." -- What does this mean?

We apologize for this typo, the line meant to read: “max(E) = 1”. Sorry for the confusion.

- line 820: what is m_i? The current description is unclear.

We apologize for the lack of clarity, and we have now amended the text to better explain this step. Basically, the introduction of m_i is required for the row sum correction of our rejection-acceptance sampling procedure, which we discussed in more detail in response to previous comments from this reviewer. The classical rejection-acceptance sampling procedure would have m_i = 0 for all rows, i.e., one should accept the “fictitiously” drawn sample equal to 1 if its probability mass (equal to Bernoulli p) is higher than the uniform number, which corresponds to the subtraction with the uniformly distributed sample being greater than 0. By asking this subtraction to be greater than m_i (instead of zero), we can accept more or less “fictitiously” drawn samples, such that their total number is equal to the number of original mutations of the gene in row “i”. This is a rather simple strategy to preserve gene alteration frequencies, but it turned out to be an extremely effective one as shown in **Figure R6**.

- Finally, I would like to see a more intuitive description of why subtracting uniform distribution from the expected mutation frequencies (obtained assuming independent and distinct Bernoulli random variables) followed by binarization is a reasonable approximation for obtaining binary matrices adhering to fixed marginals.

As already discussed in response to the previous comment, our sampling strategy was indeed not sufficiently explained. We have included in the methods (and briefly in the main text) a more detailed description, as also reported in the previous response. We hope this additional explanation and analyses clarify the approach that we took.

2. Experimental evaluation using simulations with known ground truth

- I think the paper would be greatly improved if there would be a simulation study with ground-truth pairs of mutually exclusive and/or co-occurring pairs of mutations. While I appreciate the comparison on real data (with down sampling experiments), I think a more direct comparison to ground truth would be more compelling. Ideally such a comparison would be used to compare against competing methods as well.

This is indeed a great suggestion and something that we hadn’t been able to address before. As part of this revision, we have now developed a new model to generate artificial data where we

can predefine evolutionary dependencies among pairs of genes, effectively generating “true” co-mutation patterns.

To create a set of “true” co-mutations that can be used to benchmark co-mutation discovery tools, we develop a model that generates mutations under the hypothesis of conditional selection of variants. The model requires to predefine: the total number of genes n and a range of gene mutation frequencies $\boldsymbol{f} = [f_{min}, f_{max}]$, the total number of samples m and a range of tumor mutation burdens $\boldsymbol{t} = [t_{min}, t_{max}]$, and a set of interdependent gene pairs $dG = \{ (g_1, g_2), \ldots, (g_r, g_s)\}$, such that if $(g_i, g_j) \in dG$, then mutating g_i (or g_j) will either promote (*synergistic interaction*, $sign(g_i, g_j) = 1$) or inhibit (*antagonistic interaction*, $sign(g_i, g_j) = -1$) mutations of g_j (resp. g_i). dG gene pairs are unformly sampled from the gene set with the constraint that each gene can be involved in at most one interpendent gene pair. (While this constraint does not reflect observations in real datasets, where a gene can be involved in multiple co-mutations, it simplified the generation of artificial datasets, by avoiding the risk of generating inconsistent interactions, and it did not affect any downstream analysis.) Next, n gene mutation frequencies and m tumor mutation burdens are sampled within the predefined ranges of values and assigned to each gene and sample.

To generate one sample, the model iteratively assigns one mutation to one of the genes with probabilities proportional to their mutation frequencies. At each iteration, once a mutation is assigned to a gene g_i, the probability of mutating that gene is set to 0 and if g_j exists such that $(g_i, g_j) \in dG$, then:

$$p\left(g_j = 1\right)^{k+1} = p\left(g_j = 1\right)^{k} + sign\left(g_i, g_j\right) * \varepsilon * p\left(g_j = 1\right)^{k}$$

Where $p\left(g_j = 1\right)^{k}$ is the probability of mutating the gene g_j at iteration k, and ε is a parameter indicating the amount by which a probability is increased $(sign\left(g_i, g_j\right) = 1)$ or decreased $(sign\left(g_i, g_j\right) = -1)$. Lastly, all non-zero probabilities are rescaled to sum to 1. This procedure is repeated until the number of mutations in the sample is equal to its predefined tumor mutation burden.

Results that are now introduced in this revised manuscript were based on an artificial dataset comprising 1000 tumor samples with a number of mutations varying between 5 and 30, across 100 genes, with mutation frequencies varying between 0.05 and 0.2. In total, 30 gene pairs were selected as interdependent, with 15 synergistic interactions, giving rise to 15 co-occurrently mutated gene pairs, and 15 antagonistic interactions, giving rise to 15 gene pairs mutated in a mutually exclusive manner. We set the parameter $\varepsilon = 0.95$, but highly similar results were obtained with $\varepsilon = 0.75, 0.85, 0.95$.

To demonstrate that this model effectively generates co-occurrent and mutually exclusive mutation pairs consistent with the predefined set of interdependencies, we generated a second dataset of the same size, using the same gene mutation frequencies and tumor mutation burdens,

but without specifying any interdependent gene pairs (i.e. $dG = \{\emptyset\}$). By comparing the co-mutation incidence of each gene pair in the two datasets, we could clearly see that co-mutations incidences differ substantially only among gene pairs that were set as interdependent in the first dataset (**Figure R8a**). Using this artificial dataset, we could compute the precision and recall of all benchmarked tools analysis in recovering the "true" evolutionary dependent gene pairs. Note that even in the artificial dataset, co-mutation patterns exhibited variable deviations from the expected incidence and especially for low-frequency genes, these deviations were small (**Figure R8a**). For this reason, we assigned a weight to each co-mutation in the precision and recall analysis, such that each tool is penalized less if it fails to recover a true interdependent gene pair whose co-mutation incidence was only moderately different from the expected one.

Across all comparisons, we found that only SELECT (our previously developed tool) and SelectSim obtained both good precision and recall, as shown by their F1 scores (**Figure R8b**). In contrast, all tools except DISCOVER exhibited good recall but low precision, i.e. their results were inflated by false positives. DISCOVER achieved good precision but low recall, indeed this tool correctly identified all the true mutually exclusive pairs, but none of the co-occurring pairs. This result once again demonstrates that the lack of co-occurring mutations that the authors originally reported in their study was actually due to the inherent limitations of this approach. We have now included these results in the manuscript in **Figure 1B** and presented in detail the approach in the revised Methods section.

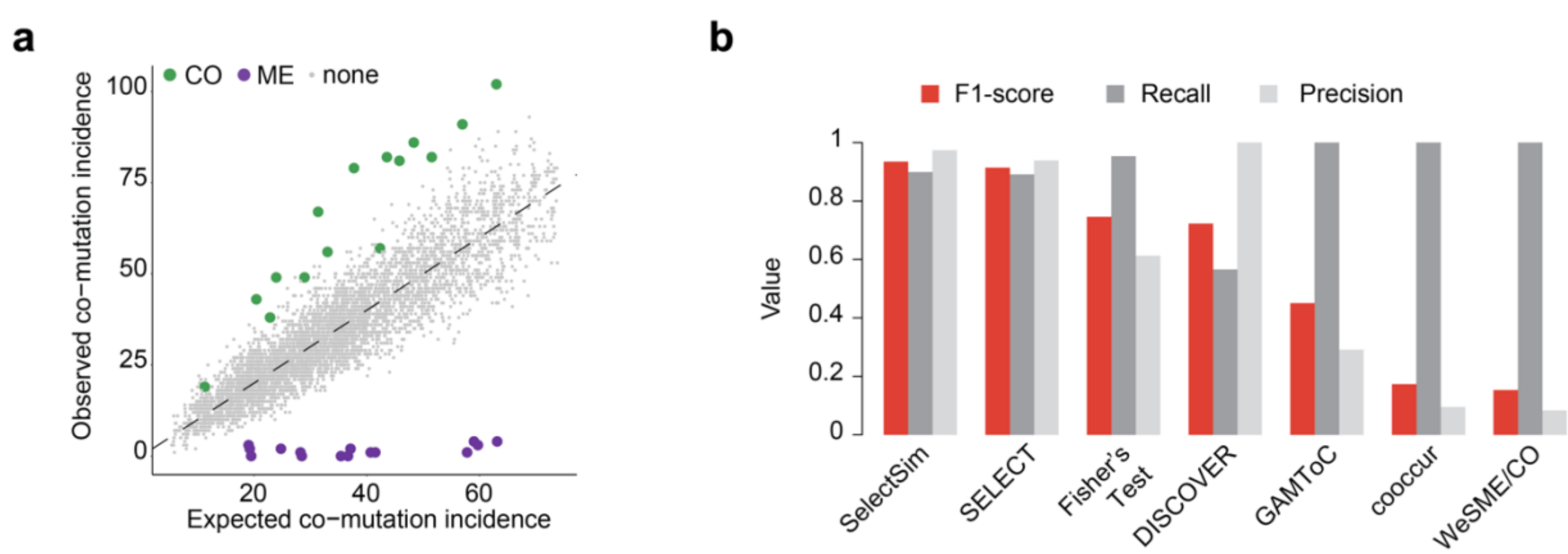

Figure R8: a) Observed vs. expected co-mutation incidence of true co-mutation based on artificial datasets generated with (y-axis) and without (x-axis) interdependent gene pairs. **b)** Precision (light gray), recall (dark gray), and F1 score (red) obtained by each method on the ground truth dataset comprising 30 pairs of evolutionary dependent genes, 15 pairs leading to co-occurring co-mutation patterns and 15 pairs leading to mutually exclusive co-mutation patterns.

- Please use simulations to assess the effect of the different parameters and threshold choices of SelectSim (such as \tau and \lambda, as well as the four categories listed in line 925).

As suggested by the reviewer, we used the newly generated artificial dataset with true co-mutation patterns to test precision and recall of SelectSim when varying lambda, tau, or the FDR intervals. Overall, both precision and recall were stable and maintained high values independently of

parameter changes (**Figure R10**) demonstrating the robustness of SelectSim. These results now also appear in **Suppl. Figure 1K,L**.

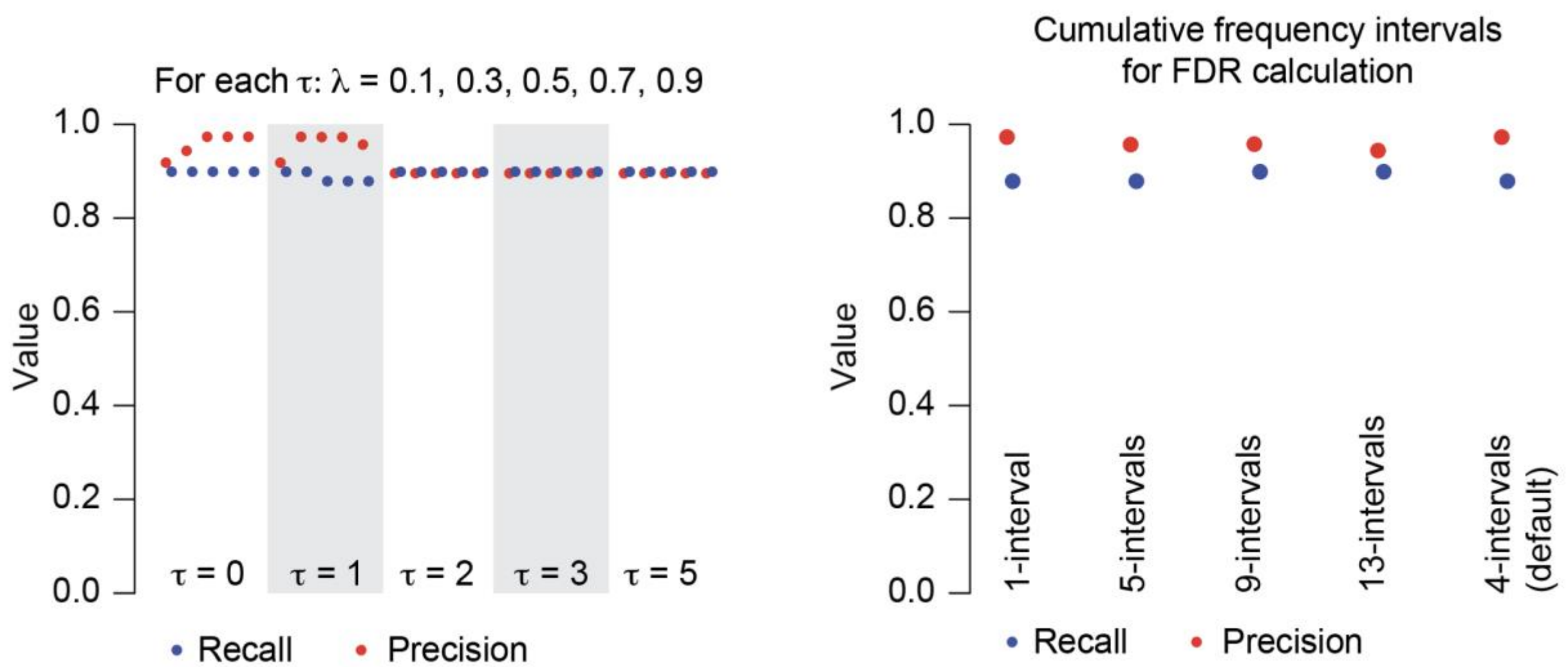

Figure R10: Precision (red) and recall (blue) values obtained by SelectSim using different lambda and tau parameters (left), as well as changing the frequency intervals for FDR calculation (right). All analyses were performed using our ground truth artificial dataset with 30 evolutionary dependent pairs and E = 0.95. Default intervals were used when varying lambda and tau, and default lambda = 0.3 and tau = 1 values were used when varying frequency intervals.

2. More detailed comparisons against standard approaches of generating binary matrices with fixed marginals.

- The paper mentions a permutation-based approach for generating binary matrices with fixed row and column marginals (BiRewire). The only results I could find is a modest reduction (by at most a factor of 2) in running time. What about the results? Are they in agreement? Ideally, this would be assessed with simulations with known ground truth.

We may have failed to mention this explicitly, but in fact BeRewire is internally used by SELECT (our previously developed tool) which is included in the benchmarking analysis. Results from SELECT and SelectSim are largely consistent in terms of precision and recall, but SelectSim is more robust to downsampling and on TCGA data it only identified co-mutations associated with putative oncogenic variants, whereas SELECT recovered significant co-mutations also among synonymous variants, which are likely to be false positive. We should also note that while the reported reduction in running time is modest, that reduction was obtained by comparing a highly optimized implementation of the BeRewire model, with a simple R script implementing our simulation strategy. Although beyond the immediate goals of this study, we expect that proper code optimization (expected with the next stable release) will significantly further reduce the running time of our approach

3. More detailed discussion of the limitations of the approach.

We thank the reviewer for these additional suggestions which we now more extensively discussed in the Discussion section of the revised manuscript. For some of these points, we were actually able to provide additional analyses, which we believe strengthen our work. Details are presented below.

- Beyond pairs

This is indeed still a limitation of the approach and something that will need to be addressed in future studies. We now discuss this limitation in the manuscript.

- Intra-tumor heterogeneity, subclonal mutations may confound analysis

While this is true, we believe that the impact of subclonal mutation will be small. We tried to investigate this effect using inferred tumor subclonal compositions from the TCGA cohort (see Raynaud et al 2018) and we found no strong evidence to suggest that sub-clonality of specific variants alter our results. *For that to happen we should recurrently detect co-occurring mutation pairs that never co-occur in the same cell*. While potentially fascinating, we cannot find such recurrent patterns in the data. We should stress however that subclonal variant estimation in TCGA samples is still an error-prone approximation and, thus, we cannot draw a solid conclusion.

- How to more naturally account for difference between mutation types

We are not sure what the reviewer is here referring to when they say: “more naturally”. SelectSim provides a rather straightforward way to account for different mutation types by simulating independent gene alteration matrices for each mutation type, each accounting for mutation type-specific gene alteration frequencies and tumor mutation burdens. In our study, we used this approach to discriminate among missense and truncating mutations, but the same method can be easily adapted to account for user-defined mutation type classifications (e.g. by separately simulating mutations corresponding to different nucleotide substitutions). This is mentioned in the text.

- Power analysis?

Using our ground truth artificial dataset, we were now able to perform a power analysis to assess the number of samples required to discover true co-mutation patterns as a function of the cumulative gene alteration frequency of co-mutated gene pairs and the average tumor mutation burden in the dataset.

To do this, we generated 3 artificial datasets, each comprising 10000 samples and 100 genes with mean TMB equal to 15, 20, and 30. Next, we ran SelectSim on these dataset and on multiple

subsampled datasets with sizes ranging between 500 and 10000 samples. The fraction of recovered true co-mutations was separately calculated within 3 groups defined by the cumulative gene alteration frequencies of the co-mutated pairs.

As shown in **Figure R11** (now also **Suppl. Figure 5**) with as low as 750 samples all co-mutation patterns were recovered among gene pairs that together were mutated in at least 10% of the samples. For lower cumulative frequencies more samples were required and for the lowest average TMB, not all co-mutation patterns could be recovered even in the whole dataset.

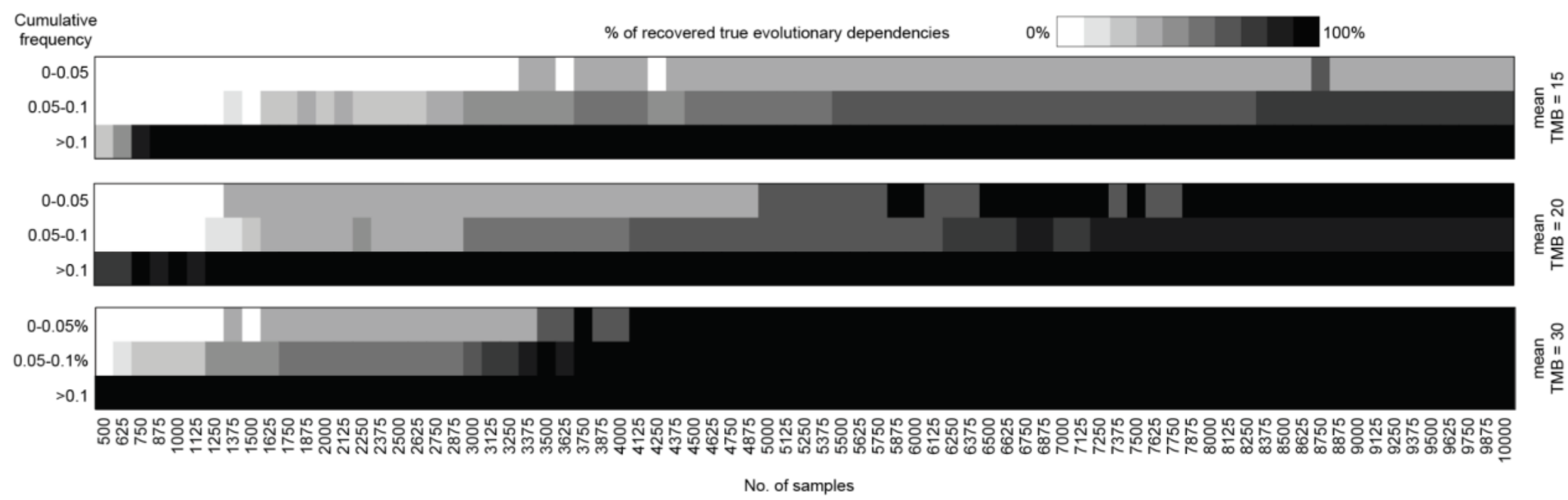

Figure R11: Fraction of true co-mutations that were recovered as a function of the number of samples in the dataset (x-axis) and mean tumor mutation burden (TMB) in the dataset (one heatmap for each mean TMB). Each row indicates the cumulative frequency range of the evolutionary dependent gene pairs.

- Mutation level instead of gene level

This is an interesting point that the reviewer is asking. Indeed, it is known that different mutated residues of the same gene may lead to distinct functional consequences and, as such, they may induce different evolutionary dependencies and co-mutation patterns. However, to separately analyze co-mutation patterns among specific mutated amino-acids, rather than genes, would drastically reduce alteration frequencies in the dataset and, with that, the statistical power to discover co-mutation patterns. Nevertheless, we tested this possibility for recurrent hotspot mutations within a selected set of frequently altered oncogenes: *BRAF*, *KRAS*, *EGFR*, and *PIK3CA*. For each gene, we selected either specific hotspots or group of hotspots defined based on prior knowledge, sequence proximity, or protein domains. Even in these conditions, some hotspots were significantly more frequent than others, and we observed that co-mutations detected at gene level were often coincident with those observed for the most frequent hotspot (**Figure R12**). Overall, co-mutations (when detected) were largely consistent across hotspots, although for less frequent hotspots only few significant co-mutations were found. Notably, among the few exceptions, *BRAF* class 2 and 3 mutations exhibited co-occurrences with *NF1* and *NRAS,* which are typically mutually exclusive with BRAF class1 mutations, and BRAF mutations analyzed at gene level (**Figure R12**). This result is likely associated with the different dependencies on *KRAS* signaling of *BRAF* mutation classes (see Yao et al. Nature 2017). While intriguing, these

results should be taken with a grain of salt, given the variable and overall low mutation frequencies of the hotspot classes. For this reason and given the overall consistency of the results that we observed (**Figure R12**), we would suggest to not include this analysis in the manuscript, unless the reviewer feels strongly otherwise. We now mention this limitation in the discussion.

Gene	Hotspot Category	Residue(s) or Range	Annotation / Domain	Class
BRAF	BRAF_1	V600	–	Class-1
	BRAF_2	R462, I463, G464, G469, E586, F595, L597, A598, T598, K601, A727	–	Class-2
	BRAF_3	K484, N581, D594, G596, G466, S467, G469	–	Class-3
EGFR	EGFR_1	185–338	Furin-like domain	Class-1
	EGFR_2	713–790	Tyrosine kinase domain	Class-2
	EGFR_3	800–900	Tyrosine kinase domain	Class-3
	EGFR_G598	G598	–	–
	EGFR_R108	R108	–	–
KRAS	KRAS_G12	G12	–	–
	KRAS_G13	G13		
	KRAS_Q61	Q61		
	KRAS_A59	A59		
	KRAS_A146	A146		
	KRAS_K117	K117		
PIK3CA	PIK3CA_1	32–118	PI3K_p85B domain	Class-1
	PIK3CA_2	341–483	PI3K_C2 domain	Class-2
	PIK3CA_3	520–703	PI3Ka domain	Class-3
	PIK3CA_4	798–1014	PI3_PI4_kinase domain	Class-4
	PIK3CA_5	1015–1068	End of PI3_PI4_kinase domain	Class-5

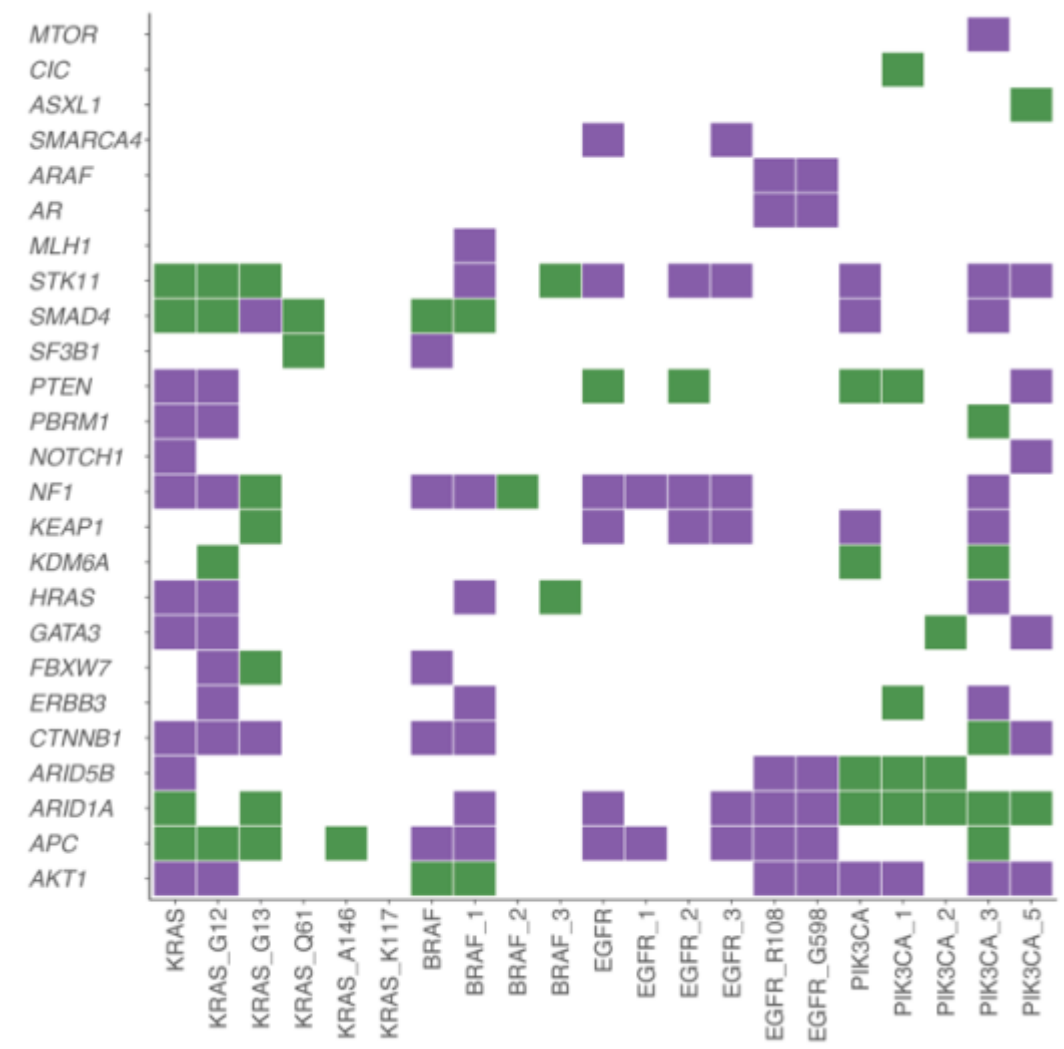

Figure R12: Co-mutations among specific variant classes: (Left) Classification of hotspot or hotspot classes for 4 oncogene. (Right) Co-mutations identified by SelectSim at the gene level and at the level of hotspots or hotspot classes.

Minor:

- what is the difference between "statistically signifcant reccurent mutations" (line 60) and "more or less frequent than expected by chance" (line 63)?

In that paragraph, we are drawing a parallel between challenges faced when searching for individual cancer driver genes, and when searching for significantly co-mutated genes, which is the focus of this study. The lines cited by the reviewer refer, first, to "statistically significant recurrent mutations", intended as individual variant or individual genes, and, second, to genes that are concurrently mutated "more or less frequent than expected by chance", i.e. co-mutated gene pairs. We clarified the text.

- line 76: include citations after "few approaches"

The citation appears at the end of the sentence and refers to a review that compared in more detail features and limitations of several approaches designed to study mutually exclusive or co-occurrent mutations.

- Include a feature table to discuss features and limitations of SelectSim as well as other methods. There are approaches that consider fixed row and column sums. One such approach is:

[1] Mark DM Leiserson, Matthew A Reyna, Benjamin J Raphael, A weighted exact test for mutually exclusive mutations in cancer, Bioinformatics, Volume 32, Issue 17, September 2016, Pages i736–i745, https://doi.org/10.1093/bioinformatics/btw462

While a comprehensive table comparing features of more than 20 different tools is provided in our recent review (Mina et al. 2022), which is also referenced in the text, we provided here an adapted extract of that table focusing specifically on the methods that we benchmarked in this study. This table now appears as **Suppl. Table 1**.

Reviewers' Comments (V2):

Reviewer #1 (Remarks to the Author):

I appreciate the authors detailed answer to earlier comments. I certainly appreciate the authors expertise in co-mutational analysis. New normal tissue co-mutational analyses is novel.

We are glad to hear that the reviewer appreciated our revised analyses and in particular the novelty brought in by the co-mutation analysis in normal tissues. As detailed in our response below, we have now addressed all the remaining concerns and hope the reviewer will consider this further revised manuscript ready for publication.

What still remains unclear from the manuscript and responses to comments:

1) What is the conceptual advances in SelectSim that were not available in the 2020 Nat Gen SELECT paper? Some performance improvement? It would be great to see this in very specific terms of the model changes and math.

Although both SELECT and SelectSim were developed in our group, these are completely different methods, as extensively discussed in the Methods section of this manuscript, as well as in the main results. Moreover, SelectSim demonstrated significantly better performances both in terms of robustness and quality of the results, as we have now more comprehensively evaluated and showed with new analyses.

In terms of model changes, SELECT generates empirical null models via data permutation, preserving the marginal distributions of the data matrix (i.e., gene alteration frequency and tumor mutation burden). Instead, **SelectSim** estimates the likelihood of a given gene to be mutated in a given sample, based on gene mutation frequencies and tumor mutation burdens, to then simulate cancer mutation datasets. In addition, co-mutation incidence in SELECT (and in all other approaches) is simply computed by counting the number of samples exhibiting mutations at both the queried genes. Conversely, **SelectSim** introduces a strategy that weights samples as a function of their mutation rates. The rationale is that co-occurring mutations are "more surprising" in samples with low mutation rate than in samples with high mutation rates. We show in the manuscript that this strategy is required to capture significant co-occurrences that would be missed otherwise. Finally, not only **SelectSim** allows to account for different tumor types, subtypes and genetic alterations, but it is the only tool that allows discriminating between different mutation types/signatures. Indeed, in this study, **SelectSim** was used to independently simulate (and then integrate) missense and truncating mutations, the rates of which differ among different tumor types and subtypes (e.g., MSI tumors have a

higher rate of truncating mutations, a property that cannot be accounted for by any of the available tools). All these methodological innovations introduced with **SelectSim** are mentioned in the main text and extensively described in the Methods in the “SelectSim Algorithm” section.

In this revision, we have more extensively compared the results returned by SELECT and **SelectSim** on the TCGA dataset, to better interpret the strengths and weaknesses of each approach. We found that the major differences boil down to the strategies employed to account for gene mutation frequencies and tumor mutation burdens.

First, we tested the mutation frequency of each significant gene pair (here defined by the total number of samples harboring a mutation in at least one of the genes in the pair over the total number of samples), based on whether the pair was found significant by:

- **A)** both SELECT and SelectSim,
- **B)** only SelectSim,
- **C)** only SELECT.

Interestingly, while pairs in the **A** and **B** categories exhibited similar frequency distributions, pairs in category **C** exhibited significantly lower frequency, i.e., pairs found significant only by SELECT typically involved genes mutated at a low frequency. This may be due to a specific frequency correction implemented in SELECT when computing the mutual information score from the binary alteration matrix (see Mina et al. Cancer Cell 2017). We reasoned that this correction may however inflate the significance of small effect sizes driven by samples with a high mutation burden. We tested this hypothesis by comparing the tumor mutation burden of co-mutated samples in gene pairs in categories **A**, **B,** and **C**. Strikingly, we found again that while gene pairs in categories **A** and **B** exhibited similar differences in mutation rates between co-mutated and wild-type samples, gene pairs that were significant only according to SELECT (category **C**) exhibited a much bigger difference, showing that gene pairs found significant only by SELECT were *biased for co-occurring mutations in samples with very high mutation burdens*. This result suggests that <u>**SelectSim** significantly better accounts for the differences in tumor mutation rates across different tumors, and that failing to do so leads to overestimating co-mutation significance</u>. These results have now been included in the manuscript and shown in **Supplementary Fig. 1K,L**.

2) AF or CCF are not employed as a covariate, though it would seem as very important. Purity of the sample (not used) can also lead to falsely missed co-cooccurrence patterns. Those are main drivers of heterogeneity.

The reviewer is correct and raises an interesting point. To address this concern, we retrieved tumor purity estimates and variant allele frequencies (VAF) when available in

our cohorts and tested whether these were associated with mutation and co-mutation incidences, and, thus, with SelectSim scores.

First of all, tumor purity may affect our results by "hiding" true cancer mutations, i.e., mutations may be missed in samples with low purity, especially if sub-clonal. While this may certainly be true in specific cases, to affect our results this should be true systematically and result in a *positive correlation between the number of mutations detected in a sample and its purity*. We tested this hypothesis in both TCGA and MSK cohorts, but in both cases no correlation was found (Spearman's cor_{TCGA} = -0.042, cor_{MSK} = 0.0071, see **Fig. R1A**).
Next, we specifically tested potential biases introduced by variable VAF for the mutations that we analyzed. Indeed, VAF can vary because of both tumor purity and tumor clonality (i.e. variable cancer cell fraction). For each significantly co-occurrent (CO) or mutually exclusive (ME) gene pair, we examined VAF values in samples exhibiting a mutation in only one of the two genes (single-mutant) or in both genes (double-mutant). VAF differences between these two groups would invalidate our results in the following cases:

- Significant lower VAF in double-mutant samples vs. single-mutant samples may suggest that although double-mutant samples harbor mutations at both genes, these may be sub-clonal and possibly not occurring in the same cells. If so, such evidence may invalidate significant co-occurrence patterns. (*Significant mutual exclusivity would not be invalidated, as such a scenario would make the mutual exclusivity even stronger.*)
- Significant higher VAF in double-mutant samples vs. single-mutant samples may suggest that mutation incidence in single-mutant samples is underestimated and therefore additional variants may be present that were missed. If so, such evidence may invalidate significant mutual exclusivity patterns. (*Significant co-occurrence would not be invalidated, as such a scenario would make the co-occurrence even stronger.*)

To perform this analysis, we analyzed all CO and ME gene pairs comprising at least 10 double-mutant and 10 single-mutant samples and that were found significant in the pan-cancer TCGA dataset. Expected VAF differences (i.e., the null distribution) were estimated by permuting single- and double-mutant sample labels. Upon comparing VAF values between single-mutant and double-mutant samples, we found no evidence of systematic bias (**Fig. R1B**): only 3 hits out of 232 barely exceeded the 95% confidence interval of the null distribution and entered into the "problematic conditions" that we described above (**Fig. R1B**). Nevertheless, we looked at these 3 cases in more detail: in all 3 cases, the difference in VAF was modest and actual mean VAF values were always above 0.25, unlikely to correspond to true sub-clonal variants. (In our opinion, the only

dubious case may be the co-occurrence of *CDKN2A* and *KRAS* mutations, which in double mutant samples have a mean VAF close to 0.25, yet these mutations are among the most recurrent in pancreatic and lung cancer and have been already reported as co-occurrent – see PMID: 38666907, PMID: 26069186. We don't think there is enough evidence here to claim that these variants co-occur in the same tumor but not in the same cells.) Overall, we think that these analyses convincingly show that while we cannot exclude that in some samples a few variants may have been missed or co-occur in different clones, *these effects are not systematically observed and do not invalidate our results*.

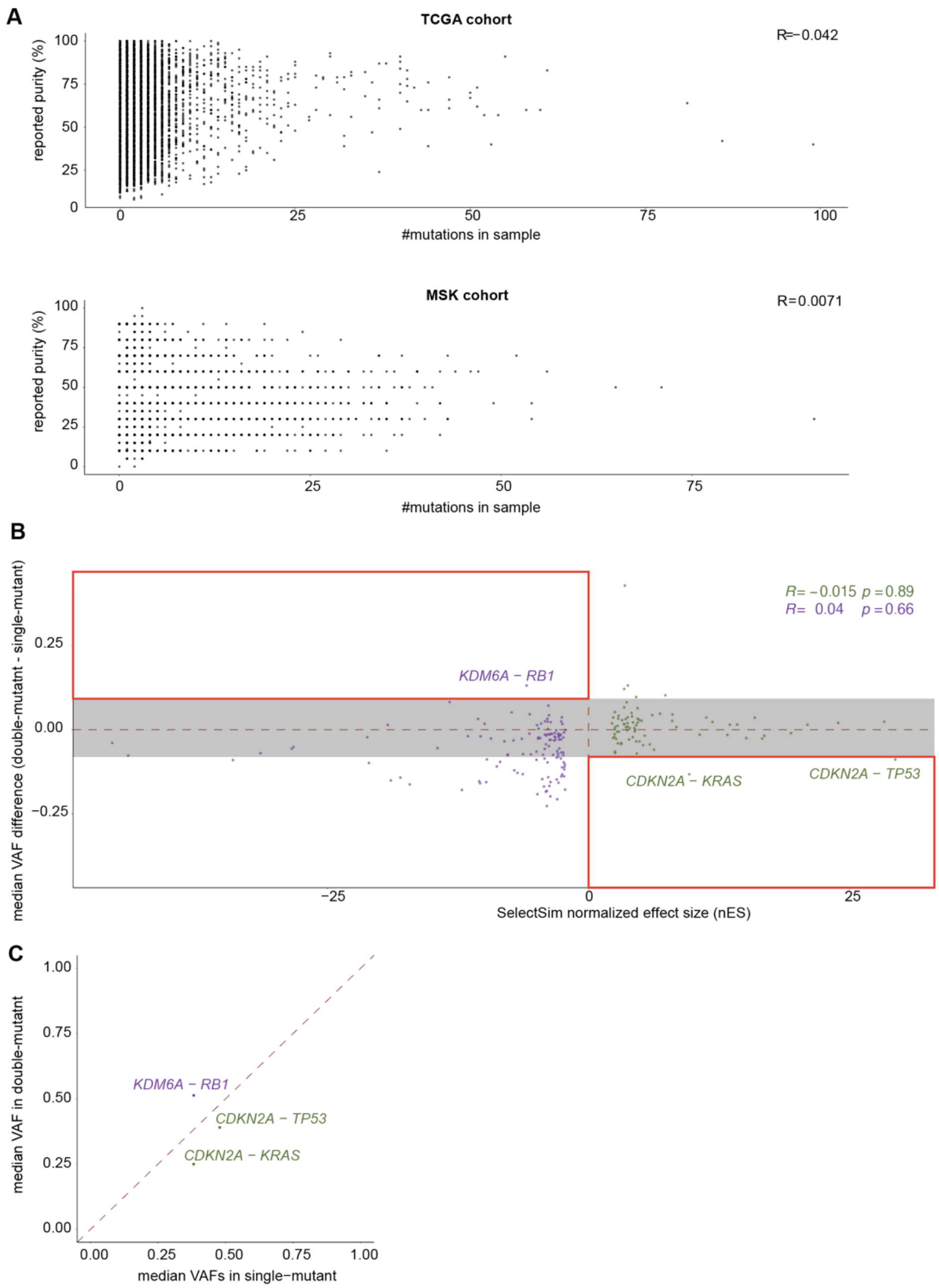

Figure R1: A) Number of mutations per sample (X-axis) vs estimated tumor purity per sample (Y-axis) in the TCGA (top) and MSK (bottom) cohorts. **B)** SelectSim normalized effect size (nES, X-axis) vs. difference between the median variant allele frequency (VAF) between double- and single-mutant samples (Y-axis) for significant co-mutations in the TCGA cohort. **C)** Median VAF for selected co-mutation in single- (X-axis) vs double-mutant (Y-axis) TCGA samples.

3) Authors mention that mutational processes/signatures co-variates are implied, but are not really discussed in the manuscript. No known mutational processed are used even if available for some cohorts.

We apologize for the lack of clarity on our side concerning this point. As discussed in detail in the Methods section, SelectSim is designed in a way that allows to account for mutations generated through distinct mutational processes, by independently estimating the likelihood of distinct mutation types for each gene (see "Step 1-bis" in the "SelectSim Algorithm" section). In this manuscript, we demonstrated this possibility by independently estimating the likelihood of missense and truncating mutations. To do so, we separately estimated the frequencies of these variant types in each gene *g,* $f_{miss}(g)$ and $f_{trunc}(g)$), and the tumor mutation burden of these variant types in each sample *s*, $TMB_{miss}(s)$, and $TMB_{trunc}(s)$. *These mutational processes have been accounted for in all analyses presented in this manuscript*. Even if theoretically possible, we decided to don't use more detailed mutational signatures, based on either specific single nucleotide changes or even accounting for trinucleotide contexts, as it required assigning each observed mutation to a specific signature, which is an error-prone exercise and would result in very sparse datasets. We agree with the reviewer though that with increasingly larger datasets, this could be a promising avenue to pursue in the next future and something that now is possible to do using SelectSim. We mention this point in the Discussion section.

4) Given extensive prior work in the field and by the current group on the topic it is very important to highlight in the text and discussion what has been known as interaction between mutations, what is found due to the novel model presented, what is found just by the sheer increase in available data to 70k tumors. Very importantly authors mention incorrect results from other studies that are rectified with the new approaches - could those be enumerated and explained - this seems very important to correct the field if this is the case.

This is indeed an important matter that we tried to address in different ways. First, throughout the manuscript we repeatedly referenced previously published functional or clinical evidence supporting our reported co-mutations, and highlighted previous unreported co-mutations of particular interest. This is done in particular in reference to Figure 2, where we report significant co-mutations associated with most common tumor types, and in Figure 3, where we discuss co-mutations associated with metastatic

progression. This is however a rather qualitative assessment of our contribution to the field.

Next, in a more quantitative manner, our work "rectifies" previous results in two main aspects:

1) SelectSim better accounts for variable mutation rates across tumors than previous tools. We already demonstrated SelectSim superior specificity in recovering synthetic co-mutations with respect to most tools that are inflated for false positives (**Figure 1B**) and our new detailed comparison with SELECT well highlights the risk of overestimating co-occurrence in samples with very high mutation rates (see new **Supplementary Fig. 1K,L**). A key distinction between SELECT and SelectSim is that the former estimates the tumor mutation burden exclusively from the subset of genes used in input by the tools and only from the mutations retained in the analysis, while the latter accounts for all mutations and all sequenced genes, providing better estimates of the TMB variability.

2) By quantifying co-mutation rates accounting for individual tumor mutation burdens, SelectSim was able to recover significant and functionally relevant co-occurring mutations, "rectifying" the previously reported absence of significant co-occurrence. We discuss this aspect at length in our detailed comparison with DISCOVER, which shares algorithmic aspects with SelectSim but is unable to detect co-occurrences both in synthetic and real datasets.

Finally, as the reviewer suggests, the "sheer increase in available data" provides unprecedented statistical power for our approach. However, to benefit from this dataset, it is critical to scale efficiently with large mutational datasets, something that we significantly improved with SelectSim with respect to our previously developed SELECT, and especially being able to account for great sample diversity, characterized by multiple tumor types, subtypes, and mutation rates. As shown in Figure 1D (and in other benchmarking analyses in Suppl. Figure 1), the inability to account for these confounders invariably leads to high numbers of false positive results.

We made these points more explicit in the text to make sure the innovation brought by SelectSim is clearly described.

5) Would be great if survival analysis/ coxph is accomplished in regards to co-occurrence on at least the cohorts that have clinical annotations.

We agree with the reviewer that this is an interesting analysis to add. Starting from the independently validated set of co-mutations presented in **Figure 2**, we selected the most

frequent combinations in each tumor type and tested whether they provide a prognostically relevant patient stratification, using the extensively curated clinical data available for MSK-derived cohorts. For multiple tumor types, we did find that co-mutations allowed to stratify patients into groups with significantly different overall survival. All these results are now discussed in the text and reported in the new **Supplementary Figure 4**.

6) Are metastasis specific pairs due to progression? or they really are mutually exclusive first and then become co-mutated?

I think here the reviewer is raising two interesting points at once.

First, if the reviewer is here asking whether we have actual evidence of changing co-mutation patterns between primary and metastatic samples derived from the same set of patients (i.e., matching samples), we need to clarify that primary and metastasis cohorts from both MSK and DFCI largely comprise un-matched samples. *However*, the scale of these datasets guarantees proper representation of primary and metastatic conditions and, thus, can be considered representative of the actual disease progression.

Second, when the reviewer says: “they really are mutually exclusive first and then become co-mutated”, they seem to allude to the possibility of acquired secondary mutations that increase metastatic capacity. Lacking matching samples, we cannot really test this hypothesis versus the alternative scenario where tumors exhibit or not a specific co-mutation from the beginning, and this changes the probability of developing metastases later on (the problem is analogous to the one of discriminating between pre-existent or acquired variants associated with treatment resistance). Note, however, that even if this problem cannot be addressed without matching samples, *it doesn’t diminish the relevance of these co-mutations as potential biomarkers of metastatic progression*.

7) The field has always been interested in more than pairs - i.e. triplets, quads. Does this method and new large dataset answer those questions?

The reviewer is correct, and this is a limitation of the current version of SelectSim and something that we hope to address in future studies. As suggested below by the reviewer, we explicitly mention this limitation in the Discussion section.

8) Would be great to see detailed Q-Q plots for all scenarios.

We have added detailed Q-Q plots for all datasets in the new **Supplementary Figure 2B**.

9) discussion should be expanded to cover all the above topics and really highlight important novel findings

As mentioned in response to the previous questions, we substantially edited the Discussion section to include the issues raised by the reviewer.

Reviewer #1 (Remarks on code availability):

The code repo seemed to have not been updated during revision?

The code repository has been updated and well-documented, as also highlighted by the other reviewers.

Reviewer #2 (Remarks to the Author):

General assessment

The manuscript has been substantially revised, with clear improvements compared with the previous version. The performance analysis of the co-mutation method is more robust. The study with somatic mutations in normal tissues, which has been completely reformulated, is more compelling.

The study does not only provide a tool that is bound to be of practical utility in somatic evolution genomic studies, but it also portrays a relevant case in the form of metastasis risk assessment based on differential co-mutation enrichments, and it also provides a novel and in this reviewer's opinion important insight into the question of whether and to which extent the somatic co-mutation patterns in normal tissues recapitulate or resemble the genomic features known in tumor samples.

The results of the normal tissue study make sense in the light of a multistage model of carcinogenesis whereby a relatively limited repertoire of sequential genomic events would allow the carcinogenic transformation, suggesting that in normal tissues a significantly more diverse repertoire of mutant combinations are possible, hinting towards a model whereby the clonal landscape is dominated by competition and convergent evolution. Unlike with the first review, I am more convinced that the results of this analysis are at the same time robust enough and interesting in the context of somatic genomic studies.

The authors have addressed to a substantial extent the concerns and comments raised by the reviewers. This reviewer is overall satisfied with the answers provided to their comments in the first review. I commend the authors for their work in improving the study and thoroughly replying to the reviews.

We thank the reviewer for the supportive and constructive feedback that helped us improve our work and for appreciating the work we did during the first revision of the manuscript. Below we addressed their additional notes, which further demonstrated the robustness of our results.

I noted down a couple of additional comments which would not necessarily require a direct answer – as far as this reviewer is concerned – but would nevertheless appreciate if the authors and editors consider them for the sake of improvement, should the manuscript be published.

Specific additional comments

- Regarding the regression analysis of metastatic risk, I wonder whether encoding the treatment into 40+ treatment types may be slicing the treatment factor too thinly for any single feature or set of features to show a consistent effect. One alternative would be grouping into broader treatment categories, which could also be tricky, given the heterogeneity of treatments and therapeutic effects, but also considering the critical importance of treatment combinations and regiment. The authors should consider, before drawing a negative conclusion regarding the treatment effects, whether the analysis can be conducted with broader treatment features classes to catch any potential interactions with treatment.

- Did the authors consider or conduct at some point this metastatic risk regression analysis by adjusting for age and/or some relevant factor or exposures such as smoking, whenever this metadata is available? I wonder whether there might be an age component, which the current analysis does not clarify, as to whether some co-mutations predict progression into metastasis. For example, do pro-metastatic co-mutations arise more frequently in older vs younger patients, or do they carry a stronger predictive effect in older vs younger patients, or vice versa? What about other exposures for which there is metadata in GENIE? I think it would be important to clarify, and I believe there is an opportunity to do so at little extra cost, if the clinical metadata permits, whether this risk effect is independent of age.

Both the comments above hint at different covariates that may affect our ElasticNet analysis.

First, we agree with the reviewer that the size of the dataset and the number of possible treatments may challenge our ability to capture significant associations with treatment. To address this point, we repeated the ElasticNet analysis grouping the treatment variables either based on their common joint administration (drug combinations), their targets (PD1, ER, AR, EGFR, PGR, VEGF, HER2, MTOR, CDK4/6, general chemotherapy), or based on their broad categorization (immunotherapy, hormonal therapy, targeted therapy, chemotherapy). The stratification by target highlighted potentially interesting associations, such as lower metastatic risk for anti-PD1-based treatment and increased metastatic risk for anti-VEGF (**Fig. R2A**), whereas most of the other variables exhibited modest effect sizes (ElasticNet coefficient). Second, in these analyses, we included age and smoking status as covariates, but neither of those exhibited strong predictive power (**Fig. R2B**). Lastly, we examined the co-mutations that

were found as predictive in these different ElasticNet runs. Strikingly, the results were highly consistent, with 34 out of 34 (100%) co-mutations found predictive of metastatic progression, and 36 out of 48 co-mutations found predictive of the number of metastases in all 4 types of analysis (**Fig. R2C** and new **Supplementary Fig. 5D**). Overall, while annotations for this dataset might be limited to properly classify as predictive specific treatments, we robustly associated co-mutations with metastatic risk independently of the type of treatment received by the patient.

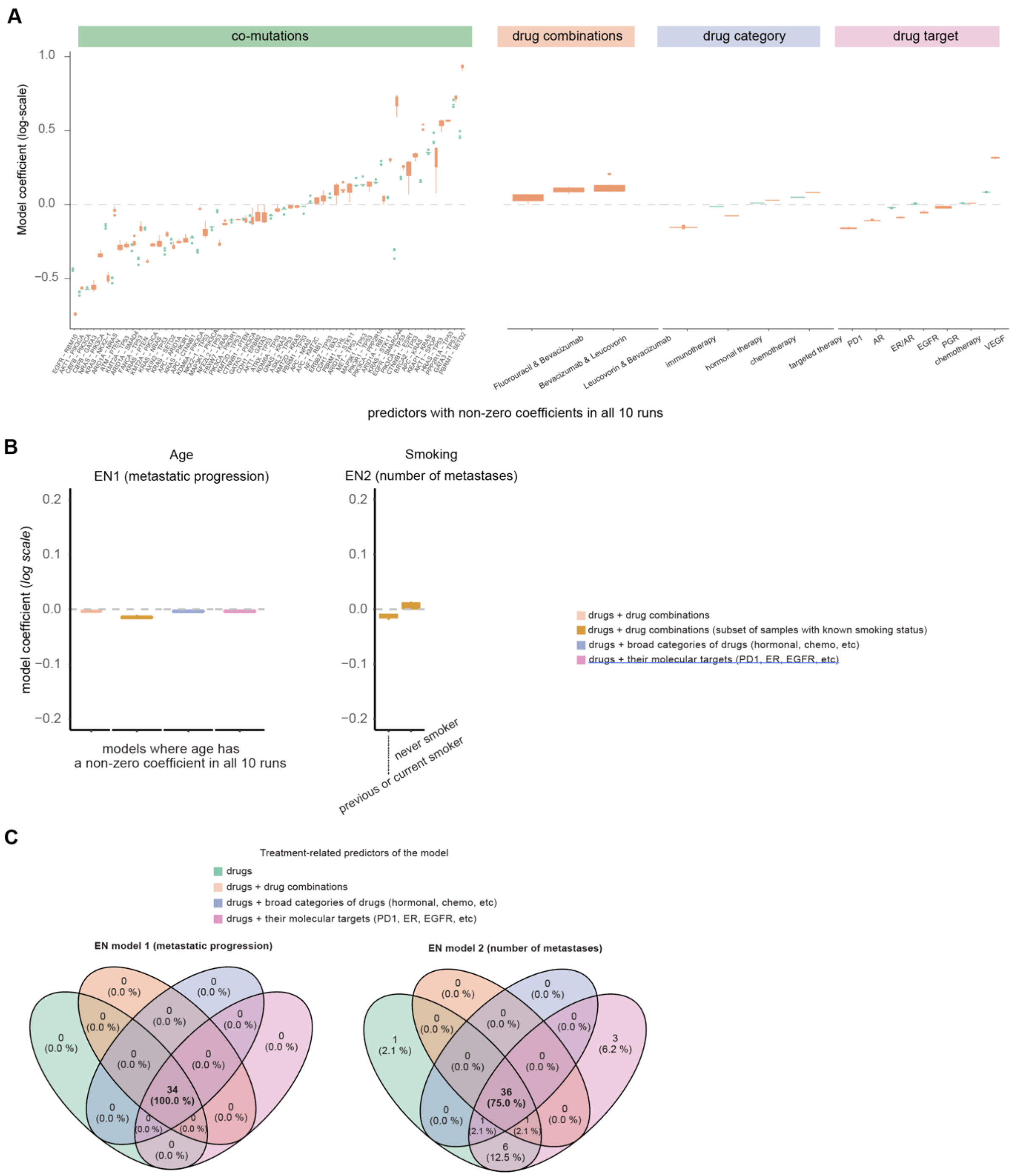

Figure R2: A) ElasticNet coefficients of grouped variables: co-mutations, drug combinations, broad classes of drugs, and drugs grouped based on their molecular target. **B)** ElasticNet coefficients of age and smoking status in different models. Only variables with non-zero coefficients across all 10 repetitions of model training are plotted in **A)** and **B)** panels. **C)** Venn diagrams of co-mutations with strong predictive power across models that include different groups of treatments as variables.

- Although the writing has improved – particularly when it comes to the method descriptions, where the previous version had some issues – there are still some parts that could be written more clearly. As an example to illustrate the type of concern, this reviewer found the explanation in p.23 where the fundamentals of the main statistical approach are laid out, still a bit troublesome. Despite the unavoidable subjective component of this assessment, I can point out to three problems that permeate this part of the manuscript: i) Non-standard concepts. The notions of proposal and desired distribution, the concept of a fictitiously drawn sample, are not standard and were not particularly helpful for this reviewer. ii) Notational inconsistencies. S is given two non-equivalent definitions, confusing the audience: wouldn't it be preferable to just denote them differently? iii) Non-specific descriptions. The alluded construction with the plus/minus epsilon that pursues to attain some prescribed marginals by tuning the rejection threshold can (and should) be described in a completely univocal way.

We rewrote this section to improve its clarity and made sure to use a consistent notation throughout the manuscript.

Minor issues

- Two equations in page 22 have some typo / formatting errors
- Instead of "true", it feels more appropriate to use something like "ground truth" or "synthetic" to refer to data that you are simulating.
- Please, be mindful to always use an appropriate numerical scale for the axes of the plots, e.g. Supp Fig 6B

All these issues have now been addressed.

Reviewer #3 (Remarks to the Author):

I thank the authors for the detailed point-by-point response. My previous comments have been addressed. I have no further comments.

Reviewer #3 (Remarks on code availability):

I have reviewed the GitHub page. The README is clear, with clear installation instructions, and links to further documentation including an example. Simulations are available on zenodo.

We thank the reviewer for the positive feedback and their previous suggestions that helped us improve this manuscript.

Reviewer #1 (Remarks to the Author):

I appreciate the authors's revisions and they addressed most of the previously raised points. One last remaining point is:

8) Would be great to see detailed Q-Q plots for all scenarios.

We have added detailed Q-Q plots for all datasets in the new Supplementary Figure 2B.

The authors seem to have provided (Sup Fig 2B) a Q-Q plot vs Normal Distribution (or similar), this it's not what was asked - Please provide a proper plot(s) of observed p-values vs. expected uniform quantiles -log(p). I am a bit surprised if the authors have not done it themselves previously as one of the best ways to check if the test is well-calibrated against the null model and that the p-values are not inflated/deflated. This is very important for us to be able to assess the test calibration properly. https://stats.stackexchange.com/questions/92124/how-to-interpret-a-qq-plot-of-p-values

We apologize with the reviewer for the lack of clarity in our previous response. The reviewer is correct in their definition of QQ-plot for p-values, but what the reviewer may have missed is that the procedure they suggested cannot be applied in our case since SelectSim does not use p-values.

Indeed, SelectSim adopts an approach that allows to estimate the false discovery rate (FDR) of a test directly from the test statistic, without explicitly calculating p values. This procedure was introduced in a seminal study by **Tusher, Tibshirani, and Chu** in 2001 (https://www.pnas.org/doi/10.1073/pnas.091062498) and it relies on estimating the FDR associated with a given threshold T, as the ratio between the number of observed effect sizes exceeding T and the number of effect sizes drawn from a null distribution that exceed T (in their original study, an empirical null distribution was derived by permuting the samples' labels). As described in the Methods section of our manuscript, SelectSim estimates the expected number of false positives from its simulated alteration matrices and select T that guarantees an FDR below a given α. (Note that the procedure indicated at the link suggested by the reviewer **can only be applied to p-values** – which under the null hypothesis has a uniform distribution, but cannot be used for FDR values.)

For this reason, in our previous response we decided to show QQ-plots of the effect sizes (instead of p-values), generated by comparing their observed and expected quantiles under the assumption that expected effect sizes followed a normal distribution. While this is a common approach, we agree with the reviewer that this procedure may be sub-optimal as the null model defined through our simulations does not necessarily follow a normal distribution. To better compare the observed and expected quantiles of the effect sizes in SelectSim, we generated new scatterplots by directly comparing the distribution of observed effect-sizes with the actual distribution of expected effect sizes generated in our simulations. Note that the same procedure was proposed in the Tusher study (*see Figure 3A of their manuscript*).

These new scatterplots (copied below), once again confirm that while the bulk of the observed and expected distributions are well aligned (black data points), significant effect sizes at the tails of the observed distribution deviate considerably from the expected quantiles (red data points). These plots provide evidence that the test is well calibrated, and that significant observed values are likely true positives. These plots have now been included as new **Suppl. Figure 2B**.

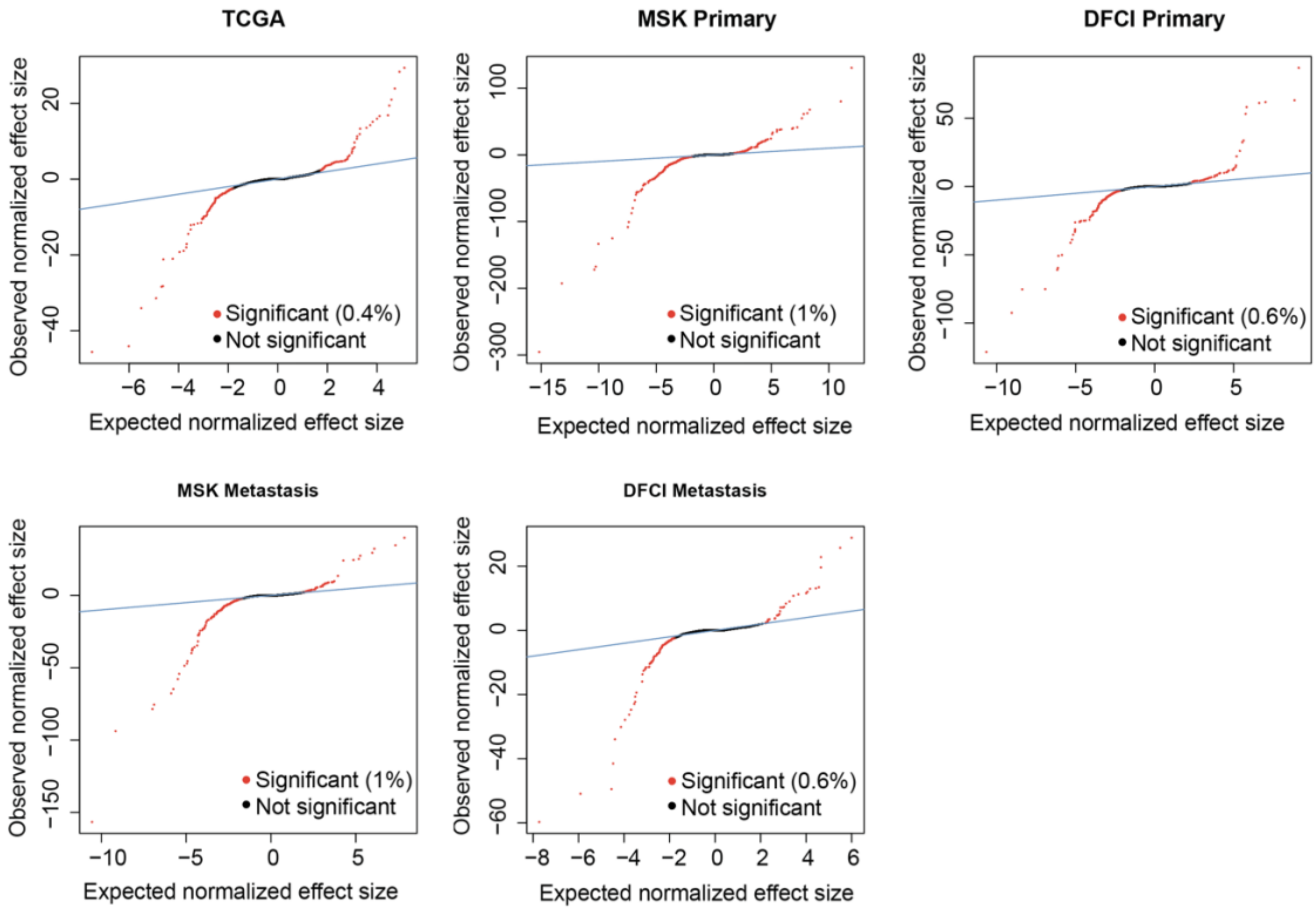

Figure R1: Each scatterplot compares the distribution of expected normalized effect size (X-axis) with those of the observed normalized effect sizes (Y-axis) for each cohort. Black points indicate non-significant co-mutations, red points indicate significant co-mutations. The blue line is the bisector (y=x).

Reviewer #2 (Remarks to the Author):

Response to "We would appreciate your comments on the revision, the degree to which the authors have addressed the concerns of the referees, and whether the manuscript now warrants publication in Nature Genetics".

In this reviewer's view the authors have addressed the concerns of the referees. As with the previous review, the authors diligently performed a few extra analyses in order to provide a proper response to the reviewers' inquiries.

A couple of comments with this regard:

- Reviewer #1 addresses an interesting question in this second round that could well be part of a supplementary note, namely, the potential confounding of the sample purity and VAF of the mutations, which I honestly overlooked in the previous rounds.
- It is entirely up to the editors to decide what is the best way in which the article would deliver all the necessary information so that the article can be read and processed as a consistent body of scientific knowledge on its own with as little burden for the reader as possible. But in this reviewer's opinion, some of the analyses provided by the authors during the full review process are relevant and interesting enough to be part of a supplementary note that can be read and searched as a coherent piece of information.
- In particular, I think that it would be beneficial for the readership to keep all the analyses (and discussions thereof) testing potential confounding effects and artifacts of the method, both provided originally by the authors and raised by the reviewers, in a single document.
- This document could include: 1) the mentioned analysis about VAF and purity; 2) the potential confounding of unequal sparsity raised by Reviewer #2 in the first round of comments; 3) the double-hit vs single-hit analysis raised by Reviewer #2; 4) all the sanity checks and deconfounding of the metastasis risk prediction in regards with co-mutations.

We thank the reviewer for their positive and supporting feedback. The suggestion to include these additional analyses in a Supplementary Note is welcome and we now provide such document covering the four chapters mentioned by the reviewer. We hope this additional report will further demonstrate the robustness and value of our work.

Response to "We would also ask you to inform us of any papers that have appeared in the literature that might compromise the novelty of these results".

Methodologically speaking I found the method compelling and convincingly more reliable than previous widely adopted approaches in the cancer genomics community. Co-mutation analysis methods continue to be published resorting to different modeling ideas e.g. https://www.biorxiv.org/content/10.1101/2025.05.27.656485v1 which focuses on mutual-exclusivity or https://www.biorxiv.org/content/10.1101/2024.04.24.590995v1, both of which are methodologically sound. There are a few additional preprints and conference

proceedings whereby graph-based models e.g. https://assets-eu.researchsquare.com/files/rs-3270626/v1_covered_9a83591b-3116-41c7-b549-4a31ea4d2eeb.pdf?c=1698204149 and language-transformer models are put to the task. I have not seen any compelling proof that these methods perform up to state-of-the-art standards in terms of sensitivity and false discovery rate. In this reviewer's opinion the level of evidence that Iyer et al. provide manuscript is convincing. I am not aware of any peer-reviewed, published papers that might compromise the novelty of these results.

With that said, the analyses that the manuscript addresses are novel. The co-mutation analysis in the manuscript normal tissues is new. On the other hand, there are many studies addressing differential co-mutations and gene clusters in relation with metastatic progression, none to my knowledge are comparable in terms of the extensive, systematic pan-cancer inquiry. Compare for example with other previous pan-cancer studies addressing genomic features of primary to metastasis progression e.g. https://www.nature.com/articles/s41586-023-06054-z where co-mutation drivers are not explored.

We thank once more the reviewer for highlighting the novelty and scientific relevance of our study even in light of the most recent literature.
